# A Window into Domain Amplification Through Piccolo in Teleost Fish

**DOI:** 10.1534/g3.112.003624

**Published:** 2012-11-01

**Authors:** Michael L. Nonet

**Affiliations:** Department of Anatomy and Neurobiology, Washington University School of Medicine, St. Louis, Missouri 63110

## Abstract

I describe and characterize the extensive amplification of the zinc finger domain of Piccolo selectively in teleost fish. Piccolo and Bassoon are partially functionally redundant and play roles in regulating the pool of neurotransmitter-filled synaptic vesicles present at synapses. In mice, each protein contains two N-terminal zinc finger domains that have been implicated in interacting with synaptic vesicles. In all teleosts examined, both the Bassoon and Piccolo genes are duplicated. Both teleost *bassoon* genes and one *piccolo* gene show very similar domain structure and intron-exon organization to their mouse homologs. In contrast, in *piccolo b* a single exon that encodes a zinc finger domain is amplified 8 to 16 times in different teleost species. Analysis of the amplified exons suggests they were added and/or deleted from the gene as individual exons in rare events that are likely the result of unequal crossovers between homologous sequences. Surprisingly, the structure of the repeats from cod and zebrafish suggest that amplification of this exon has occurred independently multiple times in the teleost lineage. Based on the structure of the exons, I propose a model in which selection for high sequence similarity at the 5′ and 3′ ends of the exon drives amplification of the repeats and diversity in repeat length likely promotes the stability of the repeated exons by minimizing the likelihood of mispairing of adjacent repeat sequences. Further analysis of *piccolo b* in teleosts should provide a window through which to examine the process of domain amplification.

Multidomain proteins are very common in eukaryotes and exon shuffling during evolution has been proposed as a primary mechanism for the creation of new multidomain protein architectures (Gilbert 1987; Patthy 1999). It has also been recognized that gene duplication, and in particular whole-genome duplications (WGDs), likely had a great impact on the evolution of protein families by providing a period during which such exon shuffling was much less constrained (Wolfe and Li 2003; Davis and Petrov 2005; Semon and Wolfe 2007). In addition to exon shuffling, amplification of repeated domains has also contributed to the diversification of protein architecture, and such domain amplification has been particularly pervasive in multicellular vertebrate evolution (Bjorklund *et al.* 2006). In the new era of whole-genome sequencing, the potential to examine these evolutionary events in more detail has become tantalizingly feasible. Furthermore, in some species experimental manipulation of the organism may provide the ability to experimentally test certain predictions of models for mechanisms driving protein structure diversification.

Teleosts provide a potentially rich source of data to address these questions at a bioinformatics level and potentially at an experimental level. A WGD occurred early in the teleost lineage and it is estimated that approximately 80–85% of duplicated genes have been lost in the species that have been examined in molecular detail (Jaillon *et al.* 2004; Brunet *et al.* 2006). Molecular analysis of distinct teleost species has revealed the extensive amplification of specific gene families such as the MHC I complex in cod (Star *et al.* 2011) and the TRIM protein family in zebrafish and salmonids (Van der Aa *et al.* 2009). The vast number of teleost species should provide a vast wealth of sequence data to more precisely define how these unique amplification of gene families occur.

Domain expansion within proteins has been less extensively studied, although it likely has also played a significant role in expanding the diversity of genes in vertebrate evolution (Bjorklund *et al.* 2006). The utility of teleost diversity in addressing this problem has been much less well examined. Herein, I describe the unusual amplification of a zinc finger encoding exon in the Bassoon/Piccolo family of active zone proteins in teleosts. I characterize the structure of the Bassoon and Piccolo genes in multiple teleosts by using available whole-genome sequence (WGS) data. I demonstrate that a zinc finger−encoding exon in one of the two Piccolo genes has expanded from one copy to 8 to 16 copies in different teleost species. I characterize the relationship of these exons and propose a model for the amplification of the exons in the teleost lineage. The features of this domain amplification in teleosts provide a unique and potentially powerful model to understanding mechanisms driving and limiting domain duplication.

## Materials and Methods

### DNA sequencing of cod, tilapia, and green spotted puffer zinc finger repeats

I obtained a cod (*Gadus morhua*) from a local fish market, a green spotted puffer (*Tetraodon nigroviridis*) from a local tropical fish market, and tilapia genomic DNA from Andrew Stuart at the Benaroya Research Institute. Genomic DNA was isolated from brain (cod) or fin tissue (spotted puffer) using a standard protocol: tissue was digested in 10 volumes of 10 mM Tris, 100 mM ethylene diamine tetra-acetic acid (EDTA) pH 8, 0.5% sodium dodecyl sulfate, 200 µg/mL of proteinase K for several hours at 50°, extracted 2 times with phenol, once with phenol-choroform, and once with choroform, precipitated in 2 volumes of ethanol after the addition of NaCl to 300 mM, and resuspended in TE (10 mM Tris 1mM EDTA, pH 7.5). RNA was removed by RNAse A digestion, followed by a second round of phenol extraction and precipitation. The final DNA was resuspended in TE. To molecularly confirm the species identity of the cod and spotted puffer, I used polymerase chain reaction (PCR) to amplify (see supporting information, Table S3, for a list of all primers) and sequence the D-loop control region of the mitochondrial genome from the specimens. Repeat sequences described herein have been deposited into the EMBL/GenBank Data Libraries under accession nos. JX535022-JX535026.

In the case of tilapia, zinc finger repeat 9 was sequenced resulting in the modification of a 123-bp segment that included 28 N, with an 84-bp sequence in lower case: CCTGATTCAAAGACAGAAAAGGCACCCCTTcaacagcctccaaaagctgcagcttctttagctaagtctcctcttccatcagaccaagaagcaagaaagccacccccacaacagCCTCCAAAAGCTGCAGCTTCTTTAGCTAAG.

In the case of green spotted puffer, the end of zinc finger repeat 2 and beginning of repeat 3 were not covered by the WGS. PCR-amplified sequences representing this gap were sequenced, resulting in the addition of coding sequences in italics and intronic sequences in lower case: GTGGATTTAACCCCATGCCAAACATTACAGA*G*gtaagatcttaaccataaaatatacactattattattattattatatttttttacaatttctctcatttgttttttgattttctaacataaag*GTGAAGGAGTGGCTTTGTCTAAACTGCCAGATGCAGAGAGCACTAGGATCATCTGAACCTCCAGGAACTCCAGCAGCAAAGCTTCAGGCTTCCCC*AAATAGAGTGAGCACCCCTGCTAGTACCCCAAAGAAGGAATTCTCTCAGTTAGATCAGTCTCGAAAG.

In addition, spotted puffer repeat 4 was PCR-amplified and sequenced, resulting in the correction of a frame shift by removal of an A (in lower case) present in the WGS AACACCTGCACTGAGTGCAAGACCAaTCGTCTGCACTCAGTGTGGATTT. Finally, PCR amplification and sequencing of spotted puffer repeat 8 resulted in the correction of three frame shift errors due to bases absent from in the WGS (underlined) and three nucleotide substitutions (in lower case):AACACCACCGACTCCACGTAAGATGTCTGCCGCAGGGCACGTCTCACCTAAAACTACACCGCCTGCCTCTCCTAGGTCATTACCTGTtaAGgACACCAAGCCTTTTAAAACCGAGGAGAAGACGCCAGTGCAATTACAGCAGGCTCCGGTGACAGCACAAGCTGAGAGAGAAAAAGATCCAGCAGAGAAAGCCAAGGCACCAACAGACAAACAAGACCTATCCATCTGTCCACTCTGTAAAGGTCGACTCAACTTGGGCTCCAAAGATCCTCCTAACTACAACACTTGCACACAGTGCAAGTCGACTGTCTGCAGCCAGTGTGGATTTGATCCAAAACCAAACGTGATTGAG

In the case of cod, zinc finger repeat 13 was amplified and sequenced, leading to the removal of a lowercase g from the WGS sequence: CAGAAATCTCCAGACCTGACCAATCAAACTgGAACGAAAGCAAAGCACCCAACAGGAGTCT.

Two other modifications to the cod *piccolo b* WGS were performed to restore the reading frame of *piccolo b* before phylogenetic analysis, although these changes were not confirmed by sequencing because they are in highly conserved regions and occurred in G or C repeats that are often misread in high-throughput sequencing. First, a C (lowercase) was removed from the sequence CAACCAAGGCCAGGCCCTGGGCCTGGGCCCCcCCATGCGGTCCTCCGTCCAAGACGACGGG and two Gs (lowercase) were removed from the sequence: CGTGACCGGAGCAGGGCCCCAGAGCCAGGggGGCTGGGCGTGCTCTCTGCCCTGGAGCGCTCC.

In the case of the cod *piccolo a* gene, one modification was made to restore the reading frame of the gene. Specifically, a C was removed from the sequence, CAGACGGCGTACACCACCGGCTCCGCCCGAcCGCCGGATGTGCCGCAACTCCAACCTGGCC, without confirmation by sequencing.

In assembling the zebtrafish *picollo b*, an error was identified in coding region of the large exon 6190 bp exon (6189 in Zv9). Specifically, a C was added to correct a reading frame error in the sequence agatgatcaacagccattaagaaaggCctcaagaaaaatgaagactgaaaaag. Evidence for this includes the presence of this C in both the AB and Tuebingen strain WGS Illumina contigs of this region (available by BLAST at http://www.sanger.ac.uk/resources/software/blast/) as well as our own sequencing of this region from a PCR product derived from cDNA.

In assembling the zebrafish *bassoon* genes, I used the data from the Illumina WGS assemblies of the AB and Tuebingen strains to fill a few gaps in the Zv9 genome. These additions are described in Table S2.

Zebrafish *bassoon b* was incorrectly annotated in Ensembl as two genes ENSDARG00000086319 (which encodes the first 3 exons of *bassoon b*) and ENSDARG00000079161 (which encodes the remainder of the gene) likely because in the Zv9 version of the genome the two portions of the gene are separated by a 300-kb interval containing several genes. I confirmed these two genes are one by reverse transcription PCR using oligonucleotide in the 3′ and 5′ of the respective genes (see Table 3). Sequencing of the resulting 1.1-kb cDNA fragment confirmed the *bassoon b* gene structure we describe herein (GenBank accession no. JX560813).

### Reverse-transcription PCR analysis of zebrafish *piccolo b* transcripts

Total RNA was isolated from 5-day-old zebrafish embryos. Fifty embryos were anesthetized in 0.04% tricaine methanesulfonate, frozen, and thawed mixed with 250 µL of Trizol reagent (Invitrogen), incubated for 15 min at room temperature until no solid material was visible, one-fifth a volume chloroform was added, vortexed for 15 sec, incubated 3 min at room temperature, and centrifuged at 12,000*g* for 15 min at 4°. The aqueous upper layer was precipitated by the addition of half a volume of isopropyl alcohol and pelleted by centrifugation at 12,000*g* for 10 min at 4° after a 15-min incubation. The resulting pellet was with 75% ethanol and resuspended in water. cDNA was synthesized from total RNA using Superscript III (Invitrogen) reverse transcriptase following the manufacturer’s instructions in two separate reactions; one primed with oligo-dT and one with random N_6_ primers. A mixture of the two first strand cDNA reactions was used for PCR analysis of *piccolo b* transcripts using oligonucleotides listed in Table S3.

### *In situ* hybridizations

*In situ* hybridization of brain tissue sections were performed essentially as described by VanDunk *et al.* (VanDunk *et al.* 2011). PCR products were used as templates for probe synthesis and amplified from cDNA using oligonucleotides listed in Table S3. Digoxigenin (DIG)-labeled antisense and sense RNA probes were synthesized from PCR products using DIG-labeled nucleotides (Roche) and T3 or T7 RNA polymerases (Promega). cRNA probes were purified using Quick Spin columns (Roche) and quantified by spectrophotometry. Probes were used at a concentration of 1–2 μg/mL.

Brains were removed from anesthetized (in 0.04% tricaine methanesulfonate) adult wild-type zebrafish and fixed overnight in 4% paraformaldehyde (PFA) in 0.1 M phosphate-buffered saline (PBS), cryoprotected in 20% sucrose in PBS, frozen in O.C.T. Compound Embedding Medium (Tissue-Tek), and stored at −75°. Then, 20-μm sections were cut on a Hacker cryostat and collected on superfrost plus slides (Fisher Scientific) and air-dried overnight. Slides were immersed in 4% PFA, permeabilized with 10 µg/mL proteinase K, and returned to 4% PFA before being washed in 0.1 M triethanolamine-HCl with 0.25% acetic anhydride. Slides were subsequently blocked for 4 hr in saline sodium citrate (SSC)-based hybe buffer [50% formaldehyde, 5× SSC (0.75M NaCl, 75 mM Na_3_ citrate, pH 7.0), 0.3 mg/mL yeast tRNA, 0.1 mg/mL heparin, 0.02% bovine serum albumin, 0.02% polyvinylpyrolodone, 0.02% Ficoll 400, 0.1% Tween 20, and 5 mM EDTA] at 65°, then incubated in hybe buffer containing 1–2 μg/mL DIG-labeled antisense cRNA overnight at 65°. Slides were then washed in 2× SSC at 62°, washed in 0.2× SSC at 65°, blocked with 10% normal horse serum in 0.1 M PBS, and incubated in alkaline phosphatase-labeled anti-DIG antibody (1:2000 in 10% normal horse serum; Roche) overnight. Sections were washed and color was visualized using nitro blue tetrazolium and 5-bromo-4-chloro-3-indolyl phosphate (Roche). Staining was stopped after visual inspection. Sections were washed, fixed in 4% PFA, and cover slipped in 90% glycerol, Vectashield Mounting Medium (Vector Laboratories).

### Phylogenic analysis

Evolutionary analyses were conducted in MEGA5 (Tamura *et al.* 2011). Piccolo and Bassoon protein sequences were aligned using MUSCLE (Edgar 2004a,b) with default settings (−2.9 penalty for gap opening, no penalty for gap extension, a 1.2 hydrophobicity multiplier, and UPGMB clustering method). The nucleotide sequence of this alignment was used to determine a phylogenetic tree using a neighbor-joining method (Saitou and Nei 1987). One-thousand bootstrap replicates were used to assess the support for the tree. All positions with missing data were eliminated, and all codon positions were used in the analysis for analysis of the Piccolo and Bassoon whole genes.

Aligning the zinc finger exons in complicated by the extensive diversity in length of these exons despite the presence of significant conserved regions. For zinc finger exon alignments, MUSCLE was used but the gap-opening penalty was increased to −4.9 and gap extension to −0.01. This was done to minimize the number of gaps introduced in the alignments. Trees for the zinc finger exons were performed similarly those mentioned previously except that only position with missing data in greater than 50% of the sequences were eliminated from the analysis. In some figures branch lengths were added and are in units of nucleotide substitutions per base. In most trees, all branches with less that 50% bootstrap support were collapsed.

### Sequence alignments presentation

All alignments were exported from MEGA5 in fasta format, and imported into CLUTALX 2.09 to create color-coded PDF alignment files for presentation using the default color-coding parameters. In summary, shading is as follows: glycine residues (G) orange and proline residues (P) yellow; conserved cysteine residues (C) pink, conserved acidic residues aspartate (D) and glutamate (E) magenta; conserved basic resides lysine (K) and arginine (R) red; conserved polar residues serine (S), threonine (T), asparagine (Q), and glutamine (N) green; conserved tyrosine (Y) and histidine residues (H) dark blue; and conserved hydrophobic residues phenylalanine (F), tryptophan (W), alanine (A), leucine (L), valine (V), isoleucine (I), and methionine (M) light blue. In some alignments, a bar graph below represents the percent conservation at individual sites in the alignment. In some alignments, asterisks, colons, or periods are present above the alignment; asterisks represent 100% conserved residues, colons represent 100% conservation of a strong amino acid (aa) substitution group (*e.g.*, acidic, basic, hydrophobic), and periods represent a 100% conserved weaker substitution group (*e.g.*, alanine, threonine, or valine). See Clustal X documentation for details (Larkin *et al.* 2007).

## Results

### One gene in teleosts is slightly divergent from mammalian Piccolo

**Figure 1:**
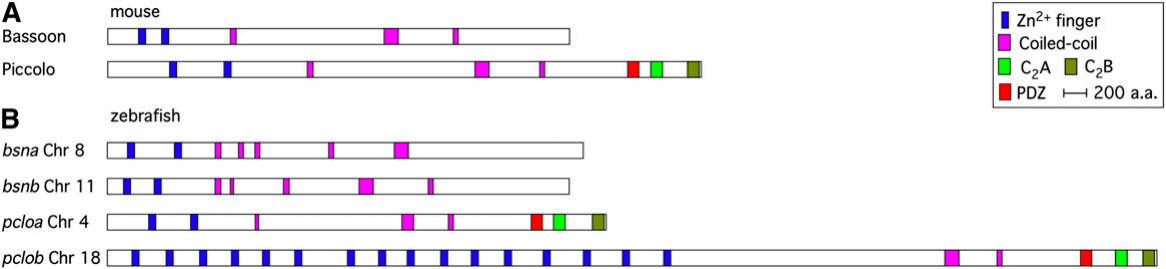
Piccolo and Bassoon protein structure in mouse and zebrafish. A schematic diagram of the domain structure of Bassoon and Piccolo proteins in (A) mouse and (B) zebrafish as determined in this work. Domains were identified using SMART (Letunic *et al.* 2012) and are color coded as denoted in the figure.

**Figure 2:**
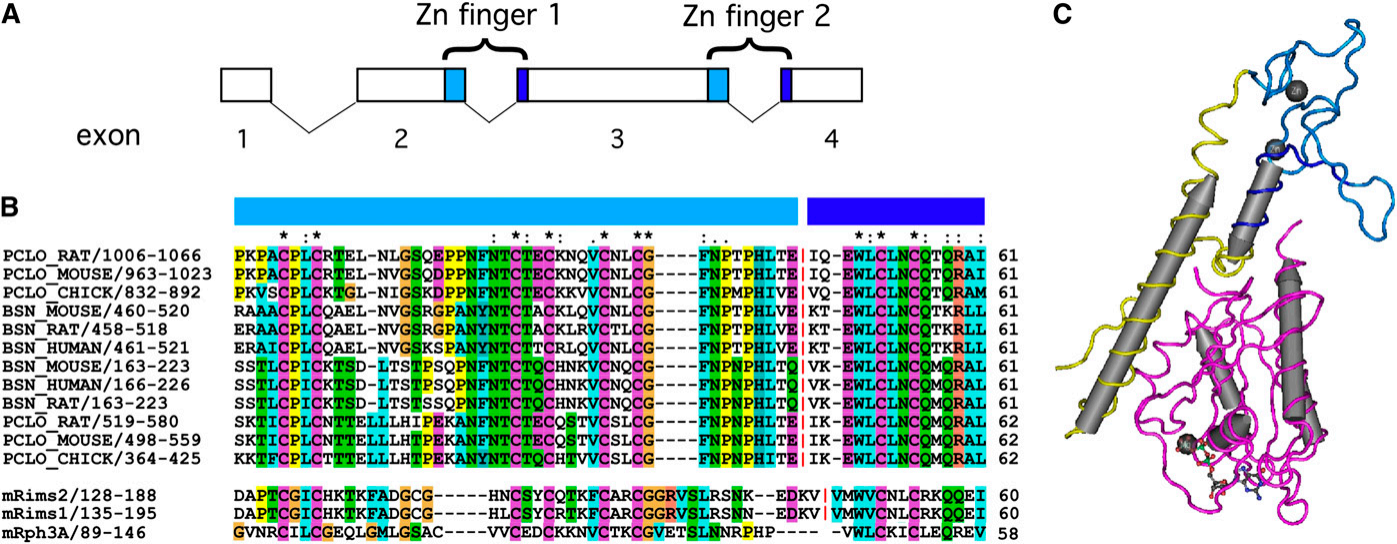
Structure of the zinc finger domains of Piccolo and Bassoon. The zinc finger domains of both mouse Piccolo and Bassoon are encoded in the first four exons of each gene as illustrated in (A). The two zinc fingers in each case are encoded across the exon 2|3 and exon 3|4 boundaries. (B) Alignment of assorted vertebrate Piccolo and Bassoon zinc finger domains illustrating conservation in the position of the exon boundary both between the two zinc fingers and between the two genes. The first 3 CxxC motifs of the domain are encoded in the 3′ of exons 2 and 3 (light blue), and the last CxxC motif is encoded in a smaller conserved region in the 5′ of exons 3 and 4 (dark blue). The position of the exon boundaries is marked with a red vertical line (|). The sequence of the related RIM and Rabphilin zinc finger domains are also included. (C) The crystal structure of the evolutionarily related zinc finger domain of rabphilin that binds selectively to the GTP-bound form of the small G-protein Rab3 (Ostermeier and Brunger 1999). Note that the zinc finger domain (light blue and dark blue) itself does not bind directly to Rab3 (pink backbone), but rather coordinates the positioning of alpha helices N-terminal (yellow) and C-terminal of the zinc finger domain. Light blue and dark blue-labeled portions of the sequence in B are represented by light blue and dark blue colored backbone in (C). Color-coding of aa is described in materials and methods.

Bassoon and Piccolo (aka Aczonin) are two very large (>450 kD) structurally related proteins that were first identified as components of the cytomatrix scaffold associated with the active zone at vertebrate central nervous system synapses (tom Dieck *et al.* 1998; Wang *et al.* 1999; Fenster *et al.* 2000). Both proteins are widely expressed in the central nervous system and localized to virtually all presynaptic specializations. Mice lacking Bassoon or Piccolo are viable, but each mutant displays some abnormalities, including epileptic seizures in Bassoon mutants and reduced postnatal viability and body weight in Piccolo mutants (Altrock *et al.* 2003; Mukherjee *et al.* 2010). Analysis of mouse cultured neurons with both Piccolo and Bassoon function disrupted revealed that the proteins are required for maintaining normal populations of synaptic vesicles at synapses (Mukherjee *et al.* 2010). Structurally, each consists of two N-terminal zinc finger domains and an extended coiled coil region (Figure 1 and Figure 2). In addition, Piccolo contains a C-terminal extension consisting of a PDZ domain and two C2 domains. Although Bassoon and Piccolo are unique in their large size, similar zinc finger, PDZ and C2 domains are also found in several other proteins localized to the presynaptic cytomatrix, including the proteins Rims1 and Rims2α (Wang *et al.* 1997, 2000; Jin and Garner 2008). However, herein I focus on Bassoon and Piccolo.

**Figure 3:**
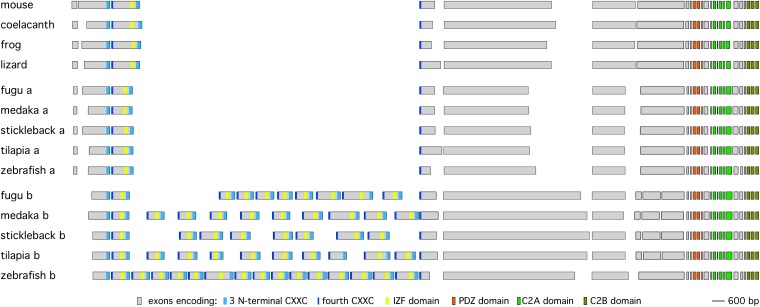
Intron-exon structure of teleost *piccolo* genes. A diagram illustrating the exon structure of *piccolo* genes from various teleost and non-teleost vertebrates. The mouse and zebrafish exon structures are supported by cDNA and/or RNAseq data. In the case of frog, coelacanth, lizard, fugu, medaka, stickleback, and tilapia, the intron-exon structure was deduced by manual annotation from WGS data using homology and splice consensus sequences as guides. The exons are presented to scale, but the introns are not. Exons were positioned such that those encoding homologous regions are aligned at the 5′ end of the exon. The position of various domains is color coded.

I initially searched for mouse Piccolo homologs in zebrafish (*Danio rerio*) using BLAST against the Zv9 version of the genome. Two Piccolo homologs were identified, one on chromosome (chr) 4 and one on chr 18. The genomic organization of the gene on chr 4, named *piccolo a (*symbol *pcloa)*, was determined manually using homology and splice-site consensus sequences as bioinformatic guides and subsequently the intron-exon structure was confirmed by RNAseq data in the v64 of Ensembl (Flicek *et al.* 2012). Zebrafish *pcloa* has the potential to encode a 4259 aa protein sharing 49% amino acid identity to mouse Piccolo (Figure 1). Furthermore, the intron-exon structure of *pcloa* was identical to that of the mouse gene (Figure 3 and Table S1), and even intron size correlated well between the two homologs (Figure S1).

### Zebrafish *pclob* has 16 zinc finger domains rather than two

Unexpectedly, the Piccolo homolog on chr 18, named *piccolo b* (symbol *pclob*), was configured with a very distinct organization of zinc finger domains from the mouse Piccolo gene. In mouse Piccolo and zebrafish *pcloa*, as well as all of the non-teleost Piccolo homologs I examined, the two zinc finger domains are distributed across 3 exons (Figure 2A and 3). Each zinc finger consists of 4 di-cysteine CXXC motifs in a ∼60 aa domain (Figure 2B). In mouse Piccolo each zinc finger domain spans an intron-exon boundary with the first 3 CXXC motifs located in the 3′ end of an exon (Figure 3), and the last CXXC motif located in the 5′ of the subsequent exon. Thus, in each gene a single “central” exon (exon 3 in mouse Piccolo and zebrafish *pcloa*) encodes both the last CXXC motif of the first zinc finger on the 5′ boundary of the exon and the first 3 CXXC motifs of the second zinc finger on the 3′ boundary of the exon. By contrast, analysis of the genomic region of zebrafish *pclob* (in Zv9) revealed 15 exons with similar structure in tandem (Figure 3). In addition to the highly conserved zinc finger CXXC motifs at the 5′ and 3′ of each exon, there was also a highly conserved inter zinc finger (IZF) domain of 22 aa in the middle of each exon (discussed later). In zebrafish *pclob* the exons ranged in size from 702 bp to 1350 bp, but similarly to the “central” exon 3 of all the non-teleost vertebrates I examined and zebrafish *pcloa*, they all started and ended in frame 0. By contrast, the remainder of the *pclob* gene was organized with identical domain structure and identical intron-exon structure as mouse Piccolo (Figure 3). Thus, zebrafish *pclob* is organized such that it has the potential to encode a protein very similar to mouse Piccolo, but with 16 N-terminal zinc fingers, rather than 2 zinc fingers.

Although zebrafish *pclob* exhibits multiple additional “central” exons compared with the mouse Piccolo gene, these additional exons could be spliced in different ways during processing of the primary *pclob* RNA transcript. One possibility is that these 15 exons represent a set of alternative “central” exons allowing for a set of 15 similar, but distinct, two zinc finger domain Pclob isoforms to be expressed. On the other hand, all exons might be used producing a Pclob protein with 16 zinc fingers with almost twice the mass of mouse Piccolo. To distinguish between these possibilities, I used oligonucleotides designed to hybridize specifically to individual repeats and performed PCR amplifications from first strand cDNA synthesized from RNA isolated from 5-day-old zebrafish embryos. Because the exons are large, I did not attempt to amplify across the entire set of 15 exons (13,497 bp), but rather amplified across pairs of exons. These results demonstrate that each exon is spliced into mature transcripts (Figure S2). I only detected the skipping of a single central exon (the 14th), and this product was much less abundant than then the larger splice product containing the exon (Figure S2B). Independently, I analyzed RNAseq data in v64 of Ensembl. Similar to the PCR analysis, the RNAseq data indicated that the gene was highly expressed in both 14-day-old male and female heads, consistent with the previously documented neuronal function of mouse Piccolo (Figure S3). Furthermore, in these samples, all adjacent exon splice products were present in approximately equal abundance, and few exon skipping products were present (although in other tissue samples some evidence of exon skipping was found). These data suggest that the primary mature RNA transcript of zebrafish *pclob* in neurons is predicted to encode an 9009 aa Piccolo-related protein with 16 zinc fingers, a PDZ domain and two C2 domains (Figure 1B).

### One dual zinc finger and one multizinc finger in each sequenced teleost genome

Finding the structure of zebrafish *pclob* unusual, I analyzed the genomic structure of Piccolo-related genes of multiple other teleosts as well as coelacanth, lizard, and frog. In most cases, the annotation of the genes was incorrect, and thus I manually annotated each gene using the mouse and zebrafish genes as guides (because the mRNA structure of these genes is known). In the case of tilapia, cod, and green spotted puffer, specific primers were used to amplify small regions from the Piccolo genes to correct sequencing errors and fill small gaps in the available genomic sequence (see *Materials and Methods* for details). I only identified a single Piccolo homolog in coelacanth, lizard, and frog, and each of these genes exhibited identical domain structure and intron-exon structure to the mouse Piccolo and zebrafish *pcloa* genes (Figure 3, Table S1, and data not shown). In contrast, the stickleback, fugu, green spotted puffer, medaka, tilapia, and cod genomes all contained two Piccolo homologs. In each case, one ortholog exhibited the same domain and intron-exon structure as zebrafish *pcloa* and Piccolo. Furthermore, in each case, the zinc finger domain was greatly amplified in the other ortholog, although the genes from different species contained different numbers of “central” exons varying in number from 8 to 16 (Figure 3). Apart from the additional zinc finger “central” exons, the domain structure of these Piccolo related genes resembled mouse Piccolo and zebrafish *pcloa*. The only exceptions were two additional introns present in the identical position in a large exon just before the PDZ domain encoding exon present in all the teleosts other than zebrafish (Figure 3). Thus, all teleosts, and only the teleosts among all the vertebrate genomes I examined, appear to contain a second Piccolo related gene with multiple additional exons encoding zinc finger domains.

### Bassoon is duplicated without zinc finger domain expansion in teleosts

**Figure 4:**
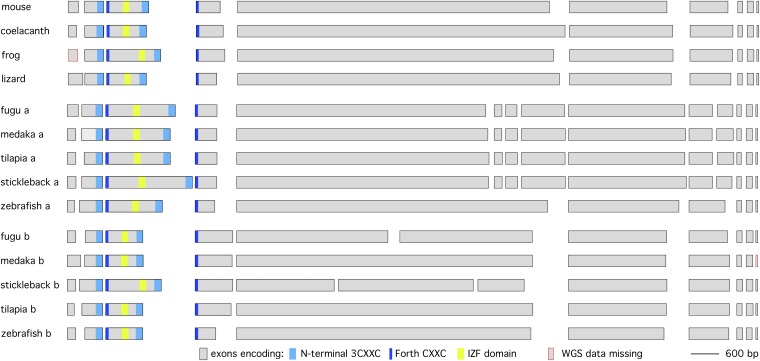
Intron-exon structure of teleost *bassoon* genes. A diagram illustrating the exon structure of *bassoon* genes from various teleost and non-teleost vertebrates. The mouse and zebrafish exon structures are supported by cDNA and/or RNAseq data. The exons are presented to scale, but the introns are not. The intron-exon organization of all other genes was deduced by manual annotation from WGS data using homology and spice consensus sequences as guides. Exons were positioned such that those encoding homologous regions are aligned at the 5′ end of the exon. The position of various domains is color coded. Data missing from WGS are marked in red.

Piccolo is closely related to Bassoon, and functional analysis of these genes in mouse has revealed that they are at least in part functionally redundant (Mukherjee *et al.* 2010). In particular, both Bassoon and Piccolo proteins have two N-terminal zinc finger domains and an extended region with coiled coil character and the portion of both genes that code for these domains exhibit similar intron-exon organization. Thus, Bassoon appears to be a Piccolo-like gene lacking the PDZ and C2 domains (or vice versa). Thus, I searched the coelacanth, lizard, frog, and various teleost genomes for homologs of mouse Bassoon. Because the Bassoon homolog was incorrectly annotated in most species, I manually annotated each gene, and confirmed the manual annotation using the v64 Ensembl RNAseq data for the zebrafish homologs. The zebrafish Bassoon homologs found on chr 8 and chr 11 were named *bassoon a (*symbol *bsna)* and *bassoon b (*symbol *bsnb)*, respectively. As is the case for Piccolo, I found two Bassoon homologs in each teleost genome I analyzed, and one Bassoon homolog in coelacanth, frog, and lizard. However, by contrast with Piccolo, the two Bassoon homologs in each teleost showed identical domain structure to mouse Bassoon and very similar intron-exon structure (Figure 4 and Table S2). The two homologs were slightly divergent, which was reflected by small differences in intron-exon boundaries of the two homologs outside the zinc finger domain region. Thus, despite having very similar structural zinc finger domains and intron-exon boundary organization, Bassoon zinc finger domains have not amplified in number in teleosts.

### Both *bassoon* and *piccolo* genes broadly expressed in adult brain

The unusual structure of *pclob* homologs raises the possibility that this gene is adapted for a highly specialized function in teleosts, which might be revealed via restricted expression in specific cell types. To address this possibility, I analyzed the expression pattern of all the zebrafish *piccolo* and *bassoon* genes in adult brain using RNA *in situ* hybridization and used the glutamic acid decarboxylase gene as a positive control. To ensure the probes were specific for individual genes the probes were designed to anneal to the most divergent region of the proteins, a region after the zinc finger domain. The resulting ∼850- to 950-bp probes shared only small 100-200 bp regions with limited homology (<75% identity) with the closest homolog. I found that all four zebrafish genes were broadly expressed in a wide variety of regions of the adult brain (Figure S4). Both *pcloa* and *pclob* were expressed in the cerebellum and the torus longitudinalis just below the optic tectum, but *pcloa* was more widely expressed in telencephalic regions, whereas *pclob* was more broadly expressed in hindbrain regions. *bsna* and *bnsb* expression overlapped to an even greater degree with both being broadly expressed in forebrain, although *bsna* appeared less robustly expressed in the cerebellum and the torus longitudinalis. In summary, both of the duplicated *piccolo* and *bassoon* genes in zebrafish are broadly expressed in adult brain.

### *bassoon* and *piccolo* genes duplicated after divergence of teleosts from mammalian lineage

I first aimed to establish the evolutionary relationship of the two *piccolo* and two *bassoon* gene families found in teleosts. To do so, I removed the sequences coding for the N-terminal region and zinc finger domains (which are divergent in structure in the *piccolo* genes) and aligned the remaining portion of the genes using MUSCLE (Edgar 2004a,b) and created phylogenic trees using a neighbor-joining approach. As predicted based on strong WGS evidence for a WGD early in the teleost lineage (Kasahara *et al.* 2007), teleost *pcloa* and *pclob* were more closely related to each other than the Piccolo homologs found in the nonteleost lineages (Figure S5 and Figure S6). Similarly, teleost *bsna* and *bsnb* homologs were more closely related to each other than to Bassoon homologs of the non-teleosts I examined (Figure S5 and Figure S7). The resulting tree for Bassoon homologs excluding the zinc fingers was similar to that obtained for the entire Bassoon protein (Figure S8A). The conserved PDZ, C2A, and C2B domains found only in the Piccolo family also showed the same phylogenic relationship, although these were less divergent than the remainder of the Piccolo consistent with these domains having highly conserved roles in Piccolo function (Figure S8B). In addition, as expected based on previous molecular phylogenic studies (Kasahara *et al.* 2007; Setiamarga *et al.* 2009; Star *et al.* 2011), the zebrafish *piccolo* and *bassoon* genes were most distantly related to the other teleost *piccolo* and *bassoon* homologs, followed by the cod genes. The genes of the sequenced percomorph species (medaka, tilapia, stickleback, and fugu and green spotted puffer) were more similar and formed a cluster whose evolutionary relationships could not be unambiguously defined solely using *bassoon* and *piccolo* sequences. In summary, evidence strongly supports the duplication of teleost *piccolo* and *bassoon* after the divergence of the teleost lineage from other vertebrates.

### *pclob* zinc finger number changes by the duplication or deletion of single repeats in percomorphs

I analyzed the repeat structure of the *pclob* genes from the teleost genomes more carefully. Interestingly, the genomic structure of all the *pclob* homologs was similar. Each contained many closely spaced “central” exons followed by a very large intron separating the last central exon from the remaining coding sequences of the gene (Figure S9). Thus, in all teleosts examined the increase in the zinc finger domain number appeared to be most consistent with variable amplification of the “central” exon 3 encoding the C-terminal half of zinc finger 1 and the N-terminal half of zinc finger 2 in the mouse Piccolo gene.

**Figure 5:**
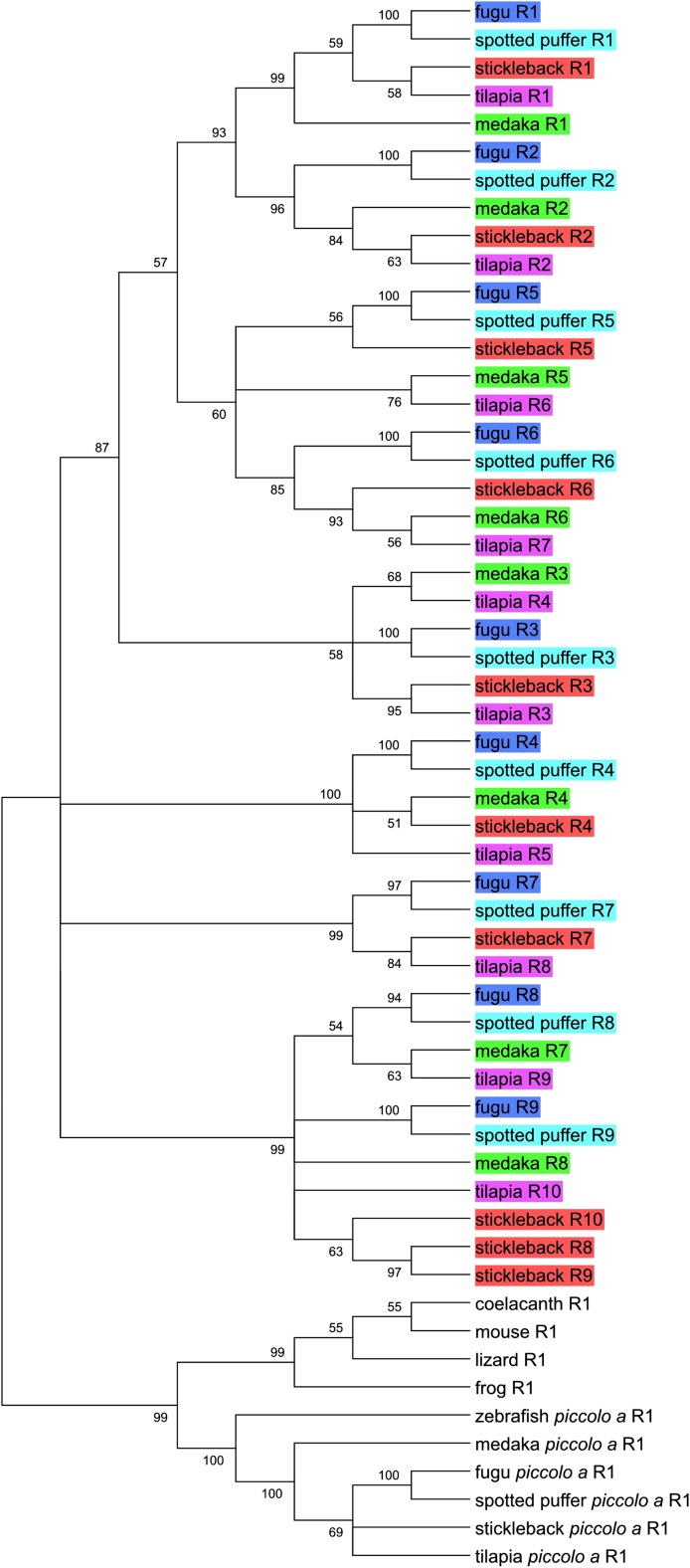
Evolutionary tree of repeated *piccolo* gene exons encoding zinc finger domains. The evolutionary history of the repeat exons was inferred using the neighbor-joining method. Branches corresponding to partitions reproduced in less than 50% bootstrap replicates are collapsed. The percentages of replicate trees in which the associated sequences clustered together in the bootstrap test (1000 replicates) are shown next to the branches. The teleost repeats are color coded by species. All codon positions were included. All positions with less than 80% site coverage were eliminated. There were a total of 801 positions in the final dataset. The alignment used to construct the tree is available in Figure S13.

**Figure 6:**
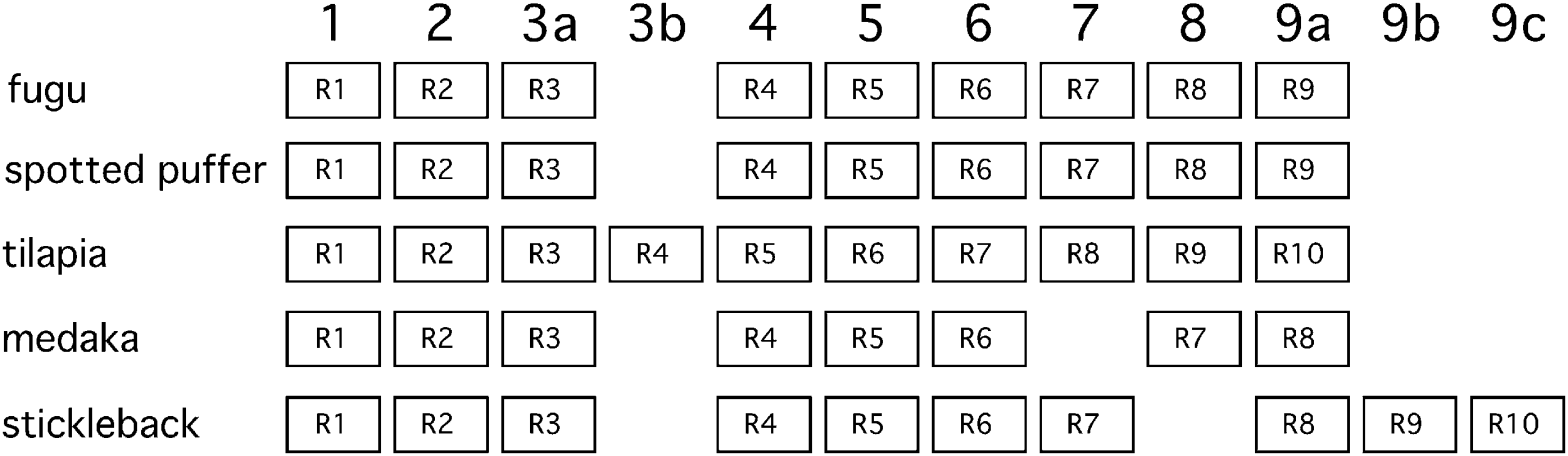
Model illustrating the relationship of the repeated exons encoding zinc finger domains. I propose that *pclob* genes of percomorpha fish diverged from a common ancestor with 9 repeated zinc finger coding exons (with a similar organization to fugu and spotted puffer). The figure depicts the 8 to 10 repeated exons from the percomorpha species analyzed and how they relate to the ancestral repeats labeled in bold above the individual species repeats. For example, the fourth tilapia repeat “R4” is evolutionarily derived from the ancestral repeat 3, and thus in the 3b column.

To address the evolutionary origin of the duplicated zinc finger domains, I performed clustering analysis on the repeated exons by using the single central exon of several other vertebrates to root the tree. Fugu, green spotted puffer, medaka, tilapia, and stickleback all contained *pclob* genes with 8-10 zinc finger repeats and are the most closely related of the sequenced teleosts having diverged within the last ∼190 million years (Steinke *et al.* 2006; Kasahara *et al.* 2007). Thus, I first performed a clustering analysis of sequences from these species. Specifically, I aligned the sequences using MUSCLE and then constructed phylogenic trees using a neighbor joining approach. Analysis of the zinc finger domain tree revealed that analogous exons of each of these species are clustered together in the same branch, and are thus likely to have evolved from a common ancestor. Furthermore, the differences in exon number (and hence exon organization) appear to result from the addition or deletion of single exons (Figure 5). In particular, fugu and spotted puffer appear to represent an ancestral state of 9 repeats whereas medaka has lost repeat 7, tilapia has duplicated repeat 3, and stickleback has both lost repeat 8 and duplicated repeat 9 two times in separate events (Figure 6). The structure of these zinc finger domain exons suggests that zinc finger repeat number changes by rare individual exon duplication or deletion events.

### Multiple repeat expansions in teleost evolution

**Figure 7:**
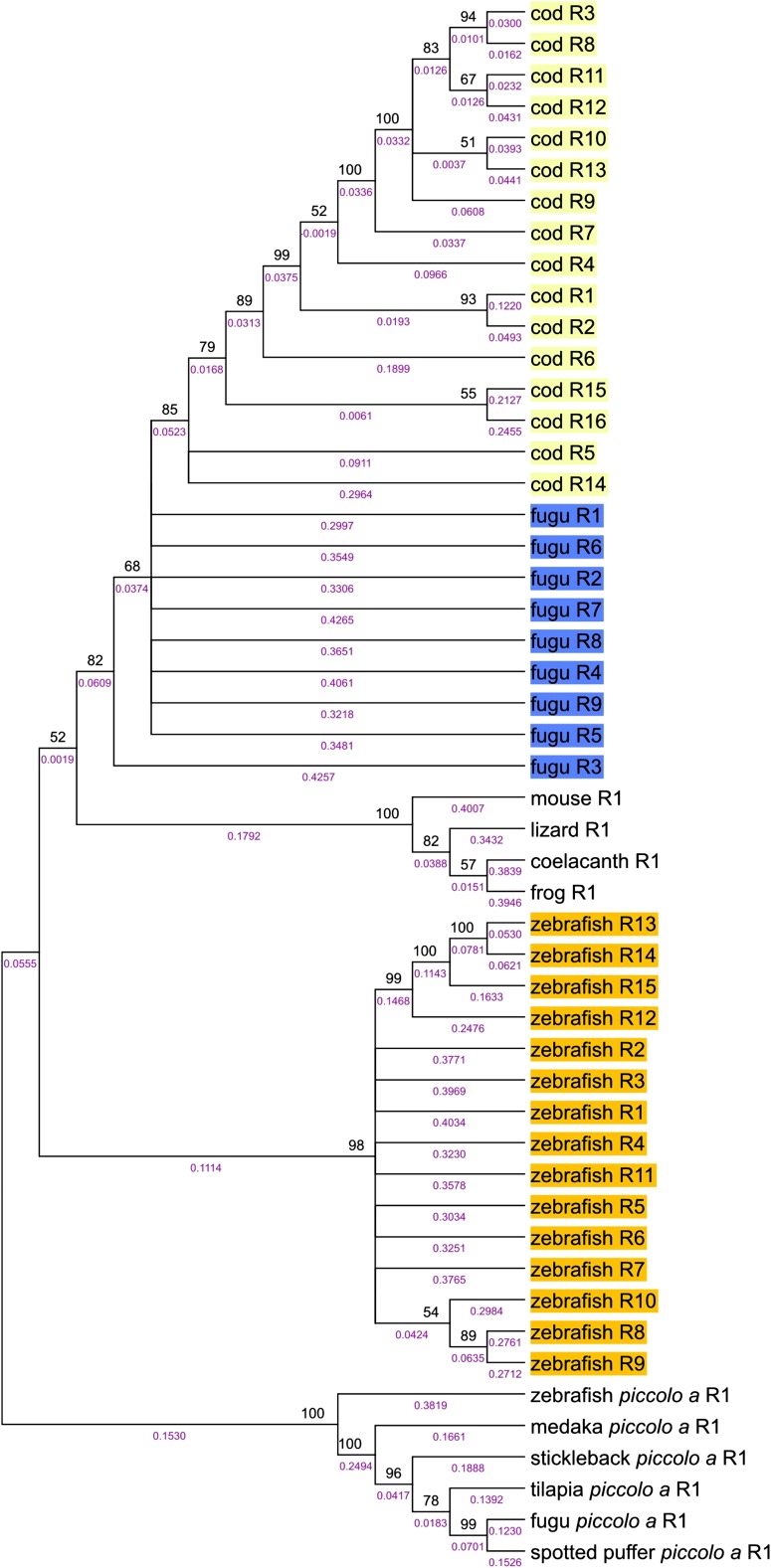
Evolutionary tree of repeated cod and zebrafish Piccolo gene exons encoding zinc finger domains. The evolutionary history of the repeat exons was inferred using the neighbor-joining method. Branches corresponding to partitions reproduced in less than 50% bootstrap replicates are collapsed. The percentages of replicate trees in which the associated sequences clustered together in the bootstrap test (1000 replicates) are shown next to the branches. Branch lengths are presented in purple and were computed using the maximum composite likelihood method and are in the units of number of base substitutions/site. The teleost repeats are color coded by species. All codon positions were included. All positions with less than 50% site coverage were eliminated from the analysis. There were a total of 861 positions in the final dataset. The alignment used to construct the tree is available in Figure S14.

Although the gene structure of medaka, fugu, spotted puffer, stickleback, and tilapia all are consistent with a simple amplification of the “central” exon during teleost evolution, the structure of zinc finger repeat exons in the cod and zebrafish *pclob* genes suggest the situation may be much more complicated. Using similar methods as outlined previously, I aligned the repeated zinc finger exons from cod, zebrafish, and fugu as a representative of the more closely related percomorph species. Surprisingly, all of the 16 cod zinc finger exons clustered in a single branch (Figure 7). Further examination of the cod zinc finger exons revealed they are much less divergent from each other than repeats of other species. Specifically, the mean divergence of cod repeats was 0.268 base substitutions/site, whereas the mean distance among repeats of the other teleosts ranged from 0.676 base substitutions/site for tilapia to 0.839 for zebrafish, suggesting that the cod repeats diverged much more recently than those of other teleosts. The analysis of cod *pclob* central repeat exons suggests that they have expanded more recently than the divergence of cod and the percomorpha lineage.

In contrast with the high similarity of the cod repeats, all of the zebrafish zinc finger exons were more divergent and clustered in a separate branch outside the other teleosts (Figure 7). Because these repeats have a relationship no more similar to the other teleost *pclob* repeats than to *pcloa* repeats, this finding suggests that these repeats amplified and diverged after the split of the percomorpha lineage from zebrafish. These data suggest the possibility that the zinc finger domain amplification may have occurred independently multiple times in the evolutionary history of teleosts.

To determine whether the same ancestral gene contains the amplification of the zinc finger domain in all teleosts, I looked at the syntenic relations for the *piccolo* genes (Figure S10). In each teleost I examined, on the promoter side of each of the two zinc finger *pcloa* homolog were located a single semaphorin (*SEMA3A*) gene and a tetraspanin gene (*TSPAN11*). By contrast, in each species three distinct semaphorin homologs (*SEMA3D*, *SEMA3A*, and *SEMA3E*) and a metabotopic gluatmate receptor gene (*GRM3*) were located promoter proximal to the multi zinc finger *pclob* homolog (except for cod where the contig ended in the last *SEMA3* gene). Thus, I conclude that the same ancestral ortholog has expanded in cod, zebrafish and the percomorphs.

### Novel IZF domain defined by repeated exons

To determine how the central zinc finger exons may have amplified, I examined their structure in more detail. First, I examined the alignments of the repeats from all species and identified a previously unrecognized conserved motif in the Piccolo and Bassoon family. This motif was not previously defined as a unique domain because it occurs only in one copy (between zinc finger 1 and 2) in currently characterized and annotated Piccolo and Bassoon genes, and thus was simply viewed as part of the general conservation between Piccolo and Bassoon genes. In the central region between the CXXC motif encoded in the 5′ of the exon and the three CXXC motifs encoded in the 3′ of the exon is a highly conserved serine- and threonine-rich 22 aa sequence of consensus: SVTGKMFGFGSSIFSSASTLIT. BLAST analysis of the consensus motif did not identify any similar motif in other proteins. This core motif was highly conserved among all repeats of all the *pclob* genes of teleosts I examined (except tilapia repeat 8) as well as nonteleost Piccolo genes (Figure S11). A more extended consensus of domain of 62 aa was present in the majority of the repeats, although there was substantial variation in this more extended domain including insertions and deletions within the extended region (Figure S11). Surprisingly, in teleost *pcloa* genes the core domain is interrupted with sequences that appear to represent a duplication of sequences present in the adjacent extended domain (Figure S12A). The core domain was also largely conserved in the Bassoon genes (Figure S12B). I propose that this motif be named the IZF motif. I speculate on the potential function of the motif in the discussion.

### Divergence by insertions and deletions between the IZF and zinc finger domains

**Figure 8:**
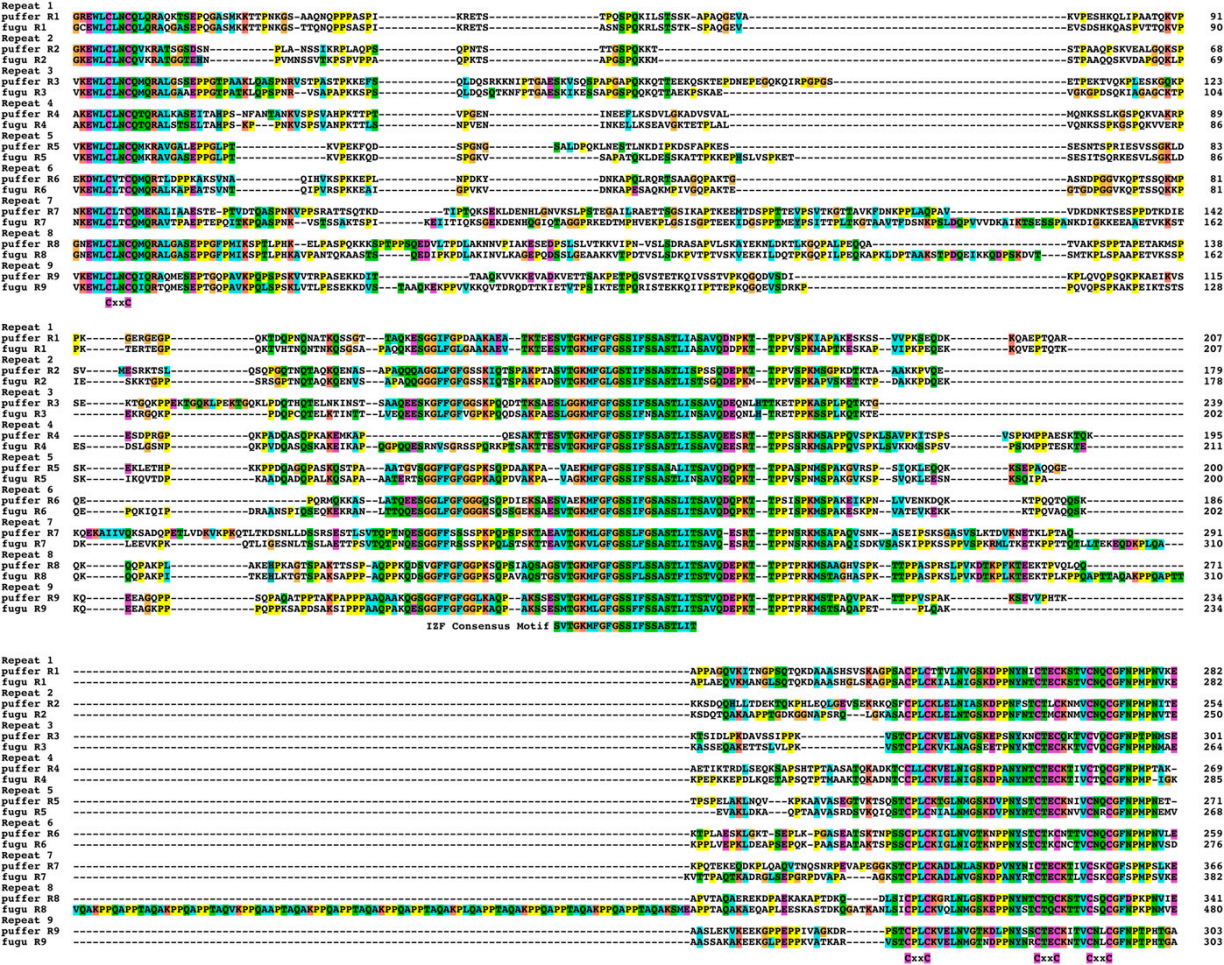
Alignment of green spotted puffer and fugu repeated zinc finger domains. An alignment of the translation of green spotted puffer and fugu zinc finger domain repeating exons. The position of the IZF consensus motif and the four CxxC motifs are labeled below the alignment. The sequences were aligned using MUSCLE and formatting of the alignment was performed using Clustal X as described in materials and methods.

Analysis of the sequence of repeats revealed that a major driving force for divergence of repeats is insertion and deletion of sequences between the N-terminal CXXC motif and the IZF and between the IZF and the three CXXC motifs. To document this, I first examined the alignment of spotted puffer and fugu, which are estimated to have diverged ∼50 million years ago (Steinke *et al.* 2006). Both of these species have retained the ancestral 9 repeat organization I proposed previously. A detailed look at the alignment between these repeats illustrates that a few repeats contain primarily nucleotide substitutions and only a minor contribution from insertions and deletions (Figure 8). For example, fugu-puffer repeat 1 divergence consists of 57 nonsynonymous substitutions, and 2 one aa insertions (one in each species leaving the repeat length identical). However, the differences between most pairs of repeats consisted of multiple insertions and deletions in addition to extensive non-synonymous substitutions. These range from small 1 to 4 aa insertions and deletions, to larger repeated insertions. For example, repeat 8 from fugu contains an insertion of 10 tandem repeats of PPQAPPTAQAK (or close variations thereof) between the IZF and the three CXXC motifs.

The comparison of puffer and fugu repeats also clearly illustrates that divergence between different repeats has occurred extensively via the insertions and deletions specific to individual repeat modules. For example, repeats 2, 5, and 6 all show distinct deletions of sequence just C-terminal to highly conserved CXXC motif. Because these deletions are present in both fugu and spotted puffer, they represent relatively old events that occurred between the time of the divergence puffers from the percomorphs (analyzed herein) and the divergence of spotted puffer from fugu. Analysis of the more distantly related stickleback, tilapia, and medaka, which also retain strong evidence of an ancestral 9 repeat organization, shows substantially more insertion and deletions found on both sides of the central IZF motif, thus shifting the spacing between the zinc finger and the IZF domain (Figure S12). The change in size of repeat exons was much more extensive than the change in size in the other conserved exons of the *pclob* genes. Specifically, of the last 18 exons of *pclob* (which encode the terminal PDZ C2A and C2B domains), 11 are identical in size in all the teleosts I examined, and 4 others are different by less than 3 aa in size (Table S1). In contrast, the zinc finger exons are largely divergent in size (Table S1 and Figure S13). Thus, this analysis suggests that the repeated zinc finger exons are under strong selective pressure to maintain a 5′ CXXC domain encoding the C-terminus of a zinc finger, a central domain encoding the IZF domain, and a 3′ domain coding for the three N-terminal CxxC portion of the zinc finger. In contrast, there appears to be little selective pressure to maintain the spacing between these domains.

## Discussion

Proteins containing domain repeats are relatively common in multicellular organisms ranging from approximately 9 to 17% in species that have been examined and account for almost 50% of all domains found in vertebrate proteins (Bjorklund *et al.* 2006). Previous analysis of the structure of multidomain proteins has provided some insight about the mechanisms leading to repeat expansion (and loss) in proteins (*e.g.*, Liu *et al.* 2005; Bjorklund *et al.* 2006, 2010), but our understanding of the mechanisms driving such domain expansion during evolution remains limited. Here I describe and analyze a case in which the zinc finger domain present in the neuronal protein Piccolo has greatly expanded selectively in the teleost lineage after the WGD that occurred shortly after the divergence of teleosts from the terrestrial vertebrate lineage (Amores *et al.* 1998; Taylor *et al.* 2003; Jaillon *et al.* 2004; Kasahara *et al.* 2007). The analyses reveal that the zinc finger domain has expanded in only one of the two teleost homologs. Furthermore, the amplification occurred at least in part by via duplication of single exons, and evidence suggests that expansion of this domain has occurred multiple independent times in the teleost lineage.

A WGD is thought to have occurred after divergence of the teleostei from chrondrostei (paddlefish and sturgeons) and holostei (gars and bowfins) approximately 320−370 million years ago (Kasahara *et al.* 2007; Amores *et al.* 2011). Analysis of genome wide gene structure in sequenced teleosts suggests that rediploidization after the WGD occurred rapidly with the loss of one of the duplicated copies in ∼75 to 85% of genes (Jaillon *et al.* 2004; Woods *et al.* 2005; Brunet *et al.* 2006). Most current models of rediploidization (Force *et al.* 1999) postulate that duplicate genes have been retained either due to gene neofunctionalization (where one gene acquires a new function that is selected for) or gene subfunctionalization (where each paralog loses reciprocally a component of the ancestral function, for example, reciprocal loss of regulatory elements or protein expression domains). The similarity and broad expression of the *piccolo* genes and the unique structure of *pclob* argue for neofunctionalization being the mechanism at work for the maintenance of two Piccolo homologs in teleosts, though functional analysis of mutants in the genes will be required to make a definitive conclusion on the matter.

### Function of the zinc finger domain repeats

The zinc finger domain of Piccolo belongs to a small family of zinc fingers that coordinate two zinc ions using a series of 4 pairs of cysteines found in Bassoon, Piccolo, RIM, and Rabphilin 3A (Fenster *et al.* 2000). In the case of RIM and Rabphilin, the zinc finger domain has been demonstrated to interact small synaptic vesicle membrane-associated GTPases (Rab3 and Rab27) via the zinc finger region (Wang *et al.* 2001). The zinc finger itself does not interact with the Rabs, but the zinc finger is required for the interaction. Structural analyses of both the RIM and Rabphilin zinc finger interacting with Rab3 revealed that alpha helices both N-terminal and C-terminal of the zinc finger are coordinated by the zinc finger (Ostermeier and Brunger 1999; Dulubova *et al.* 2005). In closely related FYVE zinc fingers, the domain binds directly to membranes. Specifically, the fingers coordinate basic residues and histidine that mediate binding to phosphatidylinositol-3-phosphate at the membrane surface (Misra and Hurley 1999; Stenmark *et al.* 2002). Experimental evidence suggests that the zinc finger domains of Piccolo and Bassoon likely interact with synaptic vesicles. Immunoelectron microscopy has shown that Bassoon can associate with vesicles (Sanmarti-Vila *et al.* 2000). A green fluorescent protein fusion to the zinc finger domains of Bassoon colocalizes with synaptic vesicles whereas a fusion to the whole protein is more restricted specifically to the cytomatrix of the active zone of the synapse (Dresbach *et al.* 2003). The molecular mechanism by which an interaction with vesicles might be mediated is currently not clear. The Bassoon zinc finger region binds to pra1 (Fenster *et al.* 2000), a protein which binds to prenylated Rab proteins and the synaptic vesicle protein synpatovrevin/VAMP (Martincic *et al.* 1997). The identification of the highly conserved IZF domain suggests that it could be a key player in the functional interactions mediated by the zinc finger /IZF repeats. That the IZF domain is exceedingly rich in serine and threonine would make it an excellent target for phosphorylation that could mediate reversible interaction with synaptic vesicles either directly or indirectly. Biochemical studies using the IZF domain may provide a new approach to defining the molecular mechanism by which the zinc finger act in Piccolo vesicle clustering function. In addition, the molecular, genetic, and physiological tools available for the study of zebrafish and medaka provide promising avenues for defining the role of the zinc finger domain in Piccolo protein in neuronal function.

### Aligning repeated exons with diverse lengths

Performing phylogenetic analysis on genes that contain significant variation in sequence due to insertions and deletions is very challenging. The relationships deduced from trees constructed using sequence data are highly dependent on the quality of the alignment (Thompson *et al.* 1999). Aligning sequences with significant insertions and deletions is complicated because weighting the choice between maintaining an ungapped but nonhomologous alignment *vs.* positioning and sizing of gaps is difficult (Cartwright 2009). In addition, phylogenic trees usually are constructed after removing regions of the alignment containing gaps, thus the number and position of gaps inserted into a sequence can greatly influence the resulting phylogeny. Although some methodologies that use gap information have been created and evidence indicates that significant information is present in gaps (Loytynoja and Goldman 2008; Rivas and Eddy 2008; Dwivedi and Gadagkar 2009; Dessimoz and Gil 2010), use of these approaches is not well established in the literature, and thus I did not attempt to use these methods to align the zinc finger repeats. In the case of the zinc finger repeat exons, removing all sequences that are not conserved in all repeats leaves insufficient data to construct statistically significant trees as the divergence in size of repeats is extensive (from 207 to 618 aa in length). I therefore opted to use all columns that were ungapped in at least 50% of the sequences. Furthermore, I modified the gap opening and extension parameter of MUSCLE to limit the number of gaps introduced in the alignment. Because these decisions are to some extent arbitrary, I also aligned the sequences using Clustal X under default parameters and MUSCLE under default parameters and constructed trees for all 3 alignments (Figure S15 for trees, and Figure S14, Figure S16, and Figure S17 for alignments). Although differences are apparent in the trees obtained using the different alignments, the fundamental conclusions made about the distinct clustering of cod, zebrafish and percomorph repeats are the same regardless of alignment parameters. Thus, although the complexity of aligning repeats of different lengths likely has introduced some uncertainty in the precise trees I obtained, they are unlikely to have misled us about the general evolutionary principles that govern the origins of the repeat exons. The subsequent discussion assumes the phylogenetic analysis is basically sound.

### Model of amplification and deletion

Analysis of the repeats from percomorphs and zebrafish indicate that repeats have been added and lost from *pclob* in discrete events that inserted or deleted single exons. In the case of percomorphs, fugu, and spotted puffer repeat organizations are representative of a proposed ancestral state of *pclob* zinc finger exons in the percomorph lineage. Insertions and deletions of single repeat exons have occurred in medaka, stickleback and tilapia (Figure 6). Similarly, the phylogenetic tree of zebrafish repeats is consistent with repeats 12-15 having appeared through individual single exon duplication events. Multiple methods have been proposed to account for repeat amplification and contraction including strand slippage (Petruska *et al.* 1998), retrotransposition (Xiao *et al.* 2008), gene conversion (Chen *et al.* 2007), and illegitimate recombination (either nonhomologous or unequal crossover mediated) during meiotic recombination (Van Rijk and Bloemendal 2003). Strand-slippage is primarily associated with trinucleotide repeat amplification and is thus unlikely to be relevant to Piccolo. Furthermore, the amplified repeat domains are also not associated with putative transposon sequences. Finally, although I cannot formally eliminate gene conversion as a plausible mechanism for the exon amplifications I describe, gene conversion tracts tend to be smaller [usually less than 300 bp (Chen *et al.* 2007)] than the zinc finger exons repeated in *pclob*. Thus, I propose a model based on unequal crossovers between repeats. Specifically, I propose that unequal crossovers between homologous 5′ CxxC, central IZF, or 3′ three CxxC encoding regions of adjacent exons (and more rarely between more distantly spaced exons) can account for the changes in repeat spacing in the percomorphs and for the expansion of repeat exons 12−15 in zebrafish. Although larger numbers of similar events also could explain the amplification from 1 to 9 central repeat exons that occurred prior to the diverge of the percomorph species I examined, analysis of the phylogenetic trees do not yield a simple set of events that can account for the observed repeat order and number.

### Zebrafish and cod *pclob*: evidence for independent amplification of zinc finger repeats in teleosts

The analysis of *pclob* suggests that the zinc finger exon structure of fugu and spotted puffer represent an ancestral state present before divergence of medaka and the puffers ∼190 million years ago. Although cod diverged from the percomorphs before this time (Setiamarga *et al.* 2009), the zinc finger exons from cod are both much more numerous and much less divergent than in the percomorphs. However, the cod repeats do fall within the percomorpha repeat clade, suggesting some relationship between the two. At least three possible scenarios could account for this difference. First, the repeats in the cod lineage were lost by unequal crossover and then more recently reamplified from a single or a few repeat exons. Second, the repeat number in the cod lineage could have amplified greatly (likely to more than 24 repeats) with much of this amplification occurring via recurrent amplification of a small subset of repeats. Subsequently, the repeats more closely related to the percomorphs were lost by unequal crossover, leaving the current 16 repeats. Third, it is possible that multiple gene conversion events harmonized the repeat structure after cod repeats amplification from the ancestral 9 repeat state. I favor the first scenario as it requires the least additional events (one unequal crossover event which deletes 8 repeats). In the case of zebrafish, the repeats show no significant relation to the percomorpha repeats but unlike the cod repeats are very diverse in sequence (except for repeats 12−15). The simplest explanation for this divergence is that the amplification of *pclob* zinc fingers was initiated independently after the divergence of zebrafish and the euteleostei (cod and percomorphs ∼280 Mya). Thus, I propose that amplification of repeats in *pclob* has occurred independently at least 3 times in the teleost lineage.

### Frequency of duplication and amplification events

The analysis of the percomorph species that diverged from ∼50 million years ago (green spotted puffer and fugu) to 175 million years ago (stickleback and medaka) indicate that loss or insertion of a whole repeat exon is a very rare event, occurring only 2 and 3 times, respectively, since the divergence of the five percomorphs I examined. In contrast, the repeats in cod appear more closely related, suggesting that repeat expansion may also occur more frequently than every 50−100 million years. In addition, I also found that zebrafish repeats 12 through 15 are closely related, suggesting they amplified more recently. Why this dichotomy in stability of repeats? I propose that the exon repeats are maintained via two different mechanisms of selection. The universal emergence of duplicated zinc finger repeats in teleosts suggests strong selection pressure to amplify the zinc finger repeats. However, repeated homologous sequences are unstable because they promote unequal crossovers during meiotic recombination. I propose that three distinct regions in each exon are under negative selective pressure (for conservation): the zinc finger domain CxxC motifs at both edges of the exon and the IZF motif in the middle of the exon. This results in three short regions in each exon that maintain high homology between the repeated exons and thus maintain the potential for unequal crossover events that mediate repeat expansion or contraction. In addition, positive selection for rapid divergence of the sequences between these conserved regions within the repeated exons drives divergence of the repeats thus reducing the extent of repeat-repeat homology and stabilizes amplified repeat exons. Such a model would predict a scenario in which after an amplification, the event repeat number would be unstable and then over time, as positive selection drives divergence of the repeats, amplification and deletion of repeats would become progressively less frequent. The cod repeat arrangement represents an earlier state in this process of repeat stabilization and the percomorphs a more mature state of the process.

### No other examples of drastic expansion of a single of two homologs in teleosts or other species

Gene amplification in the teleost lineage has been extensively studied and numerous examples of selective gene amplification in teleosts have been described. For example, the MHC I family has been greatly amplified in cod (Star *et al.* 2011) and the TRIM gene family has differentially amplified in different teleost species (van der Aa *et al.* 2009). In addition, there are numerous examples of domain amplification in the vertebrate lineage, including well-studied examples such as nebulin in which a super repeat of 7 actin binding domains has been amplified multiple times in many different vertebrate lineages (Bjorklund *et al.* 2010). However, I am not aware of another example of extensive amplification of a domain specifically in one of two ohnologs in the teleosts. The amplification of a whole gene is under different constraints from amplification of a domain because during amplification of a domain like the zinc finger domain of *pclob*, there is continued selection for function of each domain. By contrast, during amplification of whole genes such as the TRIM and MHC I families each duplicated gene presumably can be selected on independently of the other amplified genes.

To assess how unusual the case of *pclob* zinc finger domain amplification is, I used Ensembl Bio-Mart to identify all “genes” (as defined by Ensembl gene models) duplicated in zebrafish that have only a single mouse homolog. I then asked among these gene duplications that had been maintained in zebrafish, how many of these zebrafish gene pair contained different numbers of total identified domains from their mouse homolog. I analyzed 1598 genes with two co-orthologs in zebrafish, but only one in mouse. Among these, in 791 cases both zebrafish genes had the identical total number of PFAM domains as the mouse gene. Among the remaining trios, only 371 had one zebrafish ortholog with an identical domain count as the mouse gene, and the other zebrafish gene with a distinct domain count. Among these only 132 had an increased domain count compared with the mouse gene, and only four genes had an increase of three or more domains compared with the mouse homolog. The sole case with a four-domain increase was the increase in C2 domains in *esyt1b* to 9 from the 5 found in the mouse *esyt1* and zebrafish *esyt1a*. This increase has been previously documented and is present in all teleosts examined and consists of a internal duplication which likely occurred early in the teleost lineage (Craxton 2010). Two of the three other cases were the result of misannotation (*PEAR1* and *SPEG*), and the last case (*SHARPIN*), one of the zebrafish genes, has added three new domains.

Poor annotation of the zebrafish genome constrained the stringency of the analysis. For example, I did not detect *pclob* because this gene has not been properly annotated, likely due to the duplications I describe herein. Among the 371 aforementioned cases, many differences between one ortholog in zebrafish and the mouse gene were loss of domains, and it is likely that many of these cases are the result of poor annotation (which should improve greatly as RNAseq data are incorporated into gene structure annotation). In summary, although the bioinformatics analysis was constrained by data quality, the case of *pclob* zinc finger domain amplification appears to be very rare among all characterized zebrafish genes. Thus, it likely represents an unusually prolific case that may be particularly useful in defining mechanisms that are capable of driving domain amplification and contraction during evolution.

### Evolution of *pclob*

Although I have examined and documented the expansion of the number of zinc finger domains in *pclob* in the teleosts, it remains unclear whether duplication of Piccolo provided a unique evolutionary advantage to teleosts or if it might generally provide advantages to vertebrates. *Xenopus laevis* is pseudo-tetraploid due to a recent WGD [20−55 Mya (Chain and Evans 2006; Semon and Wolfe 2008]), is extensively studied experimentally, and is likely to be completely sequenced in the near future. Several species of South American rats are also thought to have undergone recent WGD, although the timing of this event has not been defined (Gallardo *et al.* 2006). It will be interesting to explore whether recent tetraploidization events also have driven the expansion of Piccolo in amphibians and terrestrial vertebrates.

The teleost lineage should also prove intriguing for further study of the *pclob* family. An additional WGD has also occurred in several teleost lineages: notably in carps and salmonids (Leong *et al.* 2010; Wang *et al.* 2012). Has further diversification of the zinc finger repeat number in *piccolo* genes accompanied these amplifications of the Piccolo family? Unfortunately, current transcriptome datasets from these fish are not of sufficient depth and quality to assembly the exceedingly large *bassoon* and *piccolo* genes, but RNAseq analysis should eventually address the issue. In addition, the large diversity of teleost species including radiation of African cichlid species in the rift lake Valley (Seehausen 2006) should provide a unique experimental laboratory to examine the molecular mechanisms driving the evolution of domain repeats in detail unthinkable even a few years ago.

## Supplementary Material

Supporting Information
