# Supplementary material for: A Window into Domain Amplification Through Piccolo in Teleost Fish
Source: G3 (Bethesda). 2012 Nov 1;2(11):1325–39. doi: 10.1534/g3.112.003624 (PMC3484663; doi:10.1534/g3.112.003624)
Supplement: Supporting Information [file supp_2_11_1325__index.html]

Supporting Information 

# A Window into Domain Amplification Through Piccolo in Teleost Fish

## Supporting Information for Nonet, 2012

**Files in this Data Supplement:**

- Supporting Information - Figures S1-S17 and Tables S1-S3 (PDF, 1.7 MB)
- Figure S1 - Correlation in size of analogous introns in mouse and teleost Piccolo genes (PDF, 256 KB)
- Figure S2 - RT-PCR analysis of splicing patterns in the repeated zebrafish zinc finger exons (PDF, 2 MB)
- Figure S3 - RNAseq DNA of zebrafish *pclob* repeated zinc finger exons (PDF, 955 MB)
- Figure S4 - Zebrafish *piccolo* and *bassoon* expression as determined by *in situ* hybridization of adult brain (PDF, 3.6 MB)
- Figure S5 - Evolutionary Trees constructed from Teleost *piccolo* and *bassoon* genes (PDF, 584 KB)
- Figure S6 - Sequence alignments of *piccolo* genes (PDF, 1.5 MB)
- Figure S7 - Sequence alignments of *bassoon* genes (PDF, 1.5 MB)
- Figure S8 - Additional Evolutionary trees derived from *bassoon* and *piccolo* genes (PDF, 587 KB)
- Figure S9 - Intron-exon organization of the zinc finger exons from *piccolo* genes (PDF, 922 KB)
- Figure S10 - Synteny between the teleost *pcloa*, *pclob* and mouse Piccolo genes (PDF, 2.5 MB)
- Figure S11 - Sequence alignments of the IZF domain of *piccolo* genes (PDF, 8.8 MB)
- Figure S12 - Alignments of the Core IZF domain from teleost *piccolo* and *bassoon* genes (PDF, 4.5 MB)
- Figure S13 - Alignments of the zinc finger repeats from percomorph fish (PDF, 562 KB)
- Figure S14 - Alignments of zinc finger repeats of all teleosts examined (PDF, 993 KB)
- Figure S15 - Comparison of evolutionary trees of repeated zinc finger domains obtained using distinct alignment methods and gap penalties (PDF, 2.7 MB)
- Figure S16 - Alignment of Zinc finger Repeats using Clustal W (PDF, 1.1 MB)
- Figure S17 - Alignment of Zinc finger Repeats using MUSCLE (PDF, 1 MB)
- Table S3 - Oligonucleotides used in this study (PDF, 59 KB)
- Table S1 - Piccolo gene splicing (.xls, 230 KB)
- Table S2 - Bassoon gene splicing (.xls, 130 KB)
- File S1 - Genbank Files (.zip, 2.9 MB)
