# Supplementary material for: A Window into Domain Amplification Through Piccolo in Teleost Fish
Source: G3 (Bethesda). 2012 Nov 1;2(11):1325–39. doi: 10.1534/g3.112.003624 (PMC3484663; doi:10.1534/g3.112.003624)
Supplement: Supporting Information [file supp_2.11.1325_003624SI.pdf]

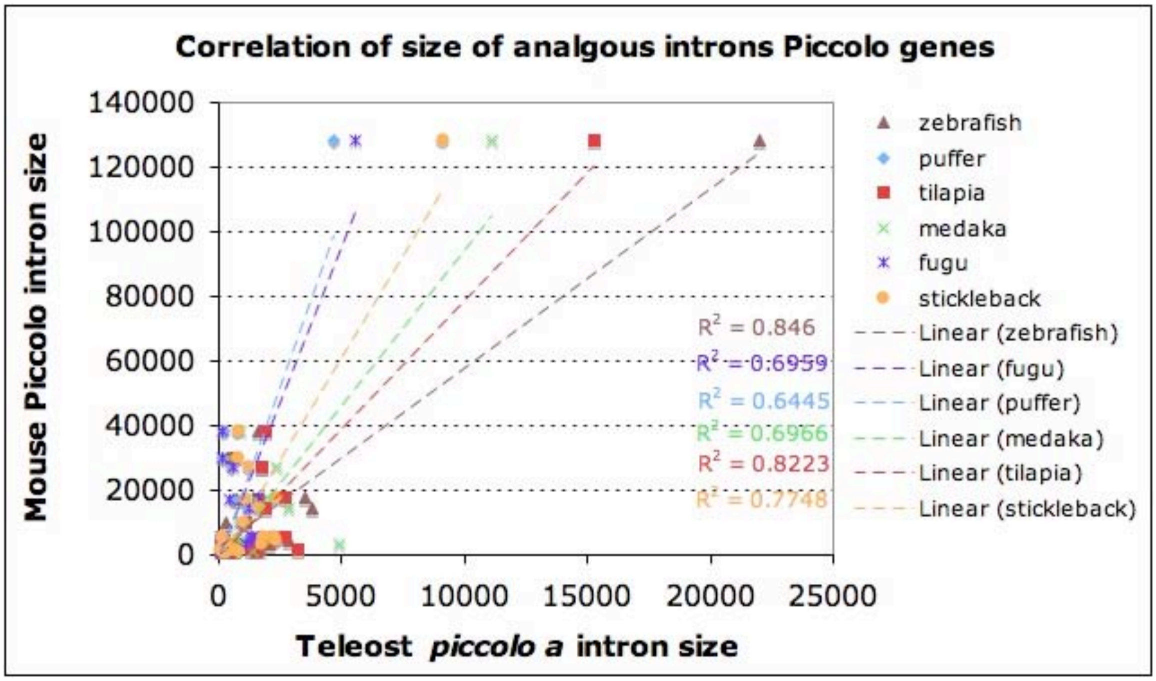

Figure S1 Correlation in size of analogous introns in mouse and teleost Piccolo genes. Mouse intron size was plotted against teleost intron size for the *pcloa* genes of zebrafish, green spotted puffer (listed as puffer in figure), fugu, medaka, tilapia and stickleback. A linear regression was performed and trend line and the correlation coefficient for each regression is presented.

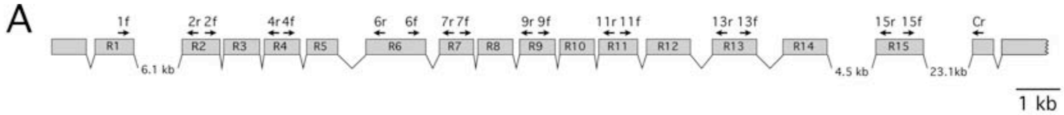

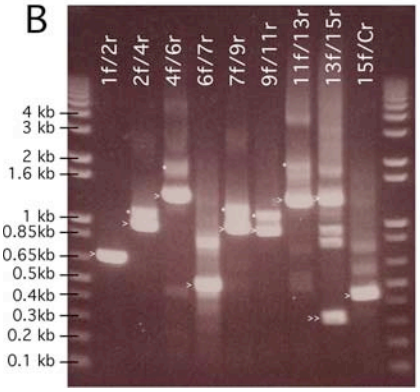

PCR primers	central exon junction tested	predicted size for cDNA (bp)	predicted size if exon skipped (bp)	expected genomic size (bp)
1f,2r	1|2	675	--	6797
2f,4r	2|3,3|4	977	166	1149
4f,6r	4|5,5|6	1384	571	2036
6f,7r	6|7	530	--	828
7f,9r	7|8,8|9	983	194	1218
9f,11r	9|10,10|11	960	176	1148
11f,13r	11|12,12|13	1309	319	1994
13f,15r	13|14,14|15	1326	330	4196
15f,Cr	15|CC	448	--	23640

Figure S2 RT-PCR analysis of splicing patterns in the repeated zebrafish zinc finger exons. A) Diagram of the exon structure of the repeated exons encoding zinc finger domains of zebrafish *pclob.* Name and position of oligonucleotide primers used for RT-PCR are also depicted above the representation of the genomic organization. The drawing is to scale except for the 3 large exons, the sizes of which are indicated in the drawing. A 1 kb scale bar is present on the right. B) Agarose gel separation of RT-PCR products obtained from amplification of first strand cDNA from 5-day-old zebrafish embryos. On the left is a table that tabulates predicted sizes of PCR products for genomic DNA, *pclob* cDNA, and cDNA size if an exon is omitted from the mature transcript. > mark DNA bands of the expected size if an exon is spliced as illustrated in A. * mark bands of the expected size of genomic DNA, >> mark the PCR products consistent with skipping of exon R14. The sizes of DNA markers are on the left.

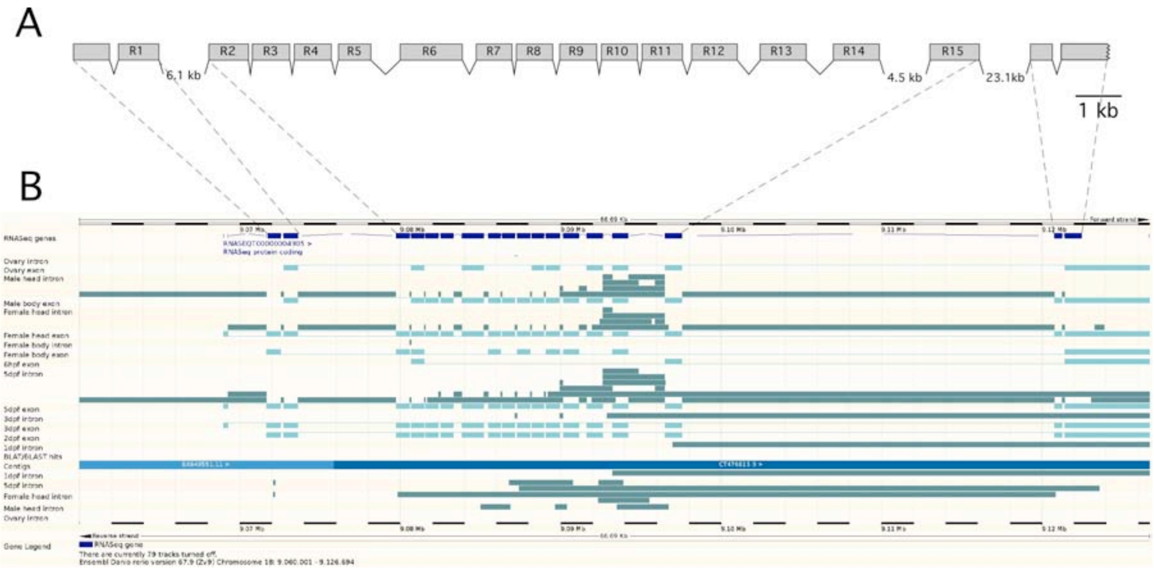

Figure S3 RNAseq DNA of zebrafish *pclob* repeated zinc finger exons. A) Diagram of the exon structure of the repeated exons encoding zinc finger domains of zebrafish *pclob*. The drawing is to scale except for the 3 large exons, the sizes of which are indicated in the drawing. A 1 kb scale bar is present on the right. B) A contig view obtained from Ensembl v67 of the genomic organization of the zinc finger exon region of *pclob* (chr18: 9,060,001- 9,126,694). RNAseq predicted gene structure for *pclob* (RNAseqT000004905) is identical to the manual annotation in the zinc finger repeat exon region. Note that translation of the T000004905 transcript ends in the Ensembl display because of an error in the genomic sequence (see materials and methods for details). The consensus exon structures determined from RNAseq of sample of various age and/or body section are shown. Also shown are the intron splicing patterns utilized in each sample based on RNAseq data crossing splice junctions.

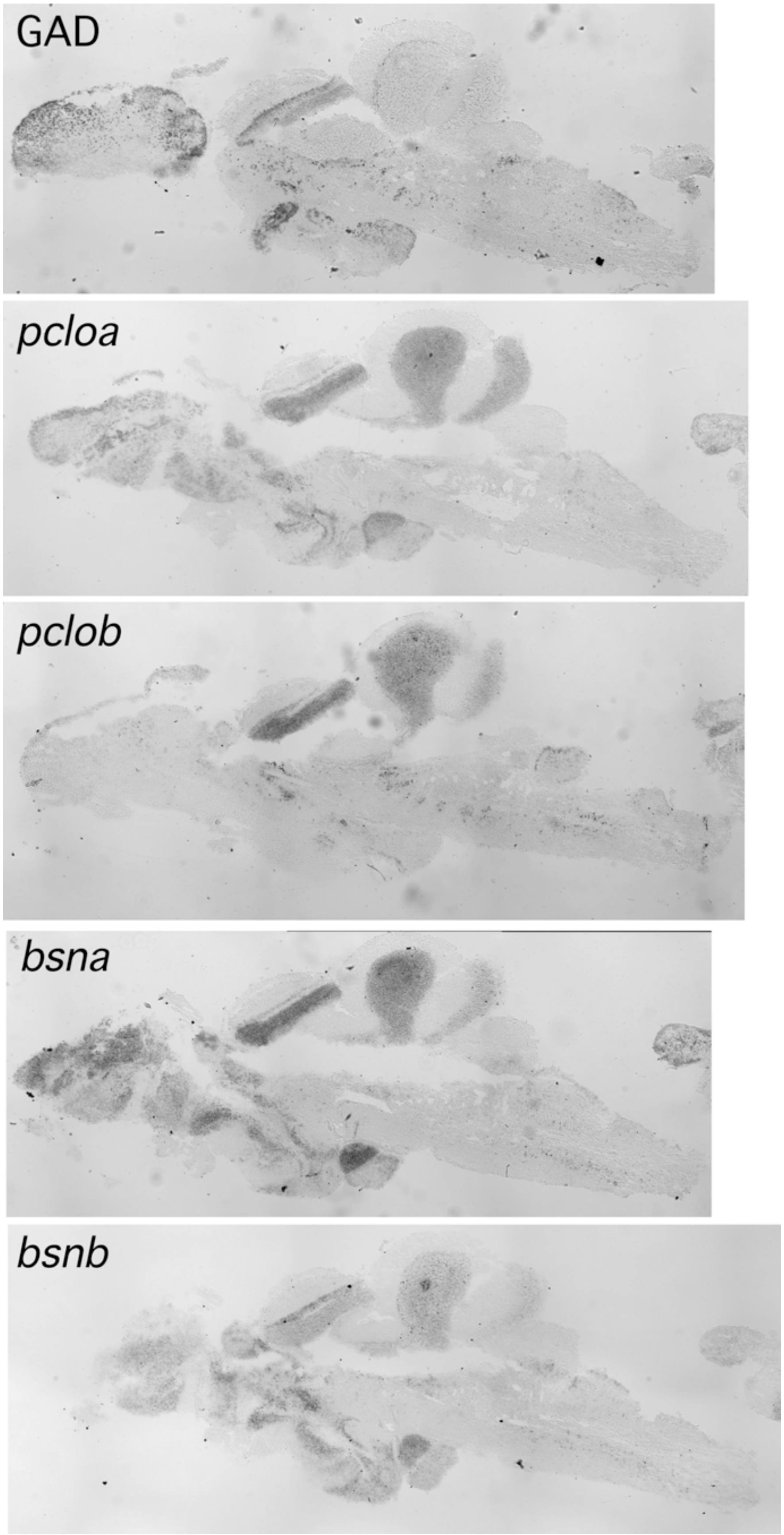
GAD
pcloa
pclob
bsna
bsnb

Figure S4 Zebrafish *piccolo* and *bassoon* expression as determined by in situ hybridization of adult brain. Sections of adult (~ 1 year old) adult wild type zebrafish brain were hybridized with DIG-labeled probes for zebrafish *pcloa*, *pclob*, *bsna*, *bsnb*, and zebrafish *gad1*. Hybridizing probes were detected using an alkaline phosphatase conjugated anti-DIG secondary antibodies, and alkaline phosphatase activity was detected using an NBT-BCIP colored precipitation reaction.

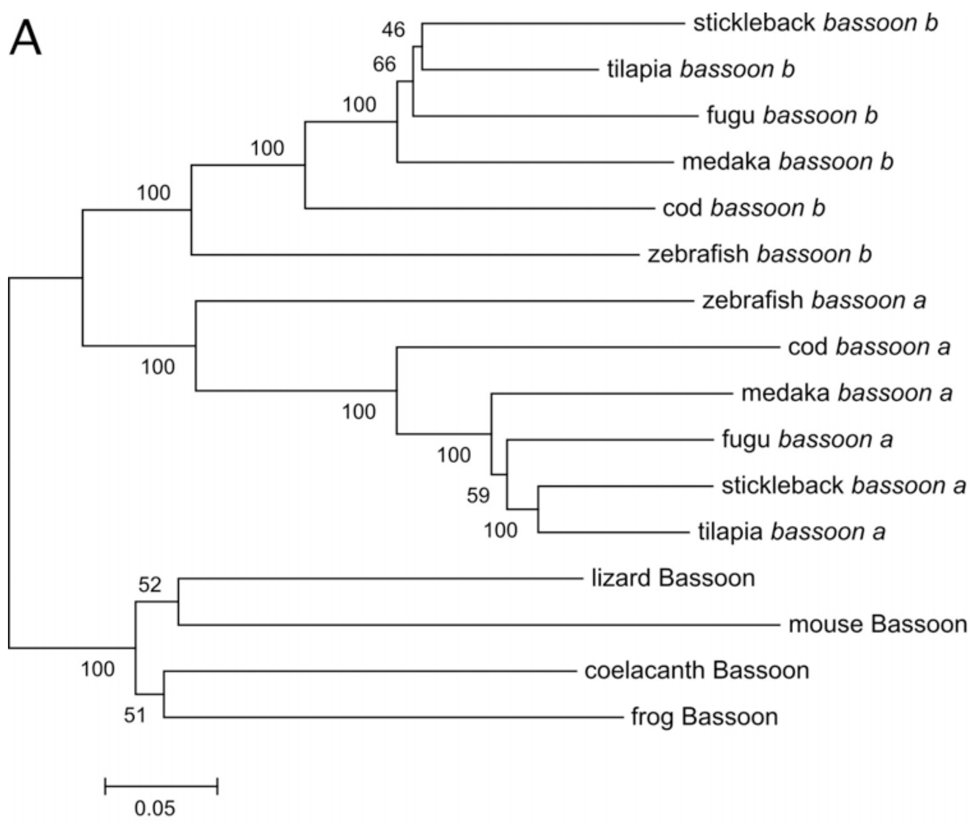
A
stickleback bassoon b
tilapia bassoon b
fugu bassoon b
medaka bassoon b
cod bassoon b
zebrafish bassoon b
zebrafish bassoon a
cod bassoon a
medaka bassoon a
fugu bassoon a
stickleback bassoon a
tilapia bassoon a
lizard Bassoon
mouse Bassoon
coelacanth Bassoon
frog Bassoon
46
66
100
100
100
100
100
100
59
100
52
100
51
0.05

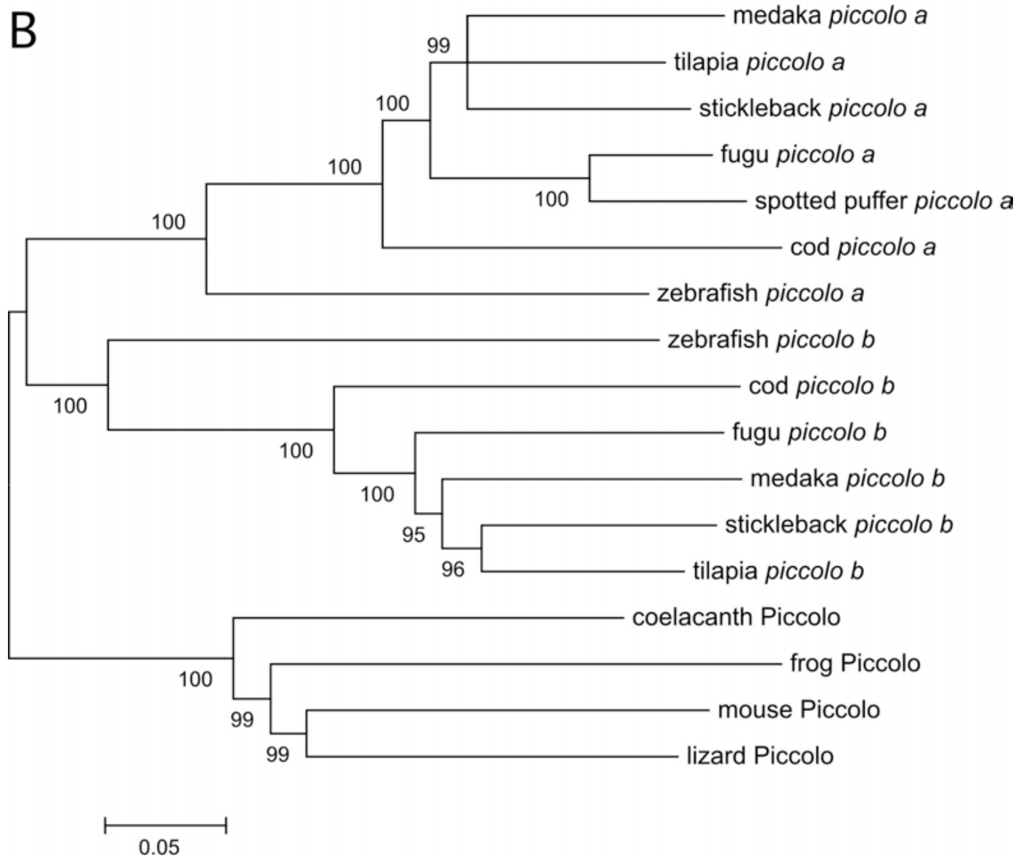
B
medaka piccolo a
tilapia piccolo a
stickleback piccolo a
fugu piccolo a
spotted puffer piccolo a
cod piccolo a
zebrafish piccolo a
zebrafish piccolo b
cod piccolo b
fugu piccolo b
medaka piccolo b
stickleback piccolo b
tilapia piccolo b
coelacanth Piccolo
frog Piccolo
mouse Piccolo
lizard Piccolo
99
100
100
100
100
100
100
100
95
96
100
99
99
0.05

Figure S5 Evolutionary Trees constructed from Teleost *piccolo* and *bassoon* genes. Evolutionary history was inferred from sequences using a neighbor-joining method. Numbers adjacent to the internal branches indicate bootstrap values. The trees are drawn to scale, with the branch length unit as base substitutions/site. Trees were constructed only from sequences C-terminal to the zinc finger motifs because of the diversity in zinc finger number in the *piccolo* homologs. A) Tree obtained using *bassoon* homologs using sequences from exon 5 onward. The tree is very similar to that obtained for complete *bassoon* (see Figure S7). B) Tree obtained using *piccolo* sequences (exon 5 onward for non-teleosts and teleost *pcloa* family members, exon 11 onward for stickleback *pclob*, exon 12 onward for fugu and tilapia *pclob*, exon 13 onward for medaka *pclob*, exon 18 onward for zebrafish *pclob*, and exon 19 onward for cod *pclob*).

Figures S6 and S7 are available for download at

http://www.g3journal.org/lookup/suppl/doi:10.1534/g3.112.003624/-/DC1.

Figure S6 Sequence alignments of *piccolo* genes. Piccolo sequence alignment used to create the trees in Figure S5 were aligned using MUSCLE and formatting of the alignment was performed using Clustal X as described in materials and methods. The bar graph below represents the level of conservation.

Figure S7 Sequence alignments of *bassoon* genes. Bassoon sequence alignment used to create trees in Figure S5 and S8 were aligned using MUSCLE and formatting of the alignment was performed using Clustal X as described in materials and methods. The bar graph below represents the level of conservation.

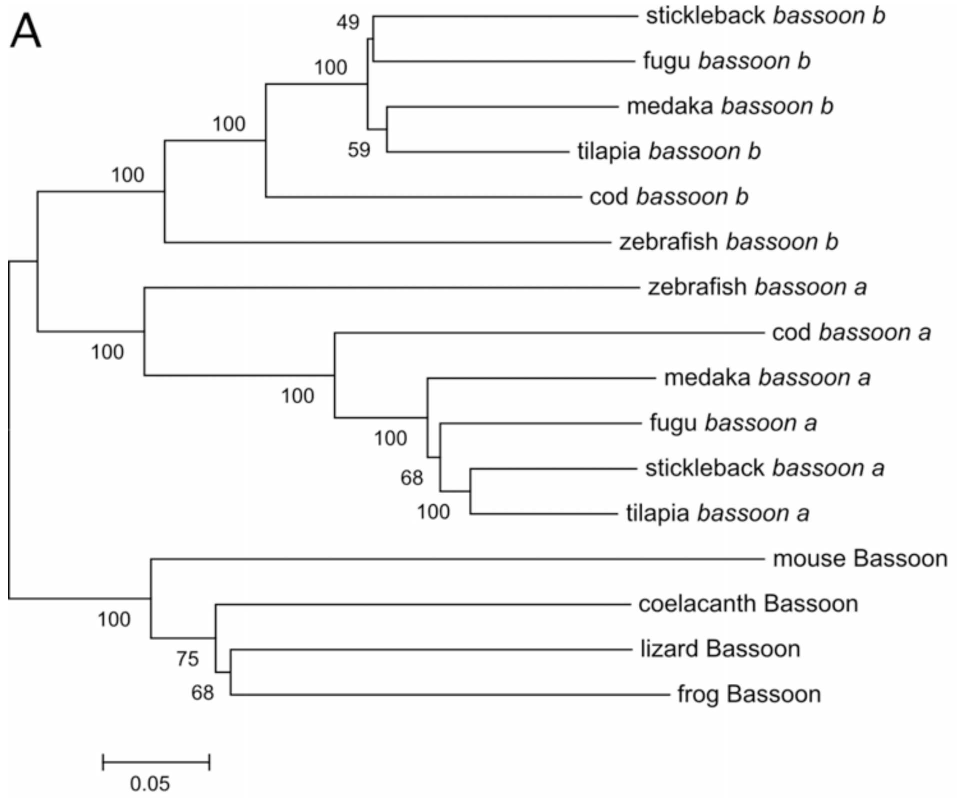
A
stickleback bassoon b
fugu bassoon b
medaka bassoon b
tilapia bassoon b
cod bassoon b
zebrafish bassoon b
zebrafish bassoon a
cod bassoon a
medaka bassoon a
fugu bassoon a
stickleback bassoon a
tilapia bassoon a
mouse Bassoon
coelacanth Bassoon
lizard Bassoon
frog Bassoon
0.05

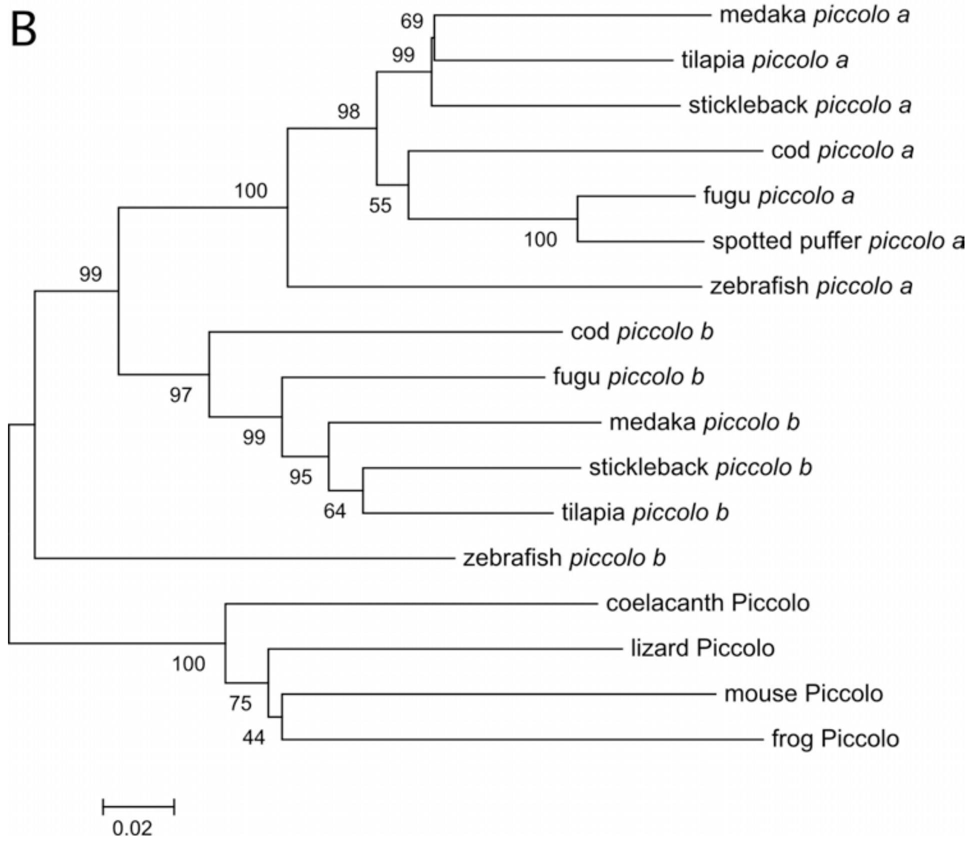
B
medaka piccolo a
tilapia piccolo a
stickleback piccolo a
cod piccolo a
fugu piccolo a
spotted puffer piccolo a
zebrafish piccolo a
cod piccolo b
fugu piccolo b
medaka piccolo b
stickleback piccolo b
tilapia piccolo b
zebrafish piccolo b
coelacanth Piccolo
lizard Piccolo
mouse Piccolo
frog Piccolo
0.02

Figure S8 Additional Evolutionary trees derived from *bassoon* and *piccolo* genes. Evolutionary history was inferred from sequences using a neighbor-joining method. Numbers adjacent to the internal branches indicate bootstrap values. The trees are drawn to scale, with the branch length unit as base substitutions/site. A) The *bassoon* tree was obtained from the entire Bassoon sequence including the zinc finger exons. B) The *piccolo* tree obtained including only the PDZ, C2A, and C2B domains that are not found in Bassoon proteins.

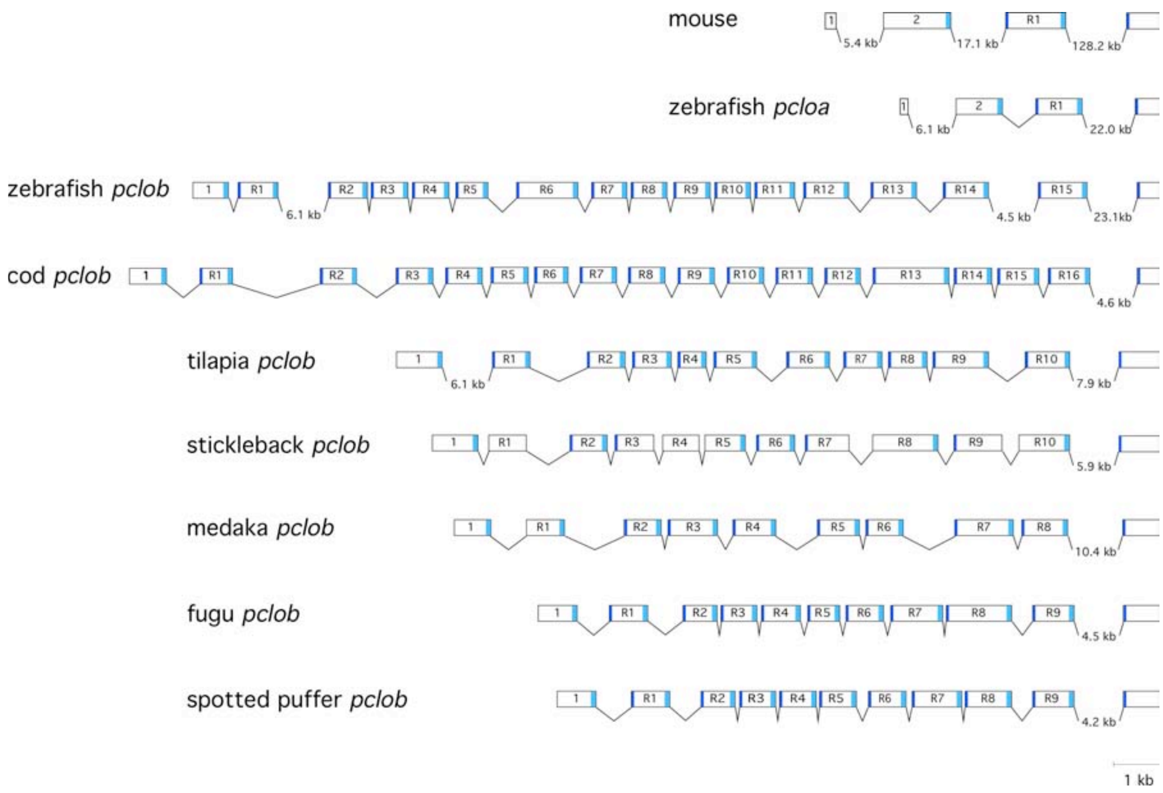

Figure S9 Intron-exon organization of the zinc finger exons from *piccolo* genes. Gene structure of the 5' portion of *piccolo* genes encoding the zinc finger repeats. Light blue and dark blue portions of exon mark the three CxxC and single CxxC components of the domain located on the '3 and 5' ends of the repeating exons. The genomic regions are shown to scale except for large introns, whose sizes are listed. Scale bar on bottom right is 1 kb.

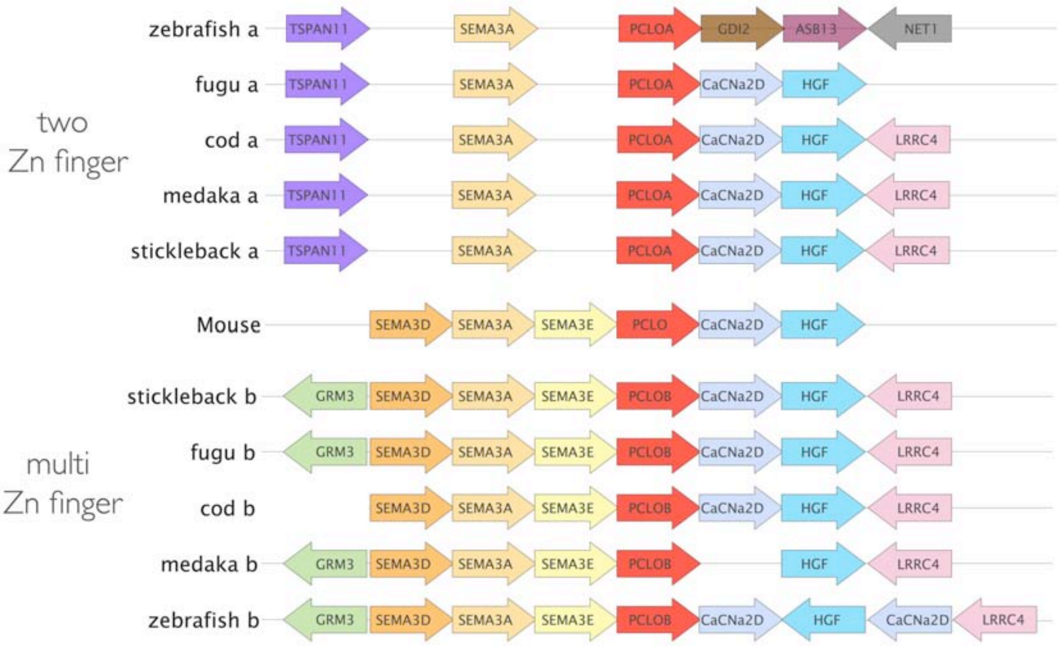

Figure S10 Synteny between the teleost *pcloa*, *pclob* and mouse Piccolo genes. Gene organization near *pcloa* and *pclob* homologs from teleosts. Genes are not shown to scale and certain small loci such as tRNA genes were omitted from the diagrams for simplicity.

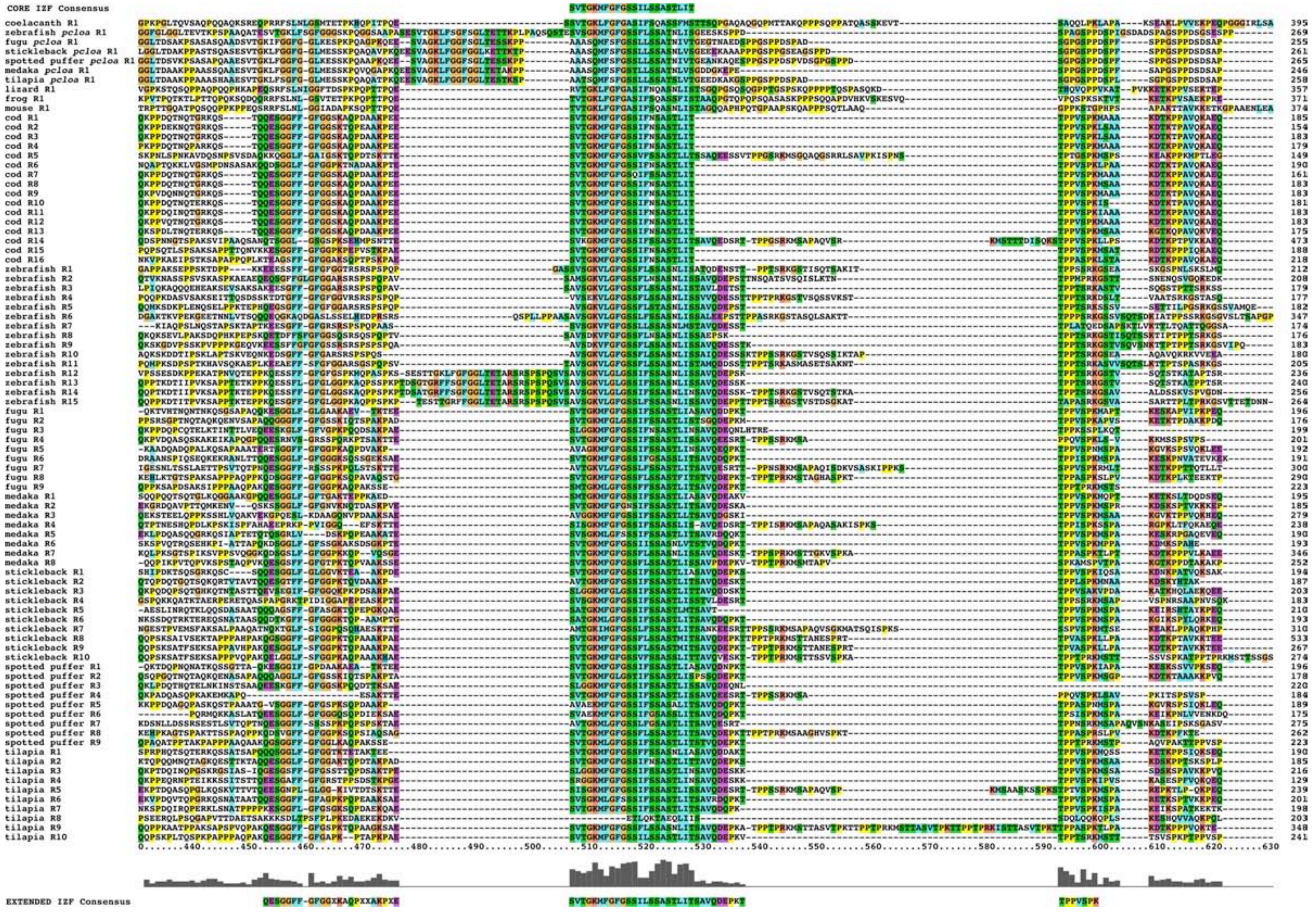

Figure S11 Sequence alignments of the IZF domain of *piccolo* genes. An alignment of central region of exons encoding the Inter Zinc Finger domain (IZF). *piccolo* repeat exon sequences were aligned using MUSCLE and formatting of the alignment figure was performed using Clustal X as described in methods. A bar graph below represents the level of conservation. A core IZF domain consensus sequence is positioned above the alignment and an extended IZF domain consensus sequence is positioned below the alignment.

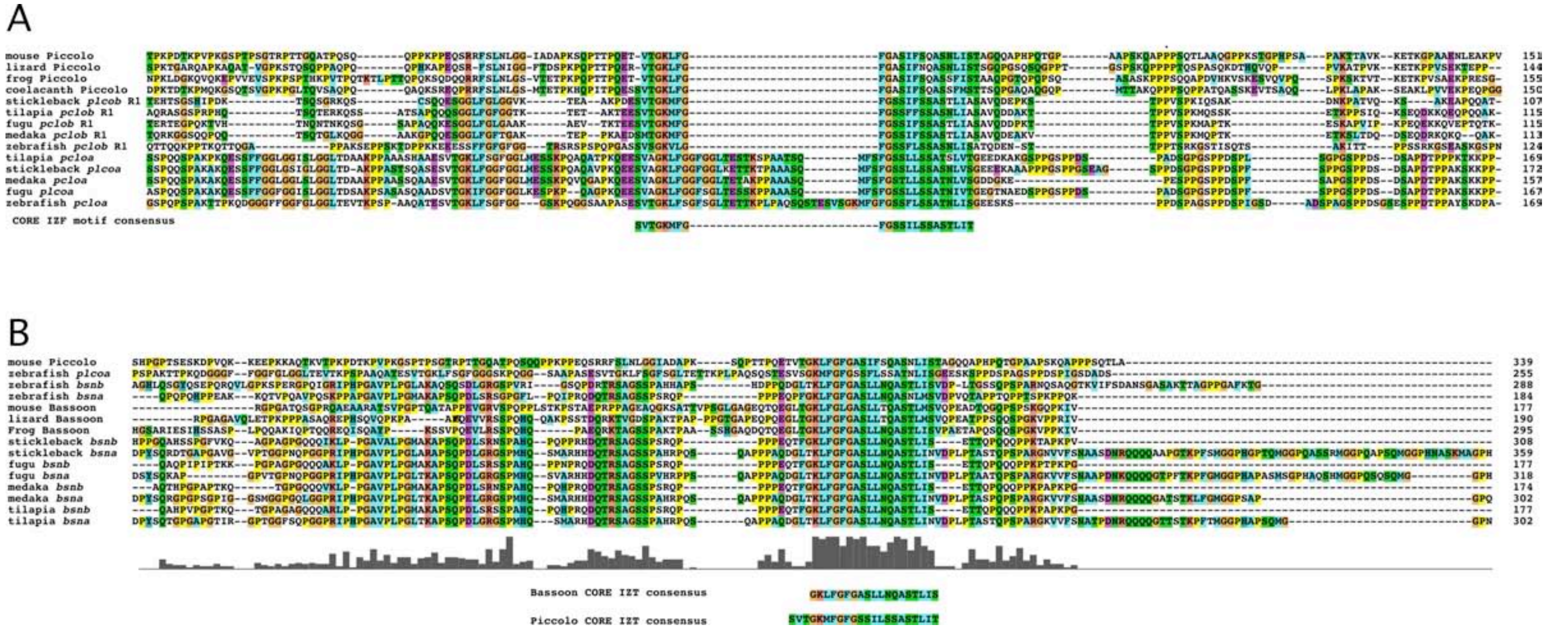

Figure S12 Alignments of the Core IZF domain from teleost *piccolo* and *bassoon* genes. A) An alternative alignment of the Core IZF domain obtained using Clustal W to align the domains illustrating that the IZF domain appears to be have been interrupted by an insertion in the teleost *pcloa* IZF domain. Compare this alignment to the alignment in Figure S9 obtained using MUSCLE. Formatting of the alignment figure was performed in Clustal X as described in methods. B) Alignment of *bassoon* gene zinc finger exons demonstrating that the core IZF domain is largely conserved in the *bassoon* homologs. The alignment was obtained using MUSCLE and formatting of the alignment figure was performed in Clustal X as described in methods.

Figure S13 and S14 are available for download at

http://www.g3journal.org/lookup/suppl/doi:10.1534/g3.112.003624/-/DC1.

Figure S13 Alignments of the zinc finger repeats from percomorph fish. Alignment of the repeating zinc finger domain exons of stickleback, tilapia, medaka, fugu and green spotted puffer. The alignment was obtained using MUSCLE with gap opening and gap extension penalties of 4.9 and 0.01. Formatting of the alignment was performed in Clustal X as described in methods.

Figure S14 Alignments of zinc finger repeats of all teleosts examined. A larger alignment of the zinc finger repeat domain exons of all species examined in this paper (cod, zebrafish, stickleback, tilapia, medaka, fugu and green spotted puffer, lizard, mouse, frog and coelacanth). The alignment was obtained using MUSCLE with gap opening and gap extension penalties of 4.9 and 0.01. Formatting of the alignment was performed in Clustal X as described in methods.

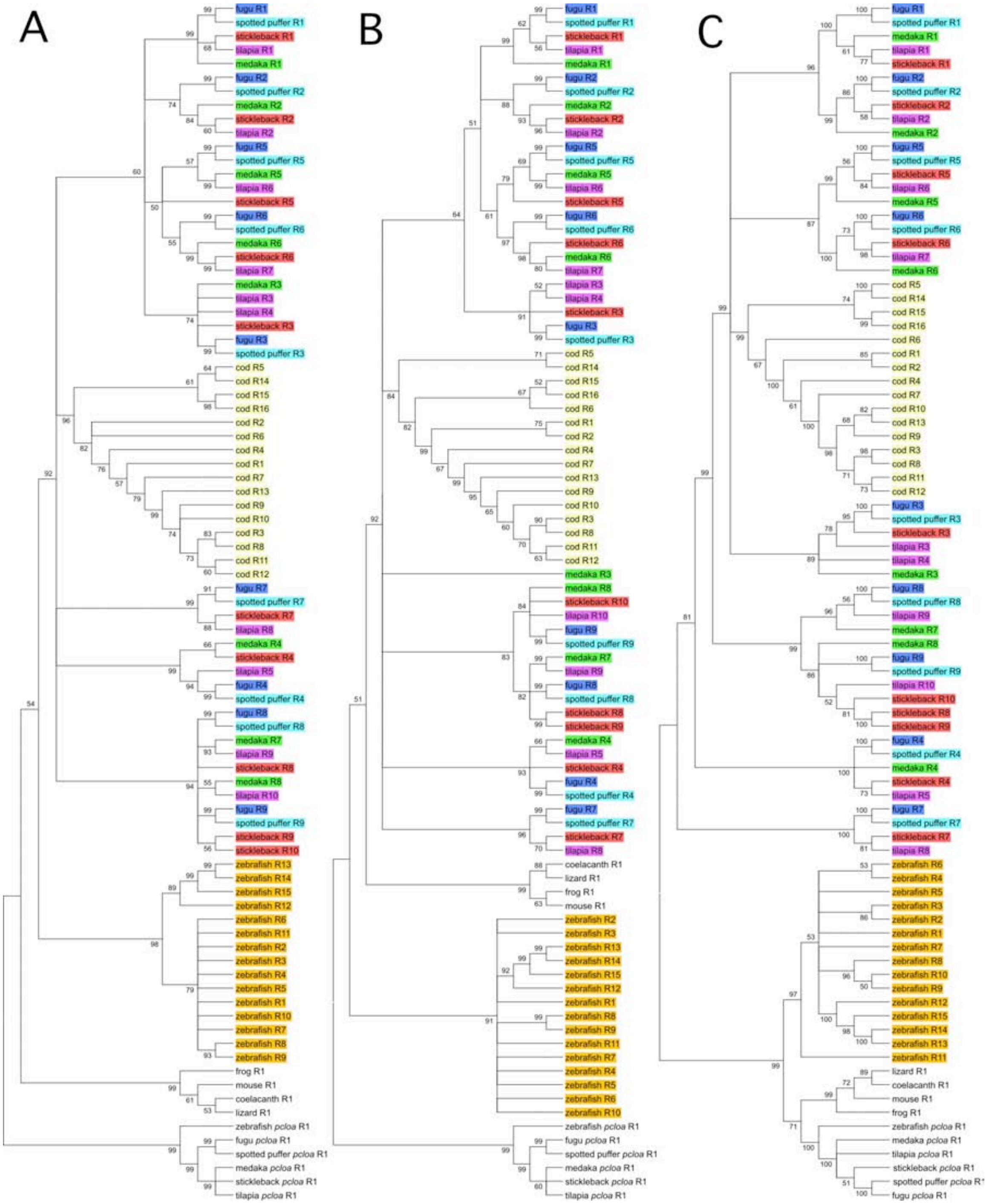

Figure S15 Comparison of evolutionary trees of repeated zinc finger domains obtained using distinct alignment methods and gap penalties. Tree trees constructed from alignments created by A) Clustal W alignment run within MEGA5 using default values for gap opening and extension without a negatively weighted substitution matrix. These are the default parameters for Clustal X stand alone application used in initial analysis of these sequences. B) MUSCLE run within MEGA5 under default parameters (-2.9 gap opening penalty, 0 gap extension penalty), and C) MUSCLE run under parameters used for the zinc finger repeat analysis presented in the rest of the paper (-4.9 gap opening penalty, -0.01 gap extension penalty).

Figure S16 and S17 are available for download at

http://www.g3journal.org/lookup/suppl/doi:10.1534/g3.112.003624/-/DC1.

Figure S16 Alignment of Zinc finger Repeats using Clustal W. Alignment of the repeated exons encoding the zinc finger domains obtained from Clustal W under default conditions used in the construction of trees presented in Figure S15. Formatting of the alignment was performed in Clustal X as described in methods.

Figure S17 Alignment of Zinc finger Repeats using MUSCLE. Alignment of the repeated exons encoding the zinc finger domains obtained from MUSCLE under default conditions used in the construction of trees presented in Figure S15. Formatting of the alignment was performed in Clustal X as described in methods.

File S1

GenbankFiles.zip

Available for download at http://www.g3journal.org/lookup/suppl/doi:10.1534/g3.112.003624/-/DC1.

This compressed file contains annotated genomic sequences and cDNA sequences of all genes annotated and examined in this work in genbank format (.gb files). The files can also be open in ApE, a shareware multipurpose sequence analysis program. It also contains .fasta version of the protein sequence of all Bassoon and all Piccolo proteins analyzed in this manuscript as well as all individual files as DNA strider .xprt files.

Tables S1 and S3 are available for download at

http://www.g3journal.org/lookup/suppl/doi:10.1534/g3.112.003624/-/DC1.

Table S1 Piccolo gene splicing

An excel spreadsheet provides lists of the nucleotide start and stop position for each exon, the sequence of each 5' and 3' splice sit, the size of each exon, the size of each intron, and the translation of the exon, codons for all Piccolo genes that were annotated. The nucleotide positions are based upon publicly available reference WGS. Note that in the case of the green spotted puffer, tilapia and cod genomes, we have identified errors and characterized the sequence of gap in the WGS. These are changes delineated in the materials and methods section. In these cases, the exons start and stop position in this table are based on the unmodified sequence and for the relevant exons will not yield the translations listed in the column. These columns are italicized and highlighted in red to warn the users.

Table S2 Bassoon gene splicing

An Excel spreadsheet provides lists of the nucleotide start and stop position for each exon, the sequence of each 5' and 3' splice sit, the size of each exon, the size of each intron, and the translation of the exon, codons for all Piccolo genes that were annotated. The nucleotide positions are based upon publicly available reference WGS.

Table S3 Oligonucleotides used in this study

A list of oligonucleotides used in the study for 1) characterization of the splicing of zebrafish *pclob* by RT-PCR, 2) verify the identification of cod and green spotted puffer genomic samples, 3) to isolation of PCR products from tilapia, fugu, and cod repeat exons to correct sequencing errors and fill in missing WGS data, 4) to amplify from cDNA templates for the construction of cRNA probes for in situ hybridization.

primer	Sequence 5'- 3'	purpose
4721	CWCCCAAAGCYRRNATTCTWAAYTAAACTA	amplify and sequence mitochondrial genome D-Loop region
4722	CATAGTGGGGTATCTAATCCCAGTTTG	amplify and sequence mitochondrial genome D-Loop region
4703	GCCTCACTGAAGAATGTCCCTCA	amplify and sequence cod zinc finger repeat 13
4704	GGTGACACACTGTTCAGATTCAAGC	amplify and sequence cod zinc finger repeat 13
4705	GTCTCAGAACCAGGGCCAGGA	amplify and sequence spotted puffer zinc finger repeat 2/3
4606	CAGAAACCTCATTTAGAGCAGTTAGGGG	amplify and sequence spotted puffer zinc finger repeat 2/3
4707	CCTGATTTCAAATAAGTTGGGACAGTATGTAAGAG	amplify and sequence spotted puffer zinc finger repeat 8
4708	GTGGATTTAGTCCTATGCCAAGCCTA	amplify and sequence spotted puffer zinc finger repeat 8
4717	AGCCTTCTGCAGAGACTCCCAAGGG	amplify and sequence tilapia zinc finger repeat 9
4718	CCTCCGTTTTCTGGACAGGCGGTGG	amplify and sequence tilapia zinc finger repeat 9
4768	ACCTTGGTGGGAAGTCCTGGT	amplify and sequence spotted puffer zinc finger repeat 4
4769	CTTTGTCTTTACAGTCCTGAGC	amplify and sequence spotted puffer zinc finger repeat 4
4853	GCTCTGTCCAGTGTGCAAGACTG	amplify and sequence *bassoon b* cDNA fragment
4854	CAGCTGGCTTTTGCTGCTGAGG	amplify and sequence *bassoon b* cDNA fragment
4855	CACCATAGCAGCTGCCGTTACAC	amplify and sequence *piccolo b* cDNA fragment
4856	TGCTGCAGCCTGGTCTTGTTC	amplify and sequence *piccolo b* cDNA fragment
4582	CCTCTGCCAAGATCACAACACCC	amplify zebrafish zinc finger repeat R1|R2 junction from cDNA
4583	CTGACTGTTCTTGTTCAGCCTCAGC	amplify zebrafish zinc finger repeat R1|R2 junction from cDNA
4598	GGACCTTGCCAACTACCAGATCTG	amplify zebrafish zinc finger repeat R2|3, R3|4 junctions from cDNA
4605	AGACTGCAACTTAGGATCTAGACTTGAAG	amplify zebrafish zinc finger repeat R2|3, R3|4 junctions from cDNA
4599	CAGCTGTTAAGTCCTCTCAGCTTCTTG	amplify zebrafish zinc finger repeat R4|5, R5|6 junctions from cDNA
4604	CGAGAACTAGACGGTGTTGTGGAGG	amplify zebrafish zinc finger repeat R4|5, R5|6 junctions from cDNA
4597	CCCATGGTCAGTCAGGCACAG	amplify zebrafish zinc finger repeat R6|7 junction from cDNA
4584	GGAGATGGCGATCTAGAGCGTCC	amplify zebrafish zinc finger repeat R6|7 junction from cDNA
4596	GAATCCACCAAACTATAGCACATGTACTAC	amplify zebrafish zinc finger repeat R7|8, R8|9 junctions from cDNA
4603	GTGGAGATGCTGGTGCAAGTGC	amplify zebrafish zinc finger repeat R7|8, R8|9 junctions from cDNA
4595	CAGAACTACAGCACTTGTAGTTCATGCC	amplify zebrafish zinc finger repeat R9|10, R10|11 junctions from cDNA
4602	GGTTGAGATGCTTTGCTAGCCATTGG	amplify zebrafish zinc finger repeat R9|10, R10|11 junctions from cDNA
4594	GTGGATCTCAATATAGGTTCTAAGGTCACTCC	amplify zebrafish zinc finger repeat R11|12, R12|13 junctions from cDNA
4601	CCTGATCAGGTGCAGCCTTCTTC	amplify zebrafish zinc finger repeat R11|12, R12|13 junctions from

		cDNA
4593	CATCACCCATACCACTTCAAAAACTTGCTC	amplify zebrafish zinc finger repeat R13|14, R14|15 junctions from cDNA
4600	GTTTGGACTTAACTGGAGCTGGAGCTA	amplify zebrafish zinc finger repeat R13|14, R14|15 junctions from cDNA
4592	TCTGGAAGGGATATATTACTGACACATCCTG	amplify zebrafish zinc finger repeat R15|CC junction from cDNA
4585	GACAGAGATGCAGGGACTGCTGG	amplify zebrafish zinc finger repeat R15|CC junction from cDNA
4606	GTAATACGACTCACTATAGGCCACCTCAACCAACTCAGCAG	amplify template for sense cRNA in situ probe for zebrafish *piccolo a*
4607	AATTAACCCTCACTAAAGGGCATTCATTGCAACCATGGTCTGTG	amplify template for antisense cRNA in situ probe for zebrafish *piccolo a*
4576	GTAATACGACTCACTATAGGGTACCACTGGCATTCACACGG	amplify template for sense cRNA in situ probe for zebrafish *piccolo b*
4577	AATTAACCCTCACTAAAGGGCAGCAGTGACCACTGCTGCAG	amplify template for antisense cRNA in situ probe for zebrafish *piccolo b*
4578	GTAATACGACTCACTATAGGGTTGCGTCAGCTAAGGCTTCAG	amplify template for sense cRNA in situ probe for zebrafish chr 8 *bassoon a*
4579	AATTAACCCTCACTAAAGGGACCTTGCTAGGATCTGGTATGGTG	amplify template for antisense cRNA in situ probe for zebrafish chr 8 *bassoon a*
4580	GTAATACGACTCACTATAGGGCGTCAGCTACGTCTACAAGAAGAG	amplify template for sense cRNA in situ probe for zebrafish chr 11 *bassoon b*
4581	AATTAACCCTCACTAAAGGGCCCAACTGGTCTGACTCAGTCTG	amplify template for antisense cRNA in situ probe for zebrafish chr 11 *bassoon b*
