# Supplementary material for: A Window into Domain Amplification Through Piccolo in Teleost Fish
Source: G3 (Bethesda). 2012 Nov 1;2(11):1325–39. doi: 10.1534/g3.112.003624 (PMC3484663; doi:10.1534/g3.112.003624)
Supplement: Supporting Information [file supp_2.11.1325_FigureS1.pdf]

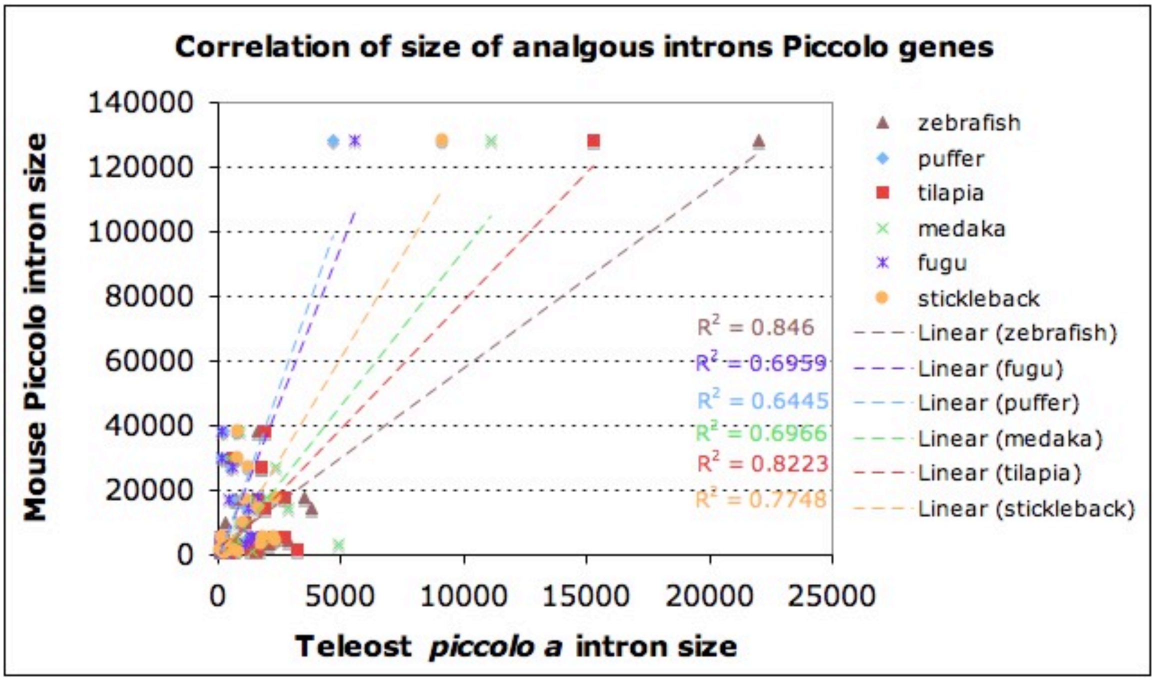

Figure S1 Correlation in size of analogous introns in mouse and teleost Piccolo genes. Mouse intron size was plotted against teleost intron size for the *pcloa* genes of zebrafish, green spotted puffer (listed as puffer in figure), fugu, medaka, tilapia and stickleback. A linear regression was performed and trend line and the correlation coefficient for each regression is presented.
