# Supplementary material for: A Window into Domain Amplification Through Piccolo in Teleost Fish
Source: G3 (Bethesda). 2012 Nov 1;2(11):1325–39. doi: 10.1534/g3.112.003624 (PMC3484663; doi:10.1534/g3.112.003624)
Supplement: Supporting Information [file supp_2.11.1325_FigureS2.pdf]

A

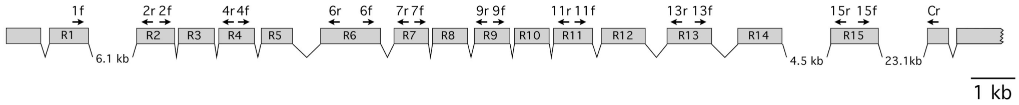

B

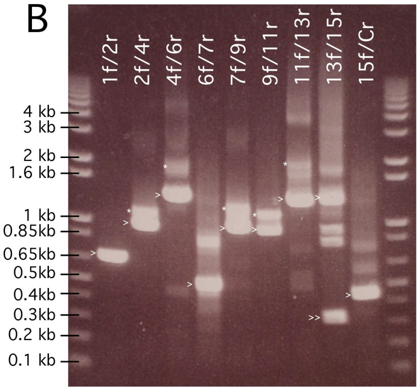

PCR primers	central exon junction tested	predicted size for cDNA (bp)	predicted size if exon skipped (bp)	expected genomic size (bp)
1f,2r	1|2	675	--	6797
2f,4r	2|3,3|4	977	166	1149
4f,6r	4|5,5|6	1384	571	2036
6f,7r	6|7	530	--	828
7f,9r	7|8,8|9	983	194	1218
9f,11r	9|10,10|11	960	176	1148
11f,13r	11|12,12|13	1309	319	1994
13f,15r	13|14,14|15	1326	330	4196
15f,Cr	15|CC	448	--	23640

Figure S2 RT-PCR analysis of splicing patterns in the repeated zebrafish zinc finger exons. A) Diagram of the exon structure of the repeated exons encoding zinc finger domains of zebrafish *pclob.* Name and position of oligonucleotide primers used for RT-PCR are also depicted above the representation of the genomic organization. The drawing is to scale except for the 3 large exons, the sizes of which are indicated in the drawing. A 1 kb scale bar is present on the right. B) Agarose gel separation of RT-PCR products obtained from amplification of first strand cDNA from 5-day-old zebrafish embryos. On the left is a table that tabulates predicted sizes of PCR products for genomic DNA, *pclob* cDNA, and cDNA size if an exon is omitted from the mature transcript. > mark DNA bands of the expected size if an exon is spliced as illustrated in A. * mark bands of the expected size of genomic DNA, >> mark the PCR products consistent with skipping of exon R14. The sizes of DNA markers are on the left.
