# Supplementary material for: A Window into Domain Amplification Through Piccolo in Teleost Fish
Source: G3 (Bethesda). 2012 Nov 1;2(11):1325–39. doi: 10.1534/g3.112.003624 (PMC3484663; doi:10.1534/g3.112.003624)
Supplement: Supporting Information [file supp_2.11.1325_FigureS4.pdf]

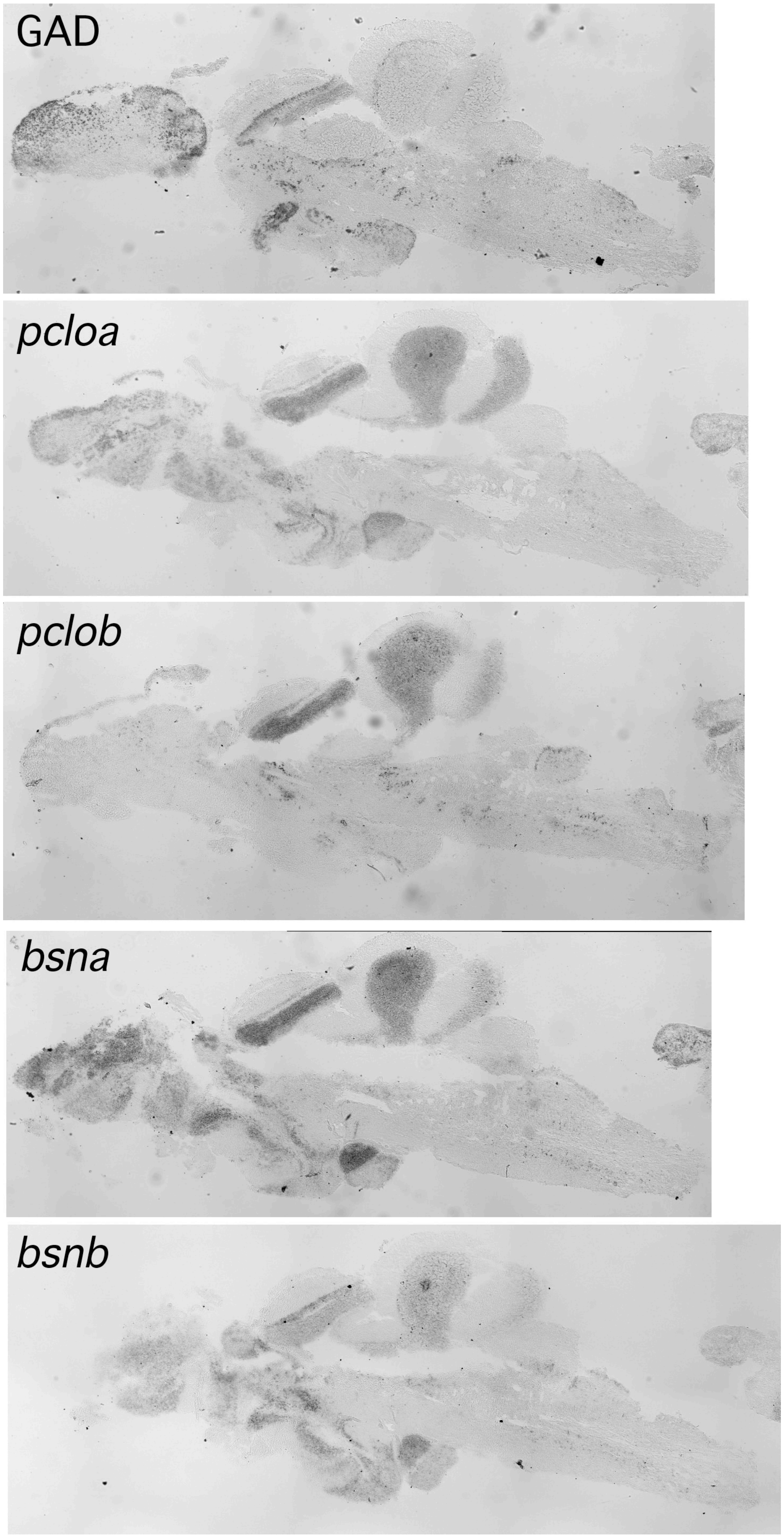
GAD
pcloa
pclob
bsna
bsnb

Figure S4 Zebrafish *piccolo* and *bassoon* expression as determined by in situ hybridization of adult brain. Sections of adult (~ 1 year old) adult wild type zebrafish brain were hybridized with DIG-labeled probes for zebrafish *pcloa*, *pclob*, *bsna*, *bsnb*, and zebrafish *gad1*. Hybridizing probes were detected using an alkaline phosphatase conjugated anti-DIG secondary antibodies, and alkaline phosphatase activity was detected using an NBT-BCIP colored precipitation reaction.
