# Supplementary material for: A Window into Domain Amplification Through Piccolo in Teleost Fish
Source: G3 (Bethesda). 2012 Nov 1;2(11):1325–39. doi: 10.1534/g3.112.003624 (PMC3484663; doi:10.1534/g3.112.003624)
Supplement: Supporting Information [file supp_2.11.1325_FigureS5.pdf]

A

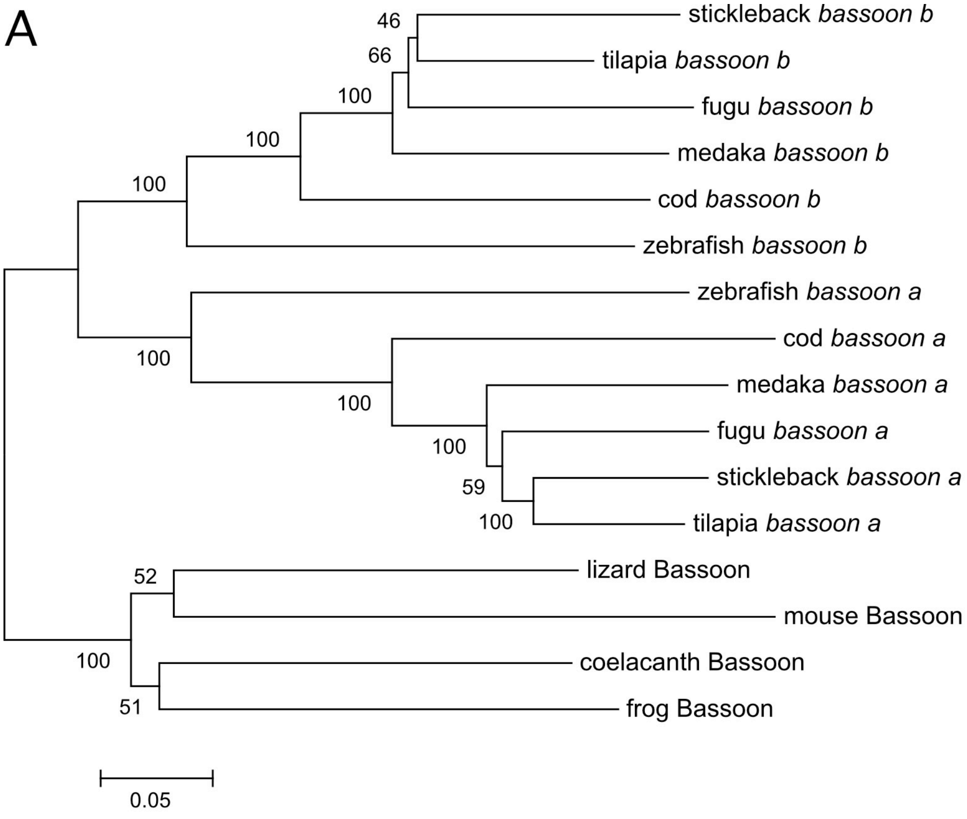
46
66
100
100
100
stickleback *bassoon b*
tilapia *bassoon b*
fugu *bassoon b*
medaka *bassoon b*
cod *bassoon b*
zebrafish *bassoon b*
zebrafish *bassoon a*
100
cod *bassoon a*
100
medaka *bassoon a*
100
fugu *bassoon a*
59
stickleback *bassoon a*
100
tilapia *bassoon a*
52
lizard Bassoon
mouse Bassoon
100
coelacanth Bassoon
51
frog Bassoon
0.05

B

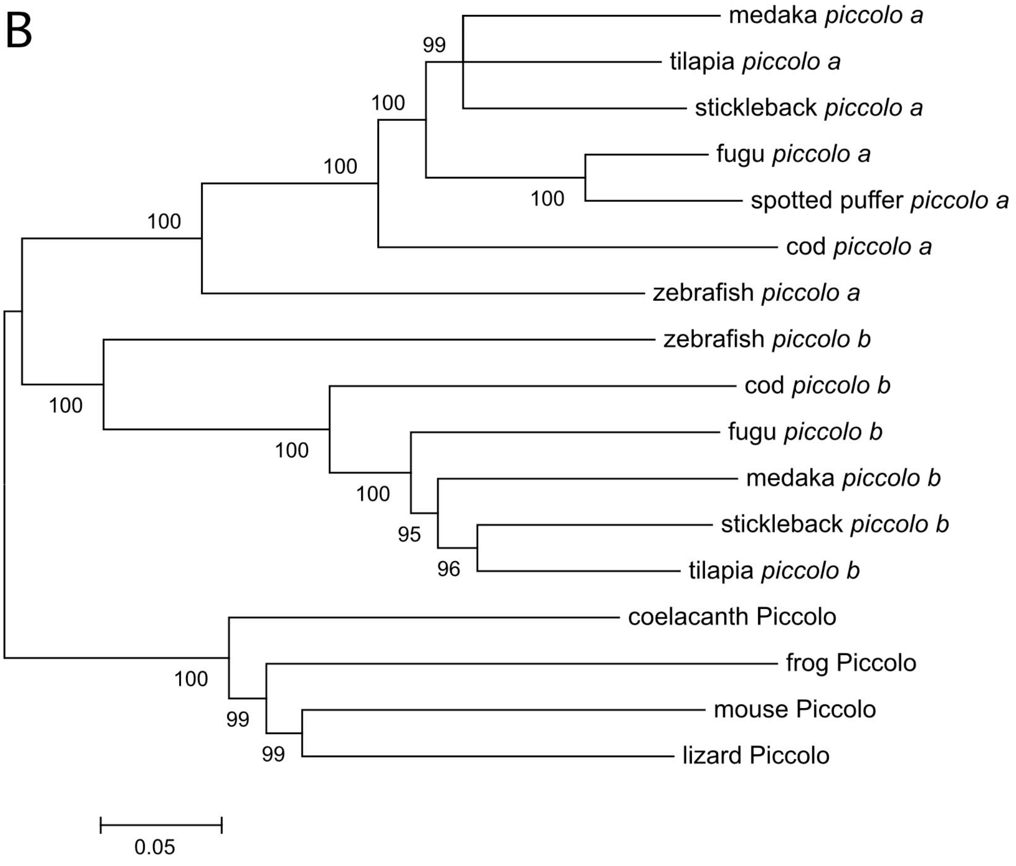
medaka *piccolo a*
99
tilapia *piccolo a*
100
stickleback *piccolo a*
fugu *piccolo a*
100
100
spotted puffer *piccolo a*
100
cod *piccolo a*
zebrafish *piccolo a*
zebrafish *piccolo b*
cod *piccolo b*
100
fugu *piccolo b*
100
medaka *piccolo b*
100
stickleback *piccolo b*
95
96
tilapia *piccolo b*
coelacanth Piccolo
frog Piccolo
100
mouse Piccolo
99
99
lizard Piccolo
0.05

Figure S5 Evolutionary Trees constructed from Teleost *piccolo* and *bassoon* genes. Evolutionary history was inferred from sequences using a neighbor-joining method. Numbers adjacent to the internal branches indicate bootstrap values. The trees are drawn to scale, with the branch length unit as base substitutions/site. Trees were constructed only from sequences C-terminal to the zinc finger motifs because of the diversity in zinc finger number in the *piccolo* homologs. A) Tree obtained using *bassoon* homologs using sequences from exon 5 onward. The tree is very similar to that obtained for complete *bassoon* (see Figure S7). B) Tree obtained using *piccolo* sequences (exon 5 onward for non-teleosts and teleost *pcloa* family members, exon 11 onward for stickleback *pclob*, exon 12 onward for fugu and tilapia *pclob*, exon 13 onward for medaka *pclob*, exon 18 onward for zebrafish *pclob*, and exon 19 onward for cod *pclob*).
