# Supplementary material for: A Window into Domain Amplification Through Piccolo in Teleost Fish
Source: G3 (Bethesda). 2012 Nov 1;2(11):1325–39. doi: 10.1534/g3.112.003624 (PMC3484663; doi:10.1534/g3.112.003624)
Supplement: Supporting Information [file supp_2.11.1325_FigureS6.pdf]

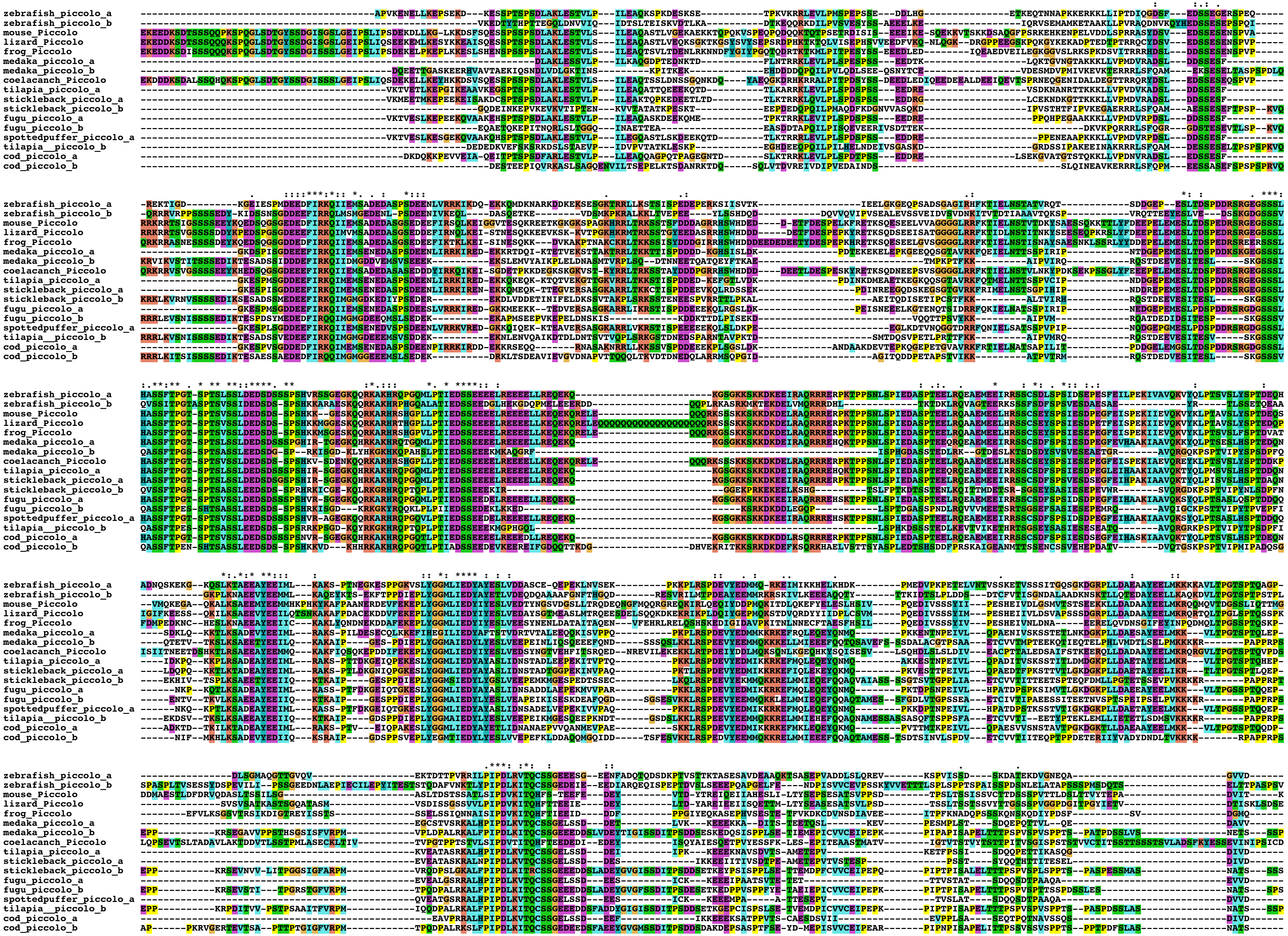
zebrafish_piccolo_a
zebrafish_piccolo_b
mouse_Piccolo
lizard_Piccolo
frog_Piccolo
medaka_piccolo_a
medaka_piccolo_b
coelacanch_Piccolo
tilapia_piccolo_a
stickleback_piccolo_a
stickleback_piccolo_b
fugu_piccolo_a
fugu_piccolo_b
spottedpuffer_piccolo_a
tilapia__piccolo_b
cod_piccolo_a
cod_piccolo_b

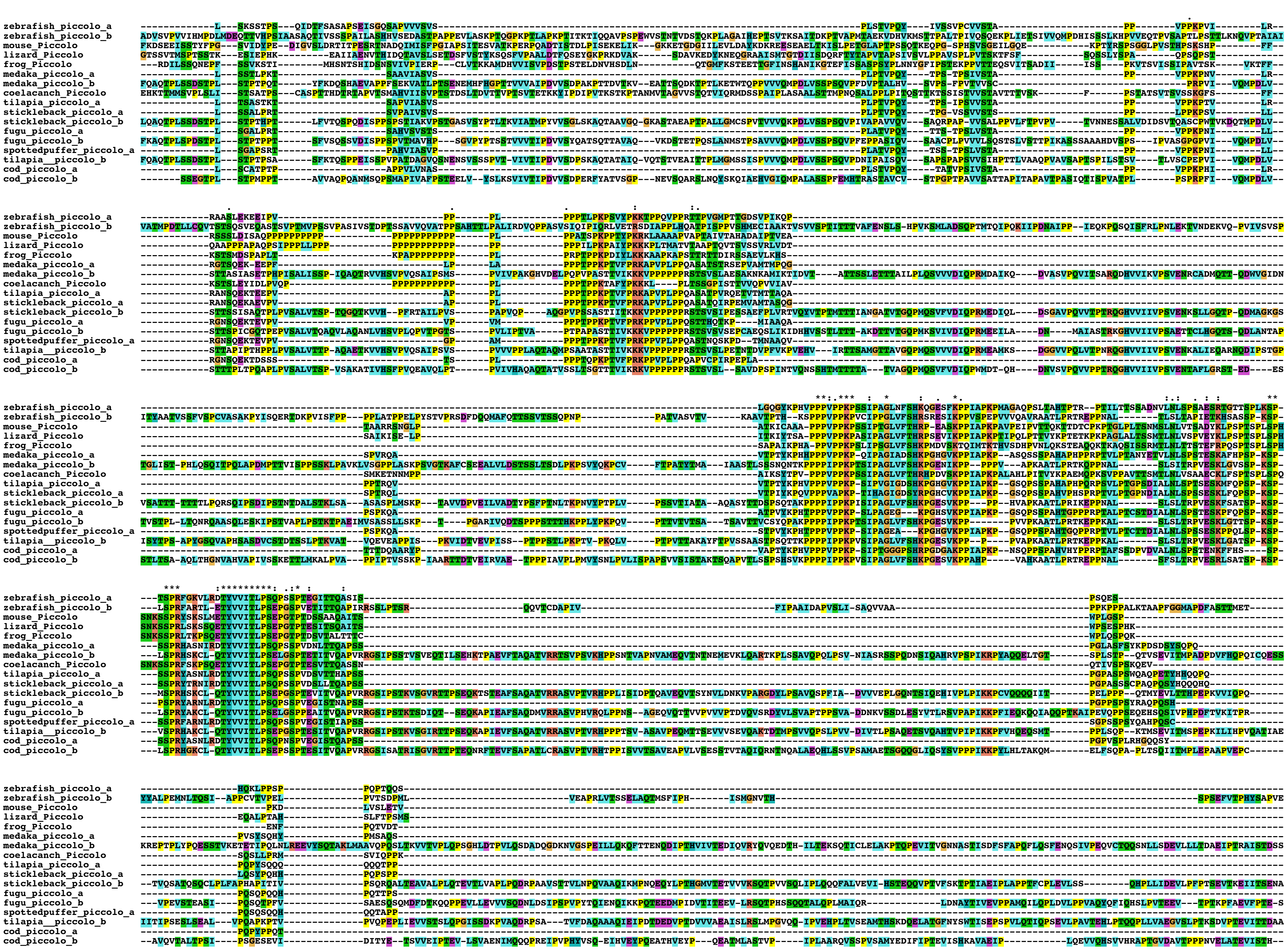

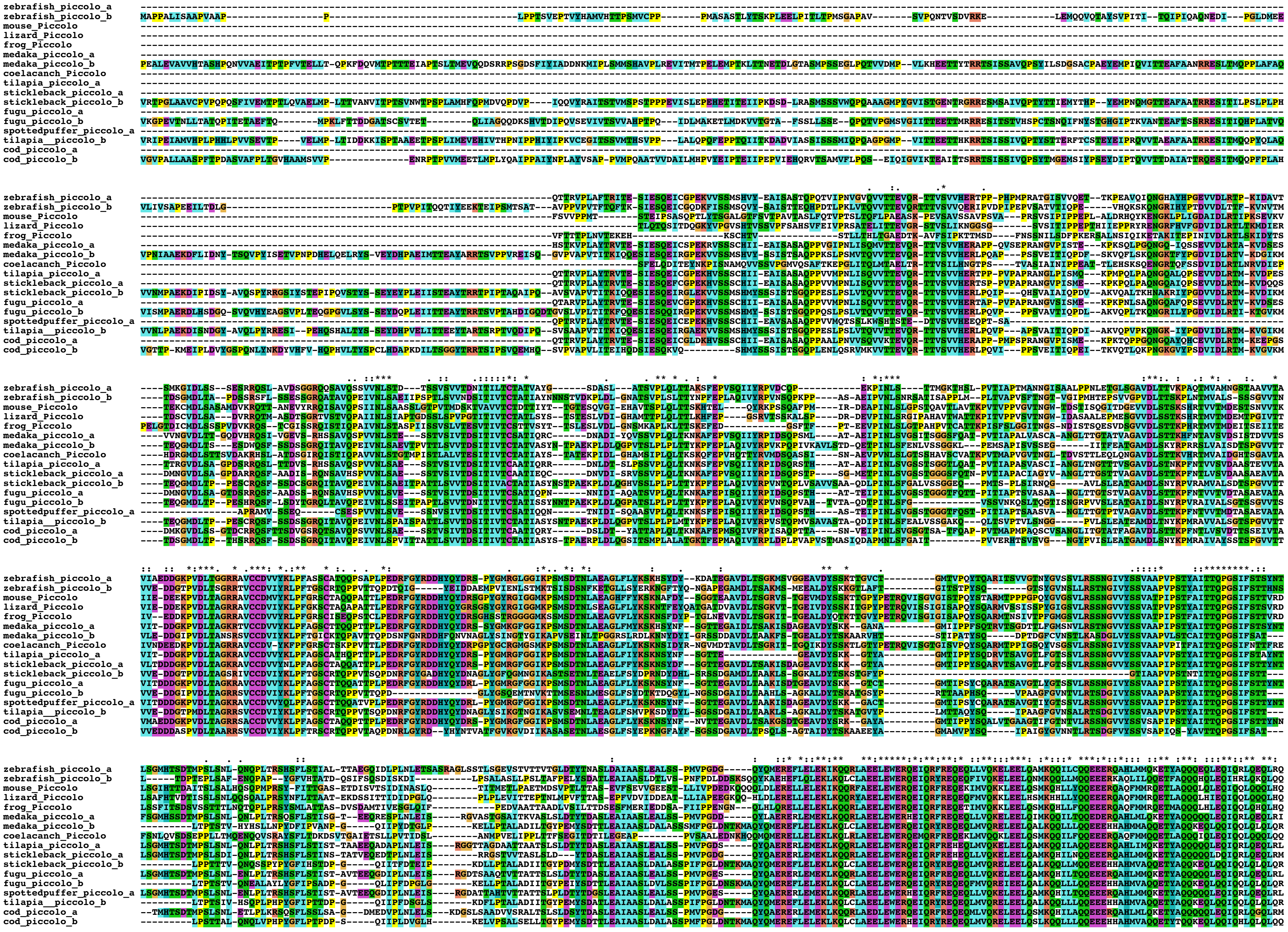
zebrafish_piccolo_a
zebrafish_piccolo_b
mouse_Piccolo
lizard_Piccolo
frog_Piccolo
medaka_piccolo_a
medaka_piccolo_b
coelacanch_Piccolo
tilapia_piccolo_a
stickleback_piccolo_a
stickleback_piccolo_b
fugu_piccolo_a
fugu_piccolo_b
spottedpuffer_piccolo_a
tilapia__piccolo_b
cod_piccolo_a
cod_piccolo_b

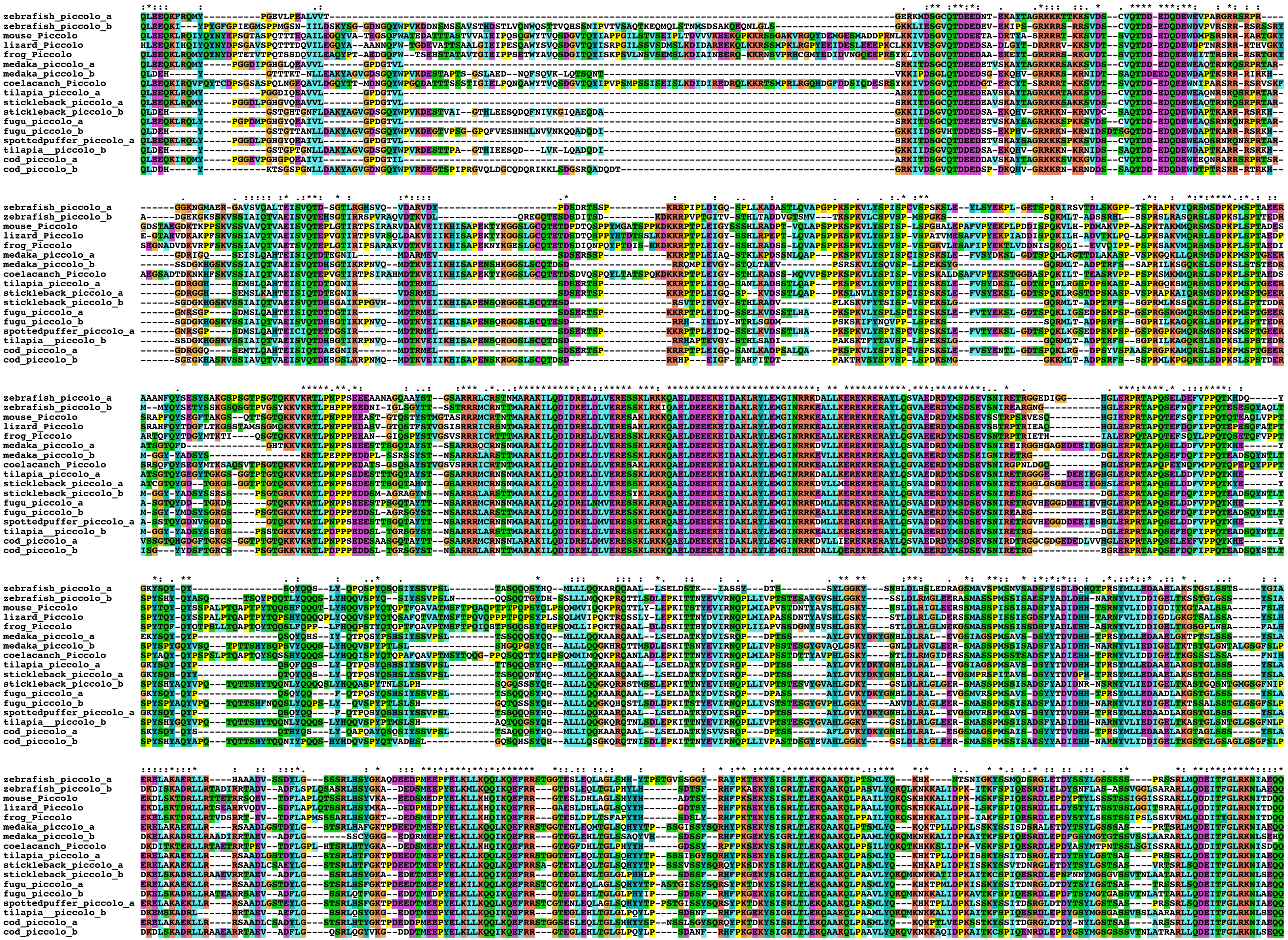
zebrafish_piccolo_a
zebrafish_piccolo_b
mouse_Piccolo
lizard_Piccolo
frog_Piccolo
medaka_piccolo_a
medaka_piccolo_b
coelacanch_Piccolo
tilapia_piccolo_a
stickleback_piccolo_a
stickleback_piccolo_b
fugu_piccolo_a
fugu_piccolo_b
spottedpuffer_piccolo_a
tilapia__piccolo_b
cod_piccolo_a
cod_piccolo_b

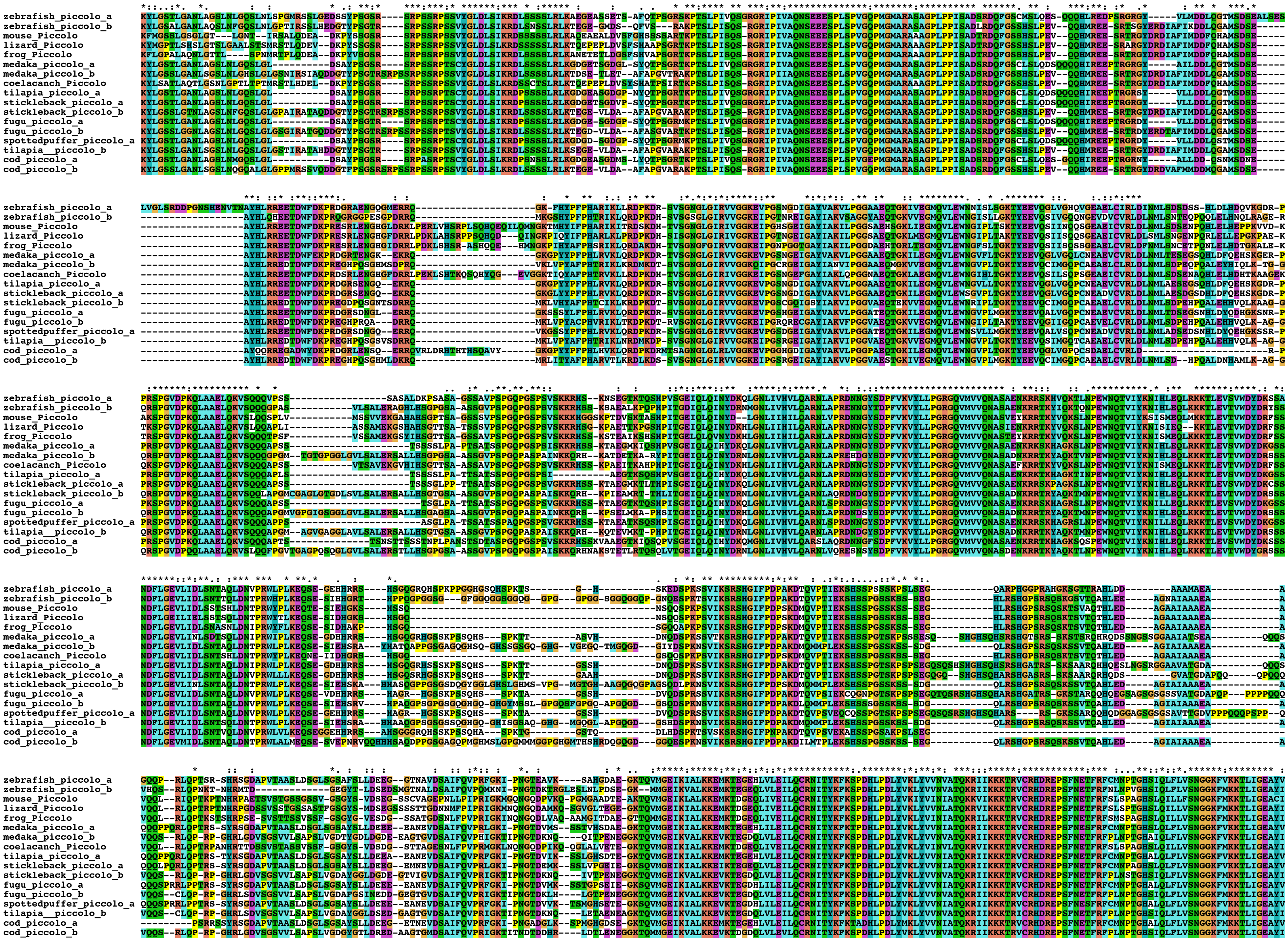
zebrafish_piccolo_a
zebrafish_piccolo_b
mouse_Piccolo
lizard_Piccolo
frog_Piccolo
medaka_piccolo_a
medaka_piccolo_b
coelacanch_Piccolo
tilapia_piccolo_a
stickleback_piccolo_a
stickleback_piccolo_b
fugu_piccolo_a
fugu_piccolo_b
spottedpuffer_piccolo_a
tilapia__piccolo_b
cod_piccolo_a
cod_piccolo_b

```
                           ****:*:*** *.****. :.   :
zebrafish_piccolo_a        WLDKVDLRKRVVSWHKLLASTAQIHS
zebrafish_piccolo_b        WLDKVDMRKRAVSWHKLVPTTSQTQP
mouse_Piccolo              WLDKVDLRKRIVNWHKLLMSPTQTH-
lizard_Piccolo             WLDKVDLRKRIVNWHKLLVSSTQTH-
frog_Piccolo               WLDKVDLRKRIVNWHKLLVSSAPAH-
medaka_piccolo_a           WLDKIDLRKRVVSWHKLLASTAQIHS
medaka_piccolo_b           WLDKVDMRKRVVSWHKLFVSSTQTNP
coelacanch_Piccolo         WLDKVDLRKRVVNWHKLLVSSTQSH-
tilapia_piccolo_a          WLDKVDLRKRVVSWHKLLASTAQIHS
stickleback_piccolo_a      WLDKVDLRKRVVGWHKLLASTAQIHS
stickleback_piccolo_b      WLDKVDMRKRVVSWHKLFVSSTQTNA
fugu_piccolo_a             WLDKVDLRKRVVSWHKLLASTAQIHS
fugu_piccolo_b             WLDKVDMRKRVVSWHKLFVSSTQTNP
spottedpuffer_piccolo_a    WLDKVDLRKRVVSWHKLLASTAQIHS
tilapia__piccolo_b         WLDKVDMRKRVVSWHKLFVSSTQTNP
cod_piccolo_a              WLDKVDLRKRVVSWHKLLASTAQIHS
cod_piccolo_b              WLDKVDMRKRVVSWHKLLVSTNQSNL
```
