# Supplementary material for: A Window into Domain Amplification Through Piccolo in Teleost Fish
Source: G3 (Bethesda). 2012 Nov 1;2(11):1325–39. doi: 10.1534/g3.112.003624 (PMC3484663; doi:10.1534/g3.112.003624)
Supplement: Supporting Information [file supp_2.11.1325_FigureS7.pdf]

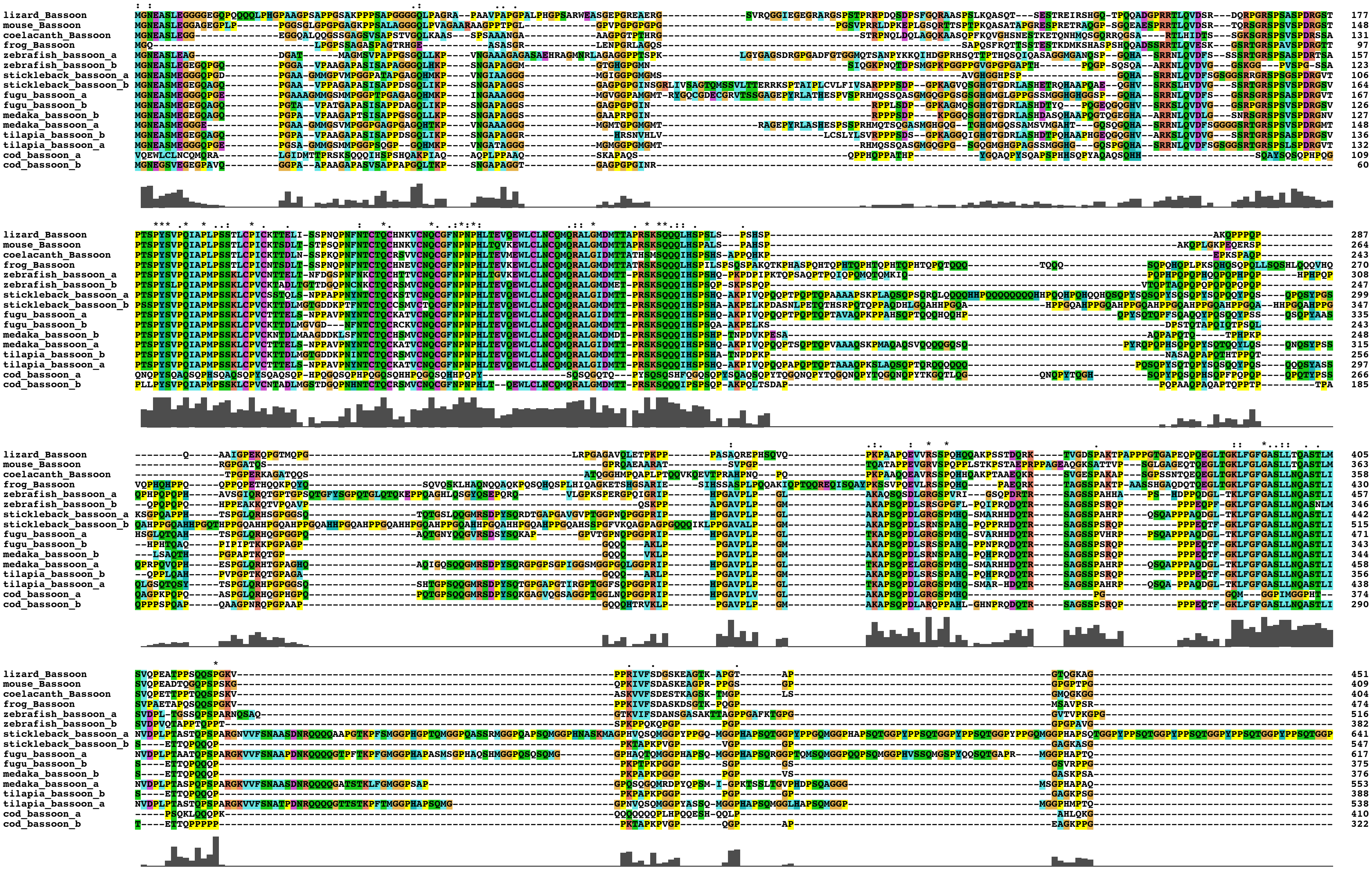

lizard_Bassoon 177
mouse_Bassoon 148
coelacanth_Bassoon 131
frog_Bassoon 97
zebrafish_bassoon_a 157
zebrafish_bassoon_b 123
stickleback_bassoon_a 106
stickleback_bassoon_b 164
fugu_bassoon_a 167
fugu_bassoon_b 126
medaka_bassoon_b 127
medaka_bassoon_a 148
tilapia_bassoon_b 136
tilapia_bassoon_a 132
cod_bassoon_a 109
cod_bassoon_b 60
lizard_Bassoon 287
mouse_Bassoon 264
coelacanth_Bassoon 243
frog_Bassoon 270
zebrafish_bassoon_a 308
zebrafish_bassoon_b 247
stickleback_bassoon_a 299
stickleback_bassoon_b 347
fugu_bassoon_a 335
fugu_bassoon_b 243
medaka_bassoon_b 248
medaka_bassoon_a 315
tilapia_bassoon_b 256
tilapia_bassoon_a 297
cod_bassoon_a 266
cod_bassoon_b 185
lizard_Bassoon 405
mouse_Bassoon 363
coelacanth_Bassoon 358
frog_Bassoon 430
zebrafish_bassoon_a 457
zebrafish_bassoon_b 346
stickleback_bassoon_a 442
stickleback_bassoon_b 515
fugu_bassoon_a 471
fugu_bassoon_b 343
medaka_bassoon_b 344
medaka_bassoon_a 458
tilapia_bassoon_b 356
tilapia_bassoon_a 438
cod_bassoon_a 374
cod_bassoon_b 290
lizard_Bassoon 451
mouse_Bassoon 409
coelacanth_Bassoon 404
frog_Bassoon 474
zebrafish_bassoon_a 516
zebrafish_bassoon_b 382
stickleback_bassoon_a 641
stickleback_bassoon_b 547
fugu_bassoon_a 617
fugu_bassoon_b 375
medaka_bassoon_b 376
medaka_bassoon_a 553
tilapia_bassoon_b 388
tilapia_bassoon_a 538
cod_bassoon_a 410
cod_bassoon_b 322

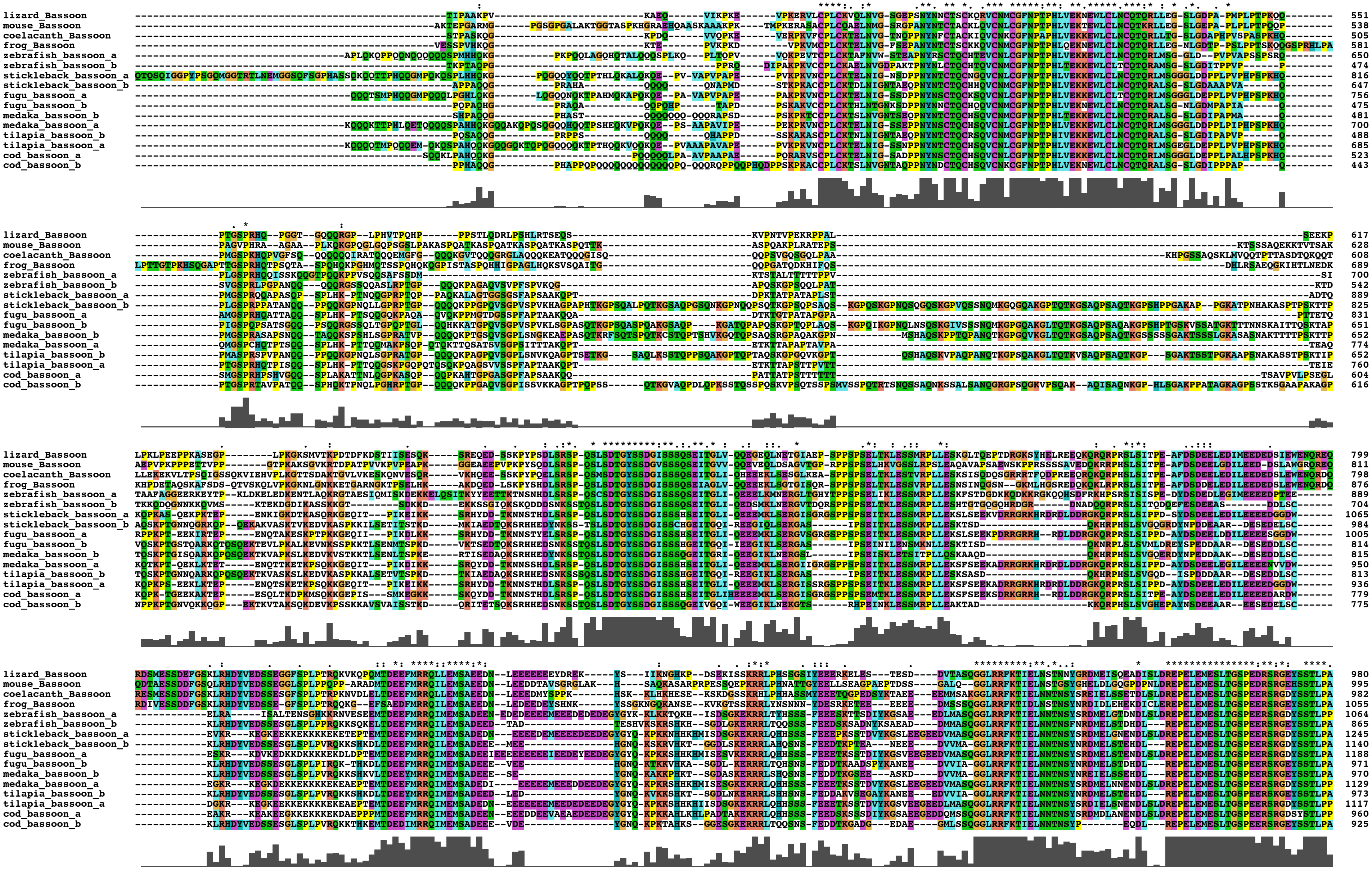
lizard_Bassoon
mouse_Bassoon
coelacanth_Bassoon
frog_Bassoon
zebrafish_bassoon_a
zebrafish_bassoon_b
stickleback_bassoon_a
stickleback_bassoon_b
fugu_bassoon_a
fugu_bassoon_b
medaka_bassoon_b
medaka_bassoon_a
tilapia_bassoon_b
tilapia_bassoon_a
cod_bassoon_a
cod_bassoon_b

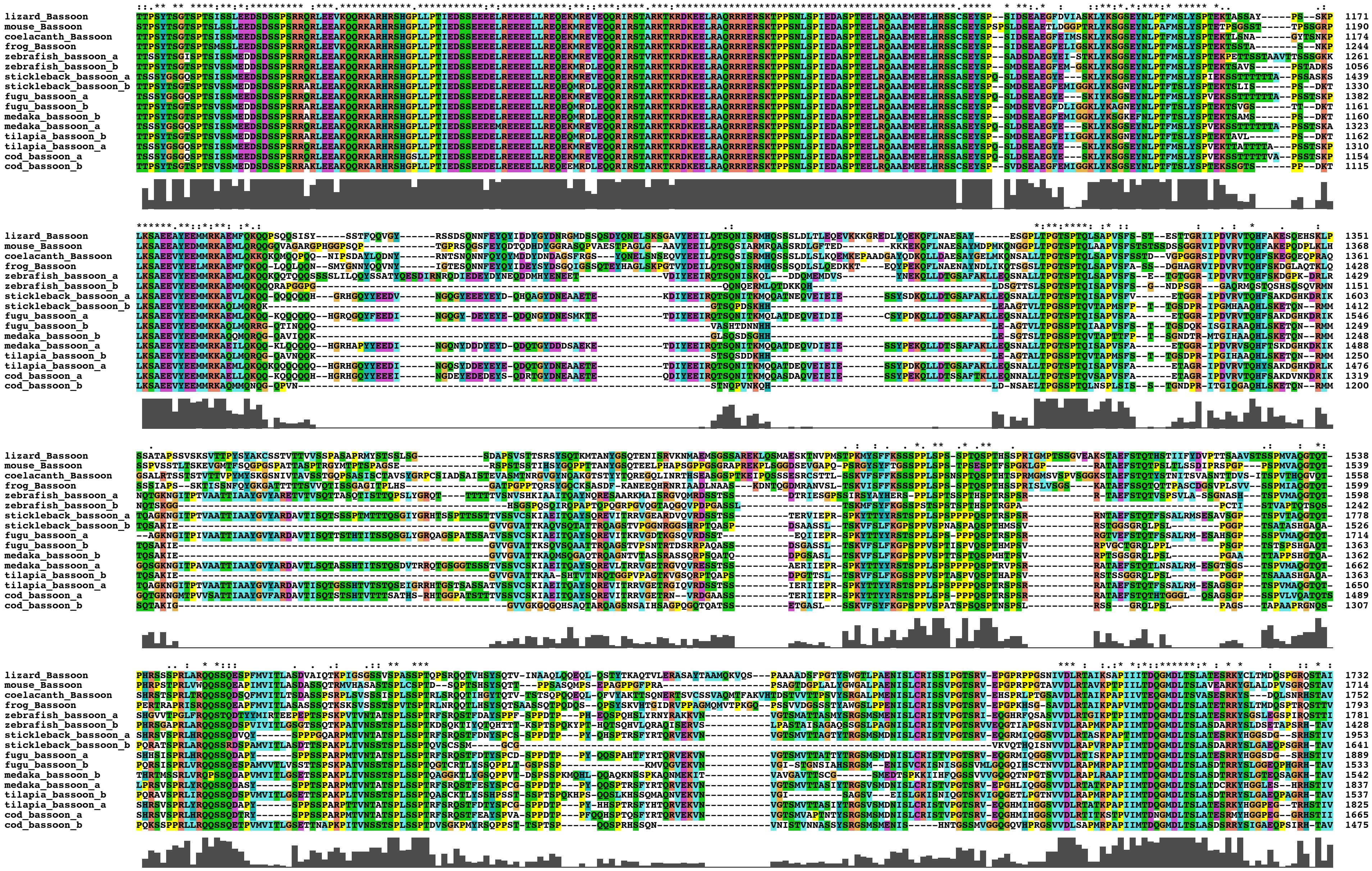

lizard_Bassoon 1171
mouse_Bassoon 1190
coelacanth_Bassoon 1174
frog_Bassoon 1244
zebrafish_bassoon_a 1261
zebrafish_bassoon_b 1056
stickleback_bassoon_a 1439
stickleback_bassoon_b 1330
fugu_bassoon_a 1382
fugu_bassoon_b 1161
medaka_bassoon_b 1160
medaka_bassoon_a 1323
tilapia_bassoon_b 1162
tilapia_bassoon_a 1310
cod_bassoon_a 1154
cod_bassoon_b 1115
lizard_Bassoon 1351
mouse_Bassoon 1368
coelacanth_Bassoon 1361
frog_Bassoon 1428
zebrafish_bassoon_a 1429
zebrafish_bassoon_b 1151
stickleback_bassoon_a 1603
stickleback_bassoon_b 1412
fugu_bassoon_a 1546
fugu_bassoon_b 1249
medaka_bassoon_b 1248
medaka_bassoon_a 1488
tilapia_bassoon_b 1250
tilapia_bassoon_a 1476
cod_bassoon_a 1319
cod_bassoon_b 1200
lizard_Bassoon 1538
mouse_Bassoon 1539
coelacanth_Bassoon 1558
frog_Bassoon 1599
zebrafish_bassoon_a 1598
zebrafish_bassoon_b 1242
stickleback_bassoon_a 1778
stickleback_bassoon_b 1526
fugu_bassoon_a 1714
fugu_bassoon_b 1363
medaka_bassoon_b 1362
medaka_bassoon_a 1662
tilapia_bassoon_b 1363
tilapia_bassoon_a 1650
cod_bassoon_a 1489
cod_bassoon_b 1307
lizard_Bassoon 1732
mouse_Bassoon 1714
coelacanth_Bassoon 1752
frog_Bassoon 1793
zebrafish_bassoon_a 1781
zebrafish_bassoon_b 1428
stickleback_bassoon_a 1953
stickleback_bassoon_b 1641
fugu_bassoon_a 1889
fugu_bassoon_b 1533
medaka_bassoon_b 1542
medaka_bassoon_a 1837
tilapia_bassoon_b 1537
tilapia_bassoon_a 1825
cod_bassoon_a 1665
cod_bassoon_b 1475

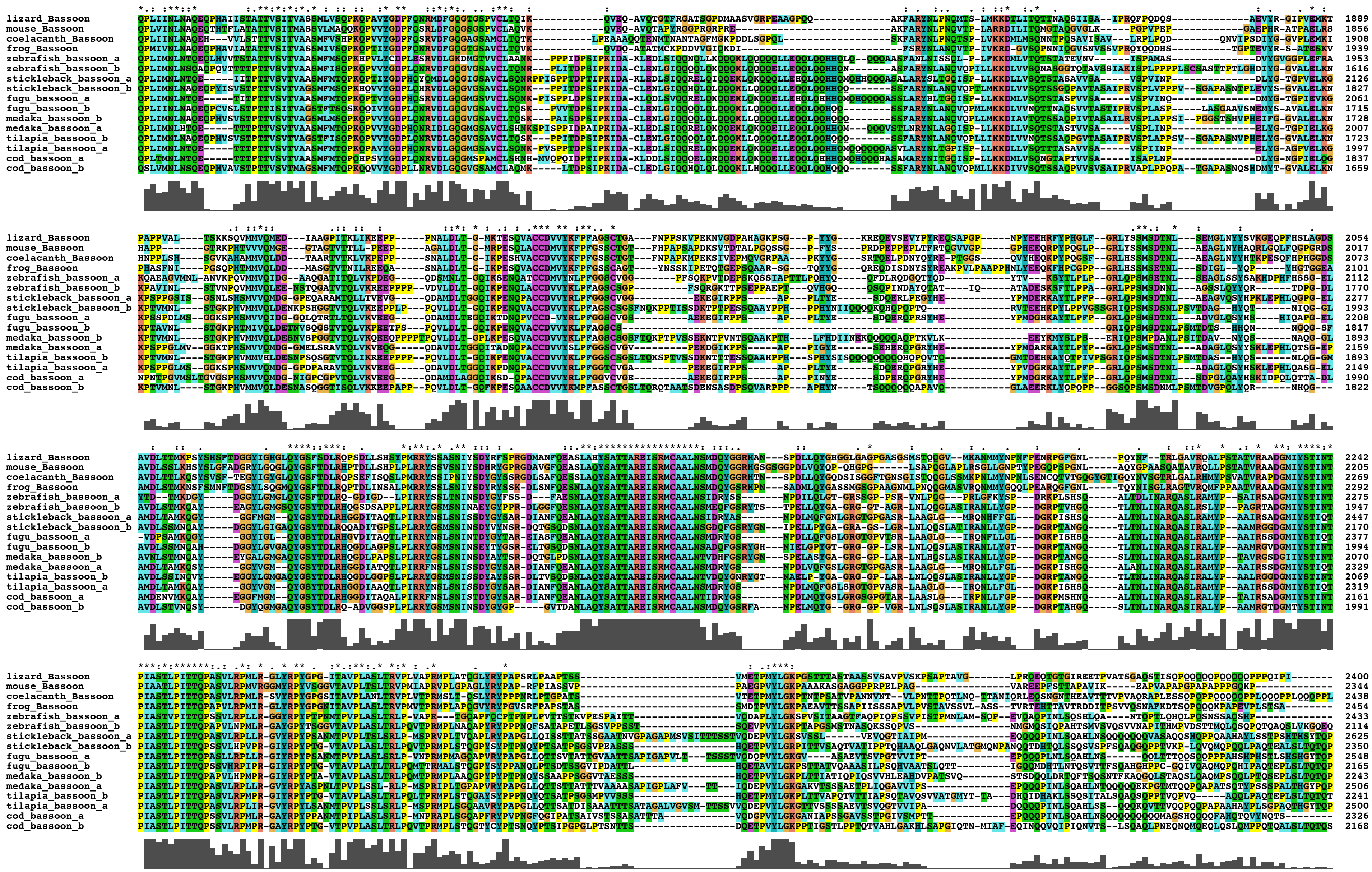
lizard_Bassoon 1889
mouse_Bassoon 1856
coelacanth_Bassoon 1908
frog_Bassoon 1939
zebrafish_bassoon_a 1953
zebrafish_bassoon_b 1616
stickleback_bassoon_a 2126
stickleback_bassoon_b 1827
fugu_bassoon_a 2061
fugu_bassoon_b 1715
medaka_bassoon_b 1728
medaka_bassoon_a 2007
tilapia_bassoon_b 1723
tilapia_bassoon_a 1997
cod_bassoon_a 1837
cod_bassoon_b 1659
lizard_Bassoon 2054
mouse_Bassoon 2017
coelacanth_Bassoon 2073
frog_Bassoon 2101
zebrafish_bassoon_a 2112
zebrafish_bassoon_b 1770
stickleback_bassoon_a 2277
stickleback_bassoon_b 1993
fugu_bassoon_a 2208
fugu_bassoon_b 1817
medaka_bassoon_b 1893
medaka_bassoon_a 2159
tilapia_bassoon_b 1893
tilapia_bassoon_a 2149
cod_bassoon_a 1990
cod_bassoon_b 1822
lizard_Bassoon 2242
mouse_Bassoon 2205
coelacanth_Bassoon 2269
frog_Bassoon 2292
zebrafish_bassoon_a 2275
zebrafish_bassoon_b 1947
stickleback_bassoon_a 2447
stickleback_bassoon_b 2170
fugu_bassoon_a 2377
fugu_bassoon_b 1994
medaka_bassoon_b 2070
medaka_bassoon_a 2329
tilapia_bassoon_b 2069
tilapia_bassoon_a 2319
cod_bassoon_a 2161
cod_bassoon_b 1991
lizard_Bassoon 2400
mouse_Bassoon 2344
coelacanth_Bassoon 2438
frog_Bassoon 2454
zebrafish_bassoon_a 2433
zebrafish_bassoon_b 2114
stickleback_bassoon_a 2625
stickleback_bassoon_b 2350
fugu_bassoon_a 2548
fugu_bassoon_b 2165
medaka_bassoon_b 2243
medaka_bassoon_a 2506
tilapia_bassoon_b 2241
tilapia_bassoon_a 2500
cod_bassoon_a 2326
cod_bassoon_b 2168

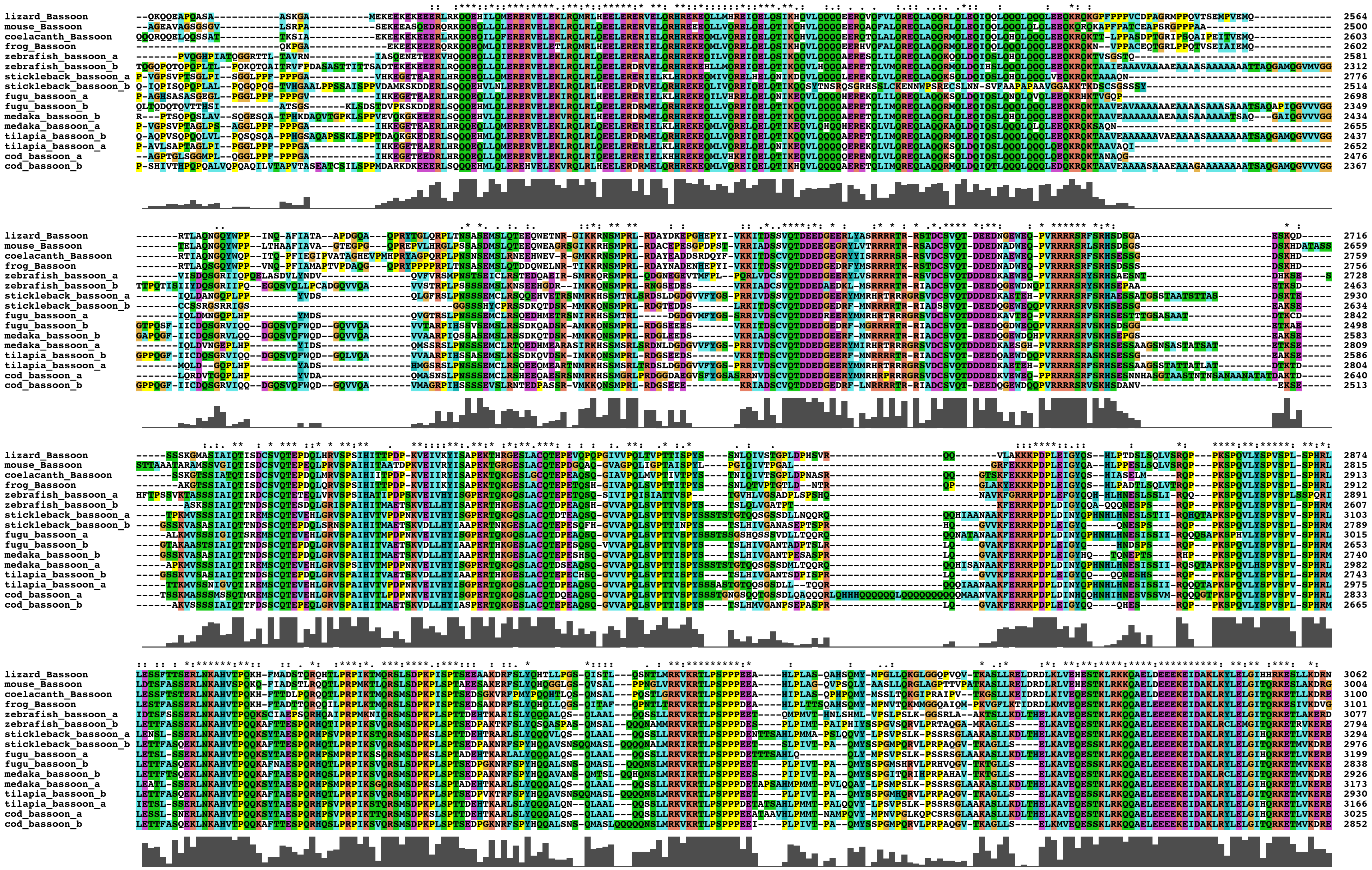
lizard_Bassoon
mouse_Bassoon
coelacanth_Bassoon
frog_Bassoon
zebrafish_bassoon_a
zebrafish_bassoon_b
stickleback_bassoon_a
stickleback_bassoon_b
fugu_bassoon_a
fugu_bassoon_b
medaka_bassoon_b
medaka_bassoon_a
tilapia_bassoon_b
tilapia_bassoon_a
cod_bassoon_a
cod_bassoon_b

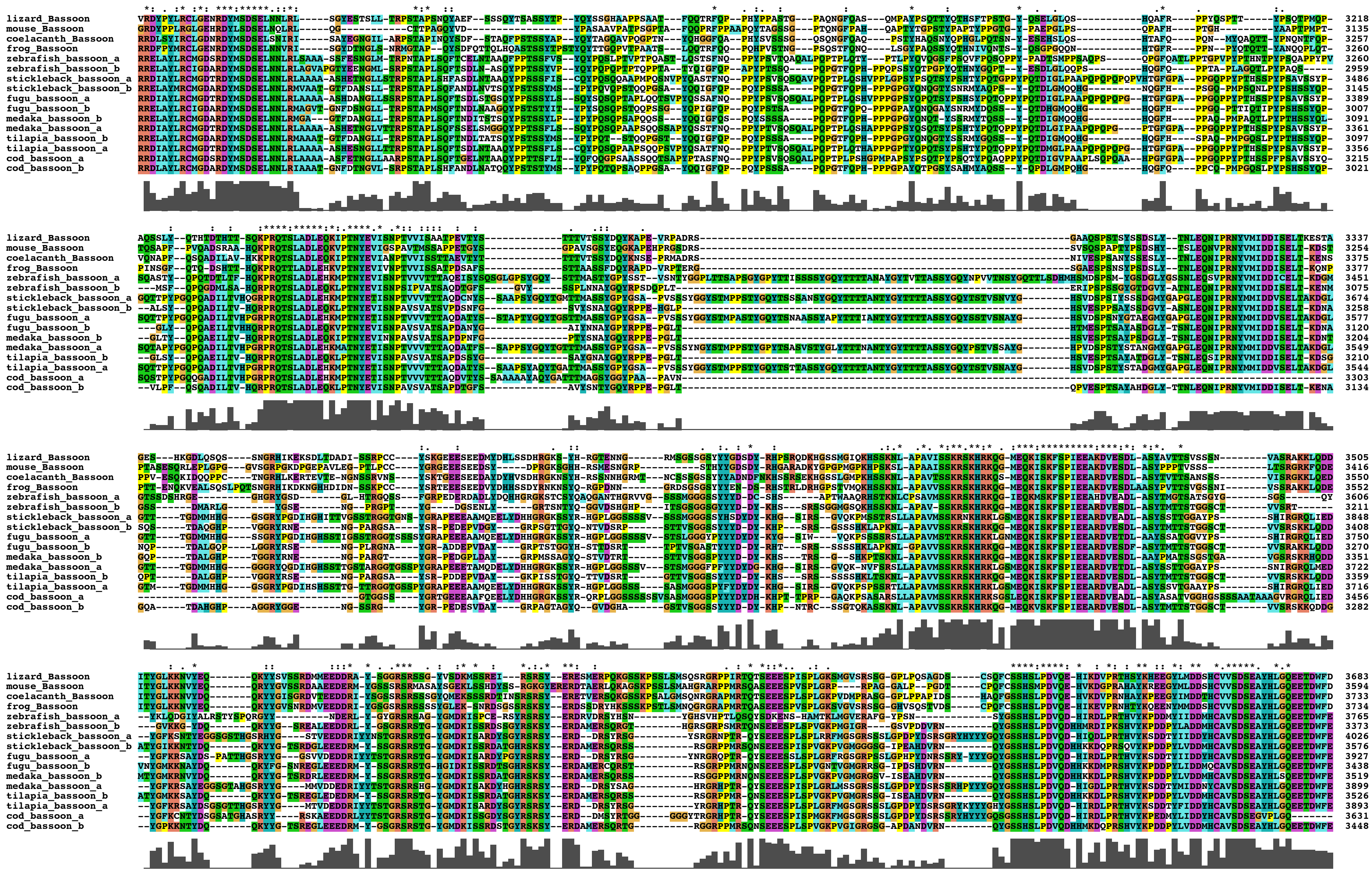
lizard_Bassoon
mouse_Bassoon
coelacanth_Bassoon
frog_Bassoon
zebrafish_bassoon_a
zebrafish_bassoon_b
stickleback_bassoon_a
stickleback_bassoon_b
fugu_bassoon_a
fugu_bassoon_b
medaka_bassoon_b
medaka_bassoon_a
tilapia_bassoon_b
tilapia_bassoon_a
cod_bassoon_a
cod_bassoon_b

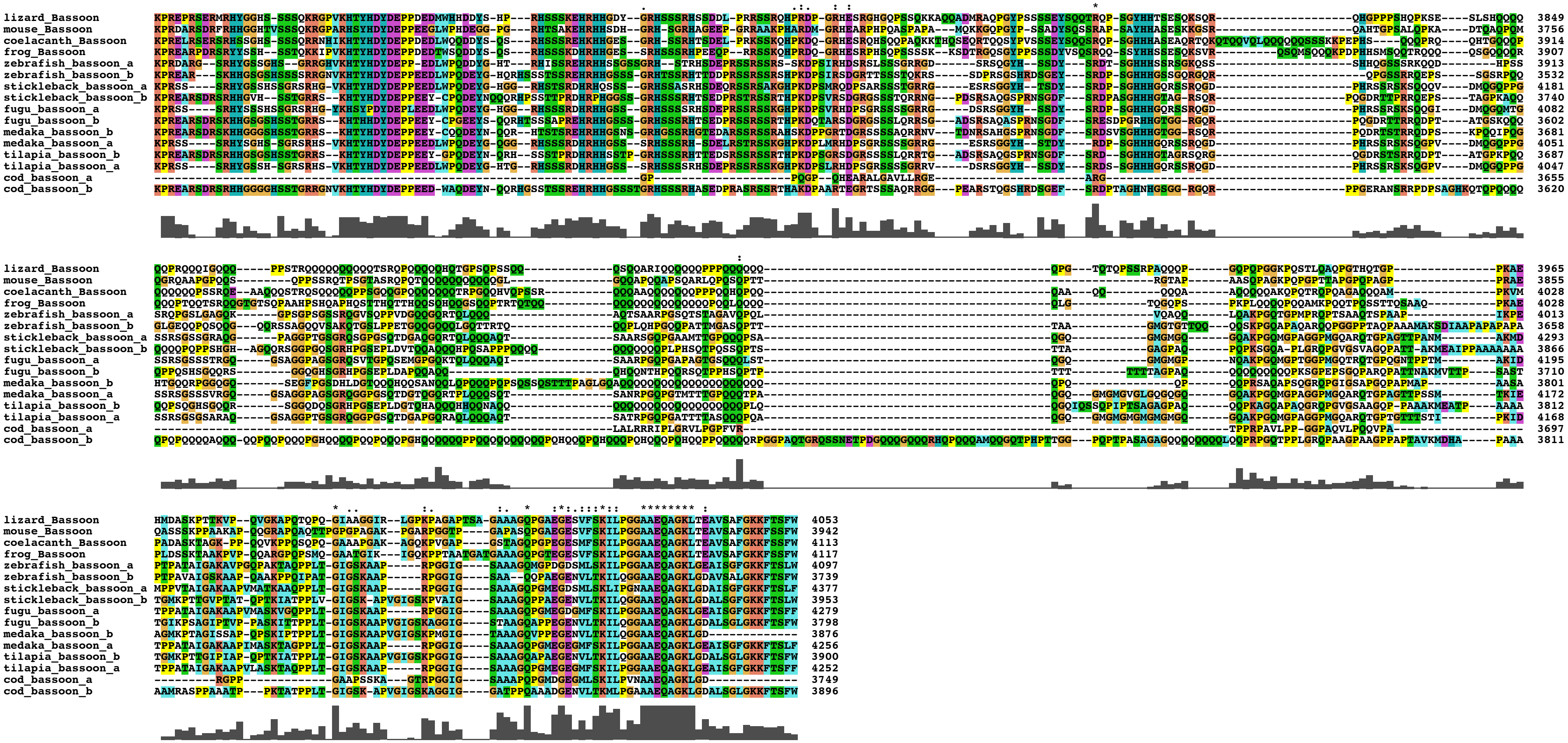
lizard_Bassoon
mouse_Bassoon
coelacanth_Bassoon
frog_Bassoon
zebrafish_bassoon_a
zebrafish_bassoon_b
stickleback_bassoon_a
stickleback_bassoon_b
fugu_bassoon_a
fugu_bassoon_b
medaka_bassoon_b
medaka_bassoon_a
tilapia_bassoon_b
tilapia_bassoon_a
cod_bassoon_a
cod_bassoon_b
