# Supplementary material for: A Window into Domain Amplification Through Piccolo in Teleost Fish
Source: G3 (Bethesda). 2012 Nov 1;2(11):1325–39. doi: 10.1534/g3.112.003624 (PMC3484663; doi:10.1534/g3.112.003624)
Supplement: Supporting Information [file supp_2.11.1325_FigureS8.pdf]

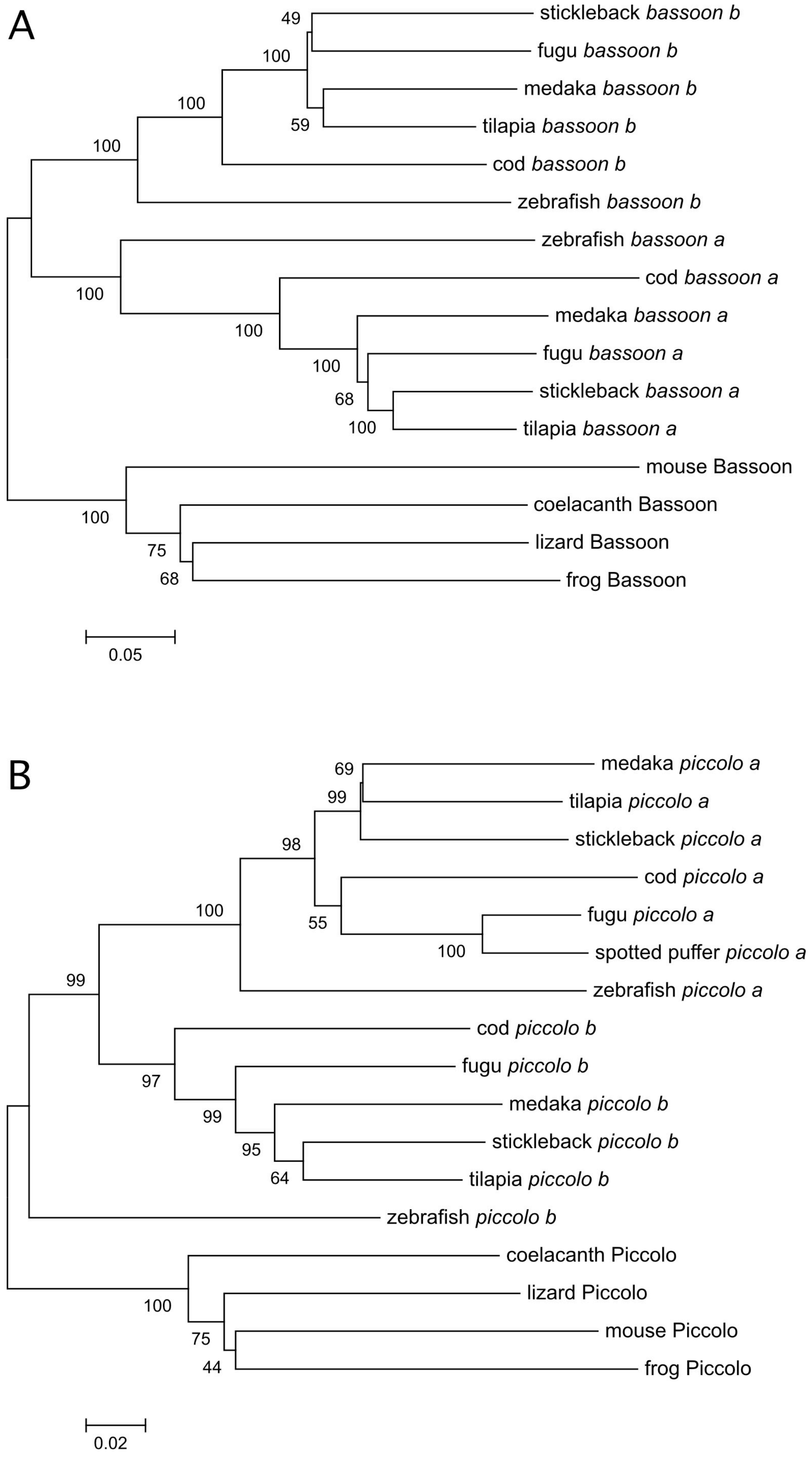
A
stickleback *bassoon b*
fugu *bassoon b*
medaka *bassoon b*
tilapia *bassoon b*
cod *bassoon b*
zebrafish *bassoon b*
zebrafish *bassoon a*
cod *bassoon a*
medaka *bassoon a*
fugu *bassoon a*
stickleback *bassoon a*
tilapia *bassoon a*
mouse Bassoon
coelacanth Bassoon
lizard Bassoon
frog Bassoon
49
100
100
59
100
100
100
100
68
100
100
75
68
0.05
B
medaka *piccolo a*
tilapia *piccolo a*
stickleback *piccolo a*
cod *piccolo a*
fugu *piccolo a*
spotted puffer *piccolo a*
zebrafish *piccolo a*
cod *piccolo b*
fugu *piccolo b*
medaka *piccolo b*
stickleback *piccolo b*
tilapia *piccolo b*
zebrafish *piccolo b*
coelacanth Piccolo
lizard Piccolo
mouse Piccolo
frog Piccolo
69
99
98
100
55
100
99
97
99
95
64
100
75
44
0.02

Figure S8 Additional Evolutionary trees derived from *bassoon* and *piccolo* genes. Evolutionary history was inferred from sequences using a neighbor-joining method. Numbers adjacent to the internal branches indicate bootstrap values. The trees are drawn to scale, with the branch length unit as base substitutions/site. A) The *bassoon* tree was obtained from the entire Bassoon sequence including the zinc finger exons. B) The *piccolo* tree obtained including only the PDZ, C2A, and C2B domains that are not found in Bassoon proteins.
