# Supplementary material for: A Window into Domain Amplification Through Piccolo in Teleost Fish
Source: G3 (Bethesda). 2012 Nov 1;2(11):1325–39. doi: 10.1534/g3.112.003624 (PMC3484663; doi:10.1534/g3.112.003624)
Supplement: Supporting Information [file supp_2.11.1325_FigureS9.pdf]

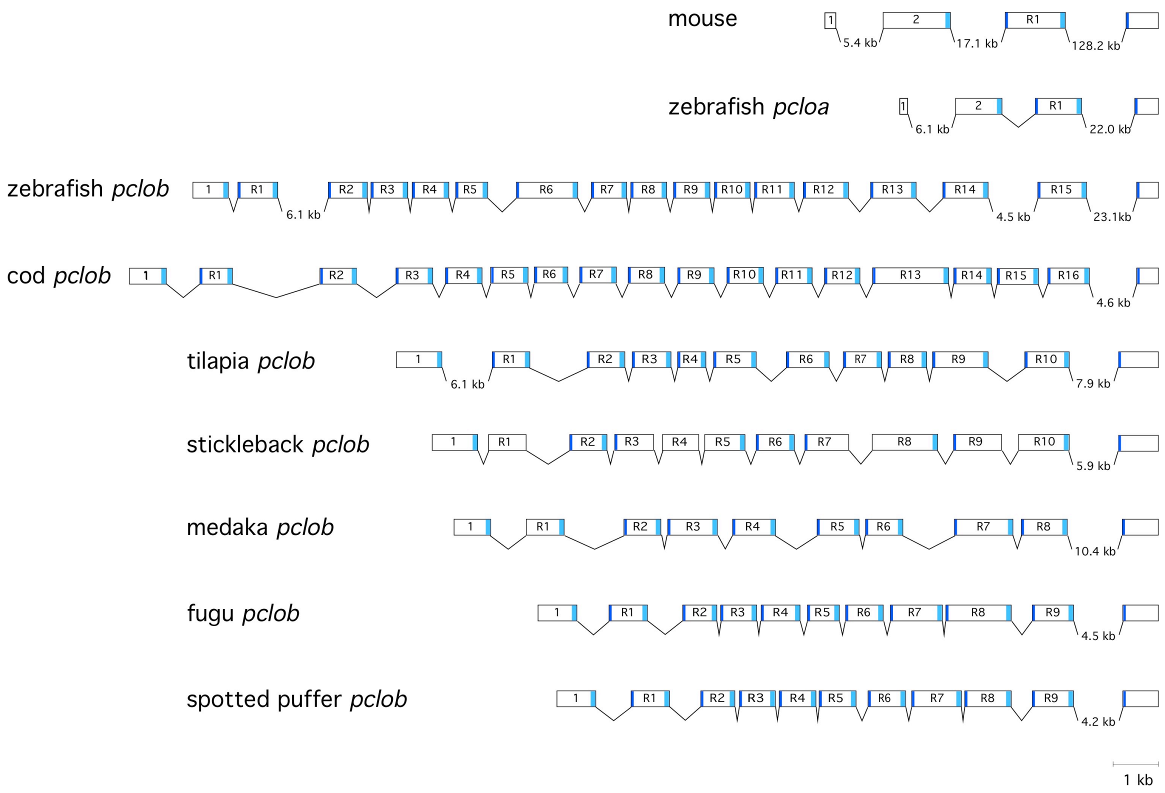

Figure S9 Intron-exon organization of the zinc finger exons from *piccolo* genes. Gene structure of the 5' portion of *piccolo* genes encoding the zinc finger repeats. Light blue and dark blue portions of exon mark the three CxxC and single CxxC components of the domain located on the '3 and 5' ends of the repeating exons. The genomic regions are shown to scale except for large introns, whose sizes are listed. Scale bar on bottom right is 1 kb.
