# Supplementary material for: A Window into Domain Amplification Through Piccolo in Teleost Fish
Source: G3 (Bethesda). 2012 Nov 1;2(11):1325–39. doi: 10.1534/g3.112.003624 (PMC3484663; doi:10.1534/g3.112.003624)
Supplement: Supporting Information [file supp_2.11.1325_FigureS10.pdf]

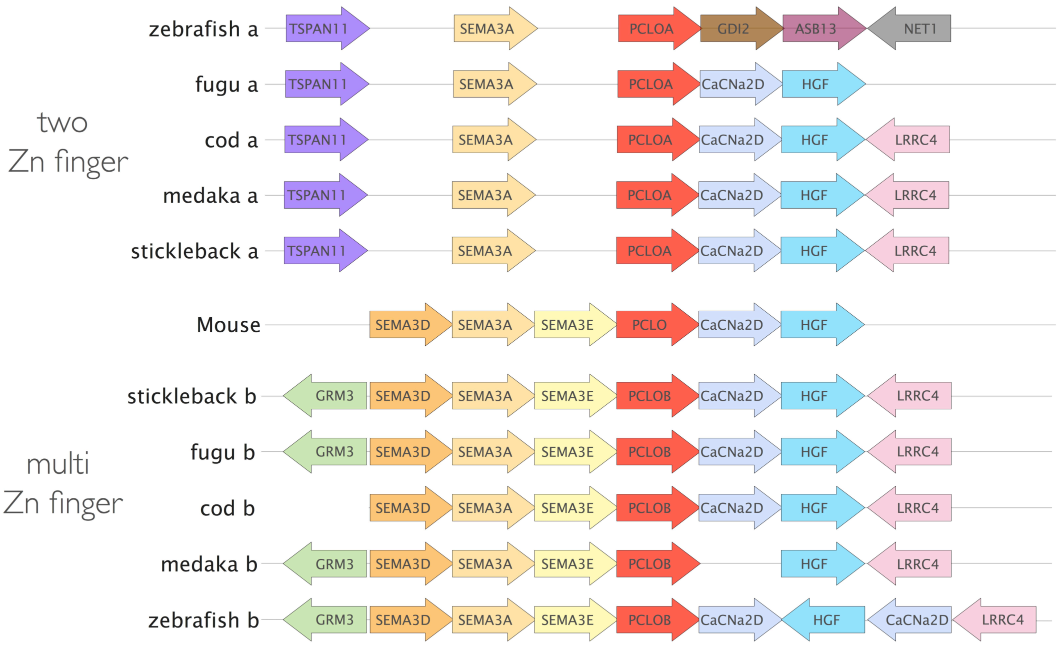

Figure S10 Synteny between the teleost *pcloa*, *pclob* and mouse Piccolo genes. Gene organization near *pcloa* and *pclob* homologs from teleosts. Genes are not shown to scale and certain small loci such as tRNA genes were omitted from the diagrams for simplicity.
