# Supplementary material for: A Window into Domain Amplification Through Piccolo in Teleost Fish
Source: G3 (Bethesda). 2012 Nov 1;2(11):1325–39. doi: 10.1534/g3.112.003624 (PMC3484663; doi:10.1534/g3.112.003624)
Supplement: Supporting Information [file supp_2.11.1325_FigureS12.pdf]

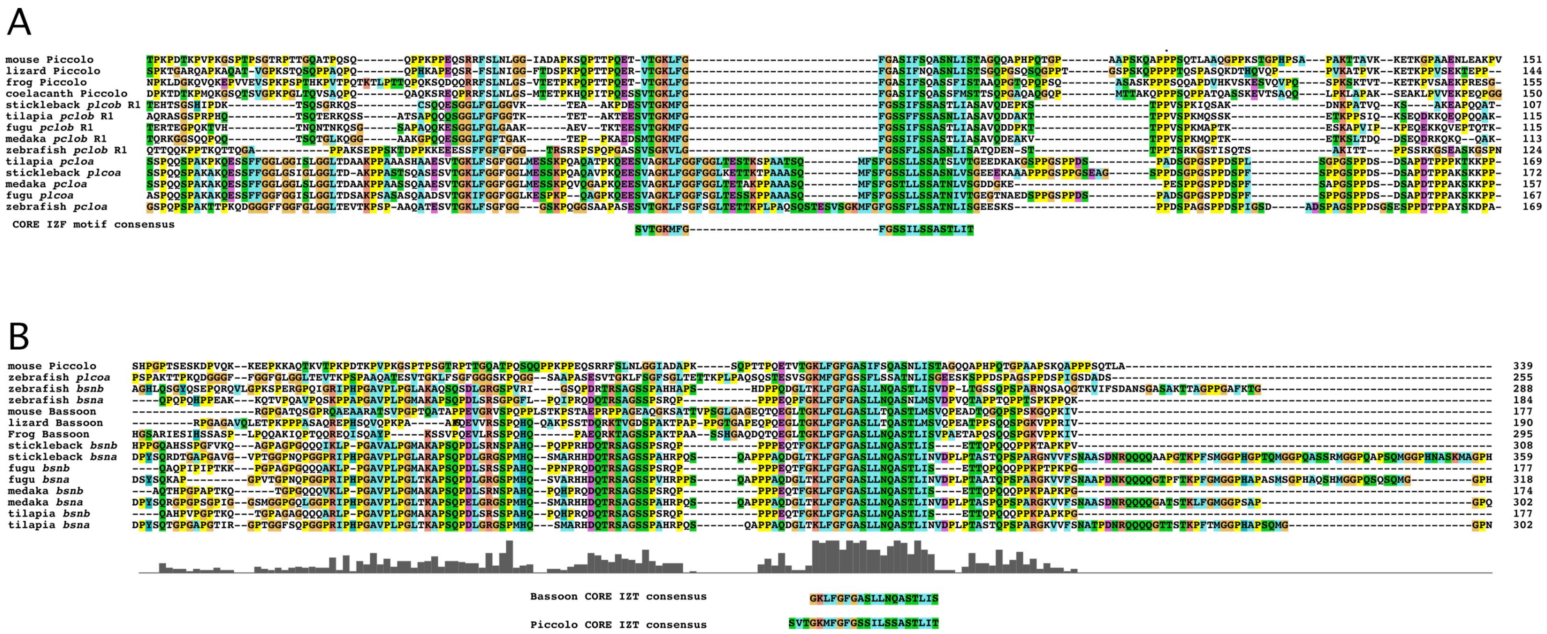

Figure S12 Alignments of the Core IZF domain from teleost *piccolo* and *bassoon* genes. A) An alternative alignment of the Core IZF domain obtained using Clustal W to align the domains illustrating that the IZF domain appears to be have been interrupted by an insertion in the teleost *pcloa* IZF domain. Compare this alignment to the alignment in Figure S9 obtained using MUSCLE. Formatting of the alignment figure was performed in Clustal X as described in methods. B) Alignment of *bassoon* gene zinc finger exons demonstrating that the core IZF domain is largely conserved in the *bassoon* homologs. The alignment was obtained using MUSCLE and formatting of the alignment figure was performed in Clustal X as described in methods.
