# Supplementary material for: A Window into Domain Amplification Through Piccolo in Teleost Fish
Source: G3 (Bethesda). 2012 Nov 1;2(11):1325–39. doi: 10.1534/g3.112.003624 (PMC3484663; doi:10.1534/g3.112.003624)
Supplement: Supporting Information [file supp_2.11.1325_FigureS13.pdf]

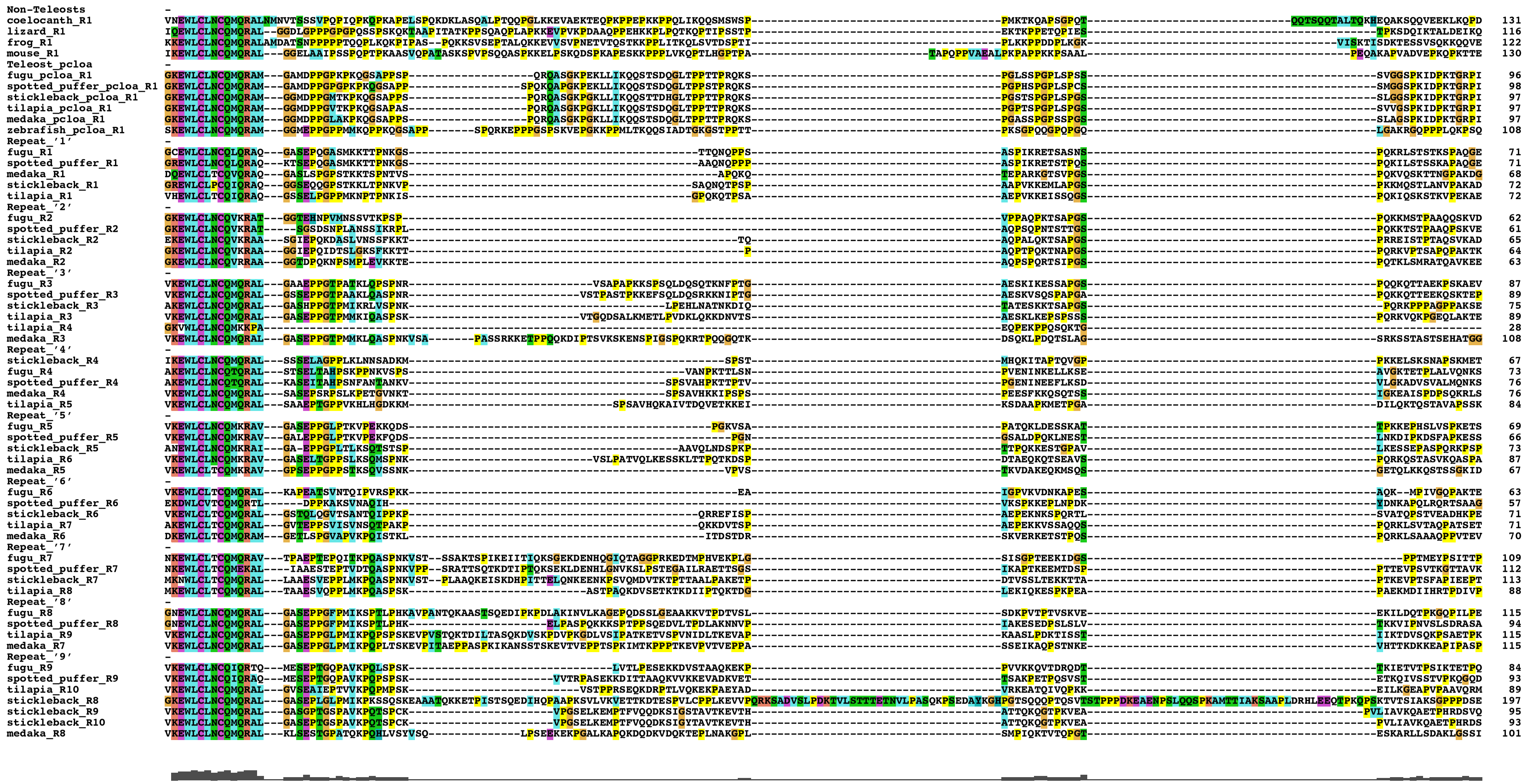
Non-Teleosts
coelocanth_R1 131
lizard_R1 116
frog_R1 122
mouse_R1 130
Teleost_pcloa
fugu_pcloa_R1 96
spotted_puffer_pcloa_R1 98
stickleback_pcloa_R1 97
tilapia_pcloa_R1 97
medaka_pcloa_R1 97
zebrafish_pcloa_R1 108
Repeat_'1'
fugu_R1 71
spotted_puffer_R1 71
medaka_R1 68
stickleback_R1 72
tilapia_R1 72
Repeat_'2'
fugu_R2 62
spotted_puffer_R2 61
stickleback_R2 65
tilapia_R2 64
medaka_R2 63
Repeat_'3'
fugu_R3 87
spotted_puffer_R3 89
stickleback_R3 75
tilapia_R3 89
tilapia_R4 28
medaka_R3 108
Repeat_'4'
stickleback_R4 67
fugu_R4 73
spotted_puffer_R4 76
medaka_R4 76
tilapia_R5 84
Repeat_'5'
fugu_R5 69
spotted_puffer_R5 66
stickleback_R5 73
tilapia_R6 87
medaka_R5 67
Repeat_'6'
fugu_R6 63
spotted_puffer_R6 57
stickleback_R6 71
tilapia_R7 71
medaka_R6 70
Repeat_'7'
fugu_R7 109
spotted_puffer_R7 112
stickleback_R7 113
tilapia_R8 88
Repeat_'8'
fugu_R8 115
spotted_puffer_R8 94
tilapia_R9 115
medaka_R7 115
Repeat_'9'
fugu_R9 84
spotted_puffer_R9 93
tilapia_R10 89
stickleback_R8 197
stickleback_R9 95
stickleback_R10 93
medaka_R8 101

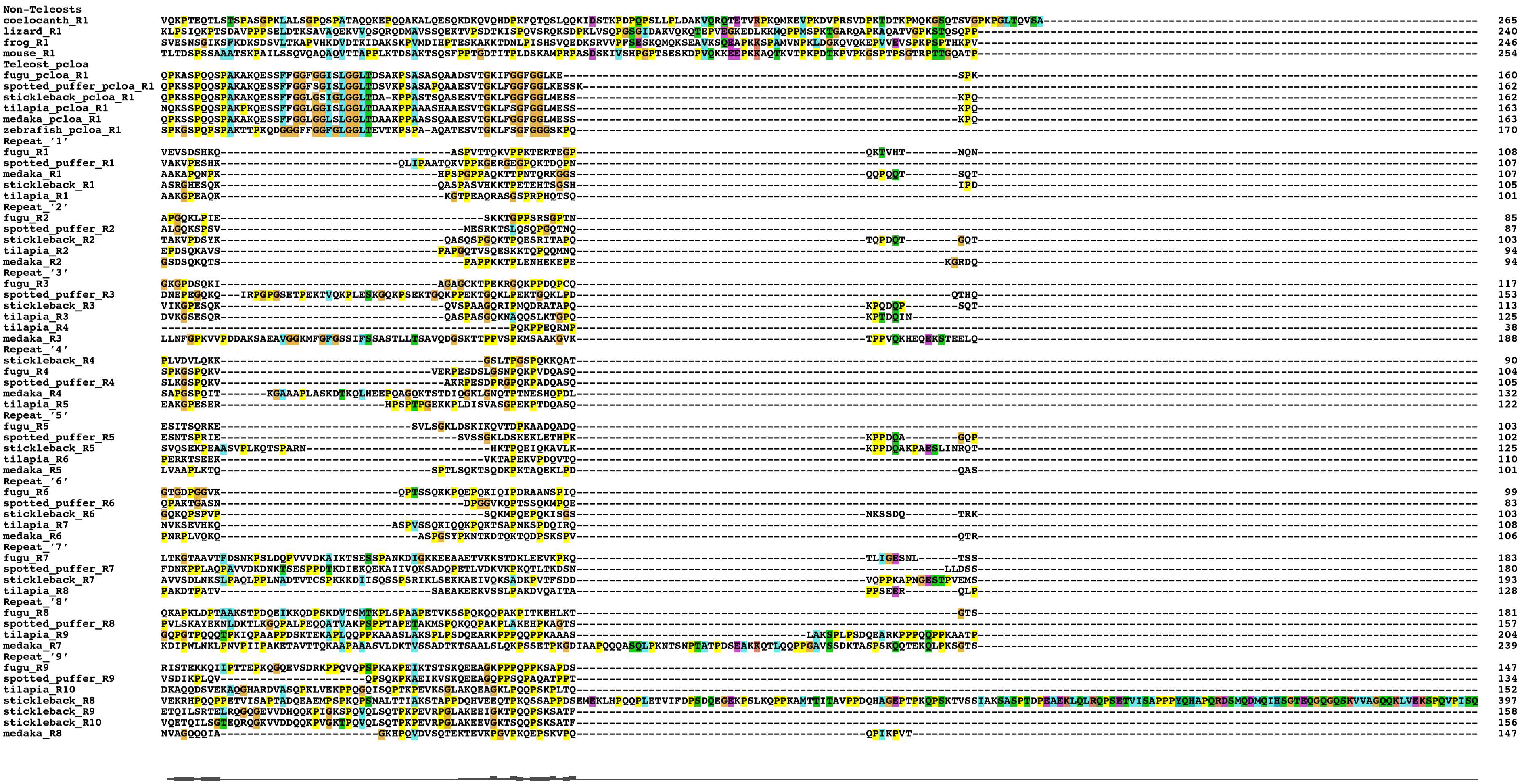
Non-Teleosts
coelocanth_R1 265
lizard_R1 240
frog_R1 246
mouse_R1 254
Teleost_pcloa
fugu_pcloa_R1 160
spotted_puffer_pcloa_R1 162
stickleback_pcloa_R1 162
tilapia_pcloa_R1 163
medaka_pcloa_R1 163
zebrafish_pcloa_R1 170
Repeat_'1'
fugu_R1 108
spotted_puffer_R1 107
medaka_R1 107
stickleback_R1 105
tilapia_R1 101
Repeat_'2'
fugu_R2 85
spotted_puffer_R2 87
stickleback_R2 103
tilapia_R2 94
medaka_R2 94
Repeat_'3'
fugu_R3 117
spotted_puffer_R3 153
stickleback_R3 113
tilapia_R3 125
tilapia_R4 38
medaka_R3 188
Repeat_'4'
stickleback_R4 90
fugu_R4 104
spotted_puffer_R4 105
medaka_R4 132
tilapia_R5 122
Repeat_'5'
fugu_R5 103
spotted_puffer_R5 102
stickleback_R5 125
tilapia_R6 110
medaka_R5 101
Repeat_'6'
fugu_R6 99
spotted_puffer_R6 83
stickleback_R6 103
tilapia_R7 108
medaka_R6 106
Repeat_'7'
fugu_R7 183
spotted_puffer_R7 180
stickleback_R7 193
tilapia_R8 128
Repeat_'8'
fugu_R8 181
spotted_puffer_R8 157
tilapia_R9 204
medaka_R7 239
Repeat_'9'
fugu_R9 147
spotted_puffer_R9 134
tilapia_R10 152
stickleback_R8 397
stickleback_R9 158
stickleback_R10 156
medaka_R8 147

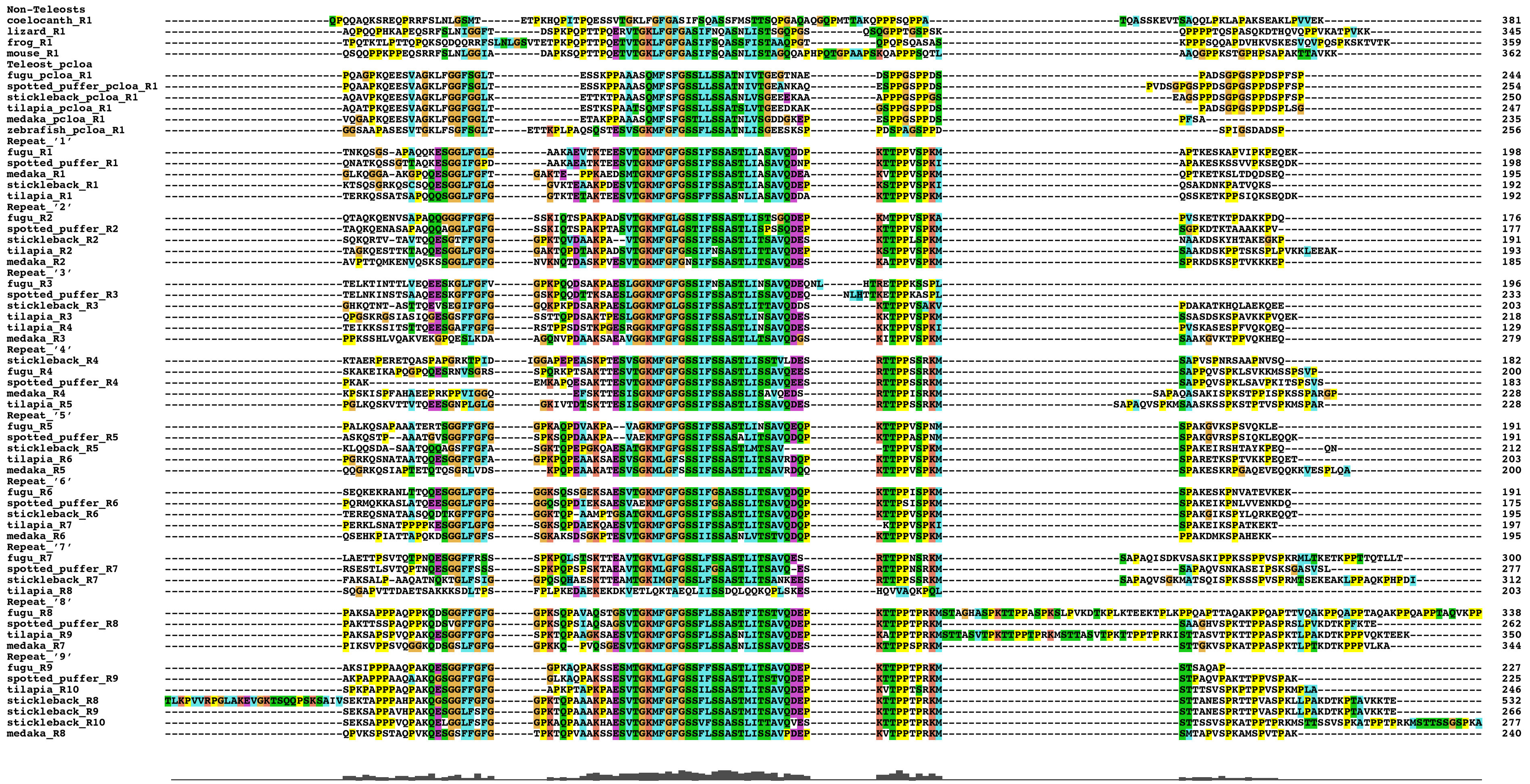

Non-Teleosts
coelocanth_R1
lizard_R1
frog_R1
mouse_R1
Teleost_pcloa
fugu_pcloa_R1
spotted_puffer_pcloa_R1
stickleback_pcloa_R1
tilapia_pcloa_R1
medaka_pcloa_R1
zebrafish_pcloa_R1
Repeat_'1'
fugu_R1
spotted_puffer_R1
medaka_R1
stickleback_R1
tilapia_R1
Repeat_'2'
fugu_R2
spotted_puffer_R2
stickleback_R2
tilapia_R2
medaka_R2
Repeat_'3'
fugu_R3
spotted_puffer_R3
stickleback_R3
tilapia_R3
tilapia_R4
medaka_R3
Repeat_'4'
stickleback_R4
fugu_R4
spotted_puffer_R4
medaka_R4
tilapia_R5
Repeat_'5'
fugu_R5
spotted_puffer_R5
stickleback_R5
tilapia_R6
medaka_R5
Repeat_'6'
fugu_R6
spotted_puffer_R6
stickleback_R6
tilapia_R7
medaka_R6
Repeat_'7'
fugu_R7
spotted_puffer_R7
stickleback_R7
tilapia_R8
Repeat_'8'
fugu_R8
spotted_puffer_R8
tilapia_R9
medaka_R7
Repeat_'9'
fugu_R9
spotted_puffer_R9
tilapia_R10
stickleback_R8
stickleback_R9
stickleback_R10
medaka_R8

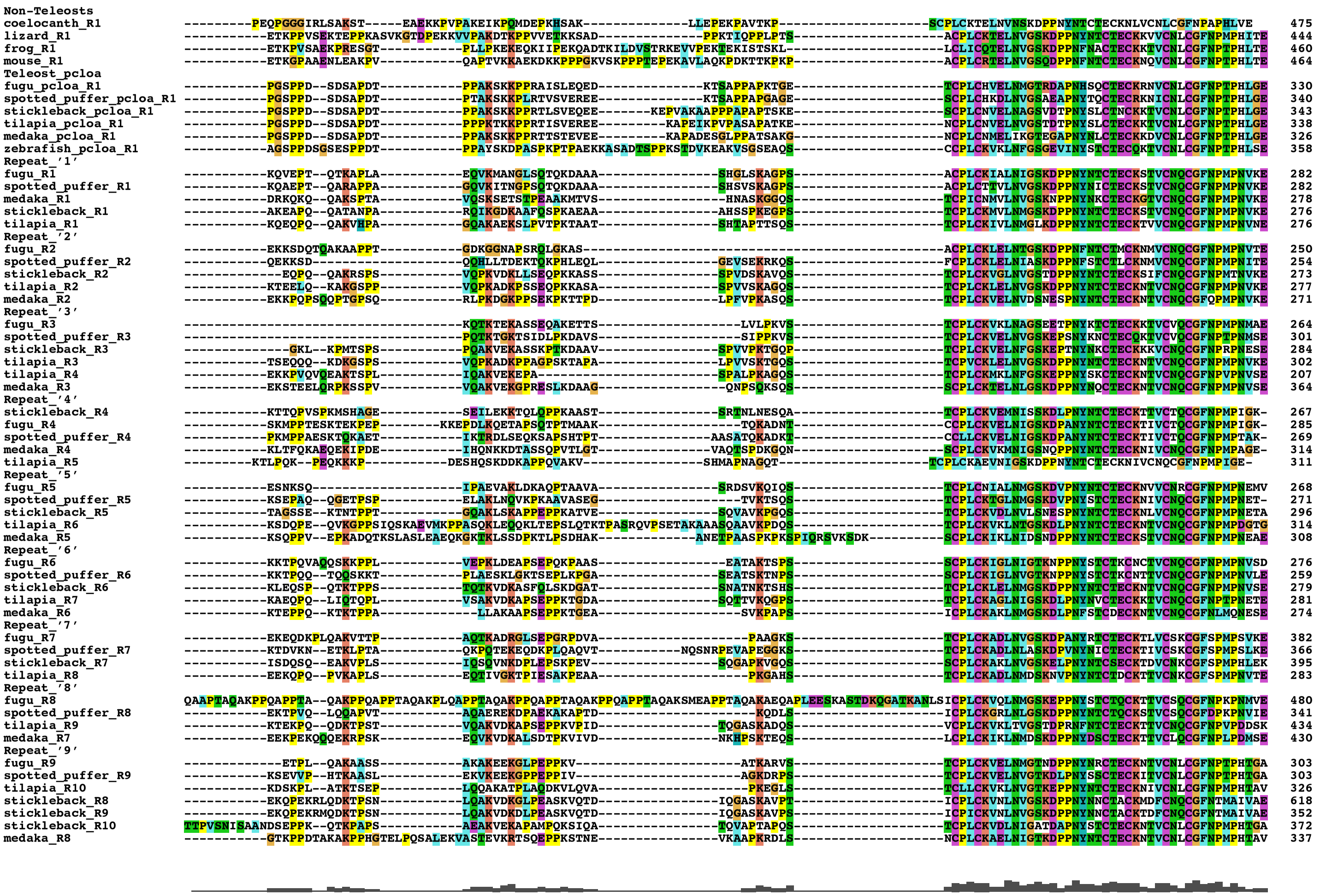
Non-Teleosts
coelocanth_R1 475
lizard_R1 444
frog_R1 460
mouse_R1 464
Teleost_pcloa
fugu_pcloa_R1 330
spotted_puffer_pcloa_R1 340
stickleback_pcloa_R1 343
tilapia_pcloa_R1 338
medaka_pcloa_R1 326
zebrafish_pcloa_R1 358
Repeat_'1'
fugu_R1 282
spotted_puffer_R1 282
medaka_R1 278
stickleback_R1 276
tilapia_R1 276
Repeat_'2'
fugu_R2 250
spotted_puffer_R2 254
stickleback_R2 273
tilapia_R2 277
medaka_R2 271
Repeat_'3'
fugu_R3 264
spotted_puffer_R3 301
stickleback_R3 284
tilapia_R3 302
tilapia_R4 207
medaka_R3 364
Repeat_'4'
stickleback_R4 267
fugu_R4 285
spotted_puffer_R4 269
medaka_R4 314
tilapia_R5 311
Repeat_'5'
fugu_R5 268
spotted_puffer_R5 271
stickleback_R5 296
tilapia_R6 314
medaka_R5 308
Repeat_'6'
fugu_R6 276
spotted_puffer_R6 259
stickleback_R6 279
tilapia_R7 281
medaka_R6 274
Repeat_'7'
fugu_R7 382
spotted_puffer_R7 366
stickleback_R7 395
tilapia_R8 283
Repeat_'8'
fugu_R8 480
spotted_puffer_R8 341
tilapia_R9 434
medaka_R7 430
Repeat_'9'
fugu_R9 303
spotted_puffer_R9 303
tilapia_R10 326
stickleback_R8 618
stickleback_R9 352
stickleback_R10 372
medaka_R8 337
