# Supplementary material for: A Window into Domain Amplification Through Piccolo in Teleost Fish
Source: G3 (Bethesda). 2012 Nov 1;2(11):1325–39. doi: 10.1534/g3.112.003624 (PMC3484663; doi:10.1534/g3.112.003624)
Supplement: Supporting Information [file supp_2.11.1325_FigureS14.pdf]

***: ** .:

Sequence	Segment 1	Segment 2	Segment 3	Segment 4	Length
coelacanth_R1	VNEWLCLNCQMQRALNMNVTSSSVPQPIQPKQPKAPELSPQKDKLASQALPTQQPGLKKEVA	EKTEQPKPPEPKKPPQLIKQQSMSWSPPMKTKQAPS	GPQTQQTSQQTALTQKHEQAK		119
zebrafishPic1_R1	SKEWLCLNCQMQRAMGGMEPPGPPMMKQPP	KQGSAPPSPQRKEPPPGSPSKVEPGKKPPMLTKQQS			66
fugu_pcloa_R1	GKEWLCLNCQMQRAMGAMDPPGPKP	KQGSAPPSPQRQA	SGKPEKLLIKQQS		51
stickleback_pcloa_R1	GKEWLCLNCQMQRAMGGMDPPGMTKP	KQGSAPPSPQRQA	SGKPGKLLIKQQS		52
spotted_puffer_pcloa_R1	GKEWLCLNCQMQRAMGAMDPPGPGPKP	KQGSAPPSPQKQA	PGKPEKLLIKQQS		53
medaka_pcloa_R1	GKEWLCLNCQMQRAMGGMDPPGLAKP	KQGSAPPSPQRQA	SGKPGKLLIKQQS		52
tilapia_pcloa_R1	GKEWLCLNCQMQRAMGGMDPPGVTKP	KQGSAPASPQRQA	SGKPGKLLIKQQS		52
lizard_R1	IQEWLCLNCQMQRALGGDLGPPPGPGPQSSPSKQKTAAPITATKPPSQAQPLAPKKEVP	VKPDAAQPPEHKKPLPQTKQPTIPSSTPEKTKPPETQPIESTPKSDQIKTALDEIKQKLPSIQKPTSDAVPP			131
frog_R1	KKEWLCLNCQMQRALAMDATSNPPPPPTQQPLKQKPIPASPQKKSVSEPTALQKKEVVSVPNETVTQSTKKPPLITKQLSVTDSP	TIPLKKPPDDPLKGKVISKTISDKTESSVSQKKQQV			121
mouse_R1	IKEWLCLNCQMQRALGGELAAIPSSPQPTPKAASVQPATASKSPVPSQQASPKKELPSKQDSPKAPESKKPPPLVKQPTLH	GPTPATAPQPPVA	EALPKPAPPKKPS		107
cod_R1	GKEWLCLPCQMKRASGSPEPAVHSKVQPSP	NK	TATTQKLTPSSS	PPTPKQVTSA	54
cod_R2	GKEWLCLDCQVKRASGAMGEAGPPVRKP				28
cod_R3	VKEWLCLTCQMQRALGASEAPGQLAVKP	QASPKKVSPSP	EPQKKVISES		49
cod_R4	VKEWLCLNCQMQRALGATEPPGTPMMKP	AGSSNKVPSSA	DQLKNLQINS		49
cod_R5	VKEWLCLTCQMQRASNAAALP				21
cod_R6	-KEWLCLNCQMHRALGASEPPGTPFMKP	PPSPSKLPAAPDT	QNKDIPKPHKPQE		53
cod_R7	VKEWLCLNCQMQRALGASEAPGQLAVKP	QASPKKVSPSP	EPQKKVISDS		49
cod_R8	VKEWLCLTCQMQRALGASEAPGQLAVKP	QASPKKVSPSP	EPQKKVISES		49
cod_R9	VKQWLCLTCQMQRALQASEAPGQPSVKA	QASLKKVSPSP	EPQKRVISES		49
cod_R10	VKEWLCLTCQMQRALQASEAQGQPLVKP	QASPKKVSPSP	EPQKKVISES		49
cod_R11	MKEWLCLTCQMQRALRASEAPGQPLVIS	QASPKKVSPSP	EHQKKVISES		49
cod_R12	MKEWLCLTCQMQRALRASEAPGQPLVIP	QASPKKVSPSP	EPQKKVISES		49
cod_R13	VKEWLCLTCQMQRALQASEAPGQPLVKP	QASLKNVPQSP	VPQKKVISES		49
cod_R14	VREWLCLTCQMQRALSASEPEKTPRPQPQASQNKVLESEQCVTPVSSQTKDTPTPGIIDKVEEKPTHVLPQATETSHPGLFKVKEAPGPAPSHIKETSSPALSQDKDITSALPPQARVTSTSGPPQATEISSPALPKATEI	PSPSPPQARETPS			154
cod_R15	MKEWLCLNCQMQRALGVSEPPGLPMIKSQPSS	VQMTNKPAPSPMD	QGAVPITTEDRTP		58
cod_R16	IKEWLCLNCQMQKALGASDPTVPPMMKSPSQIPSHAV	LQEKDKQLGKHEIPVAGAQLPTLTKKDDKLIPESSE			73
zebrafish_R1	AKEWLCLNCQMQRALGGAESSGPPTLKAQ	SQPKDNVPSQNLA	KKDTPHSKQMPPS		55
zebrafish_R2	AKEWLCLNCQTQRALKGIEPPGQPT	IKSKTQPSKSNKM	DSTLGPLEKQSST		51
zebrafish_R3	LKEWLCLNCQTQRALKGMEPPGPPKLES	AAQAKKQPASKPL			41
zebrafish_R4	VKEWLCLSCQIKRASEPSSLDPKLQSKLG	LQQDKRVQHQPPP			42
zebrafish_R5	ANEWLCLTCQVQRASVPKDQAKITTPVP	KKEKDLPESLQKK			41
zebrafish_R6	-VMWLCLACQMQKAPEPPPVQLQSHAKKPEDTVQMDKKPQVPGKQDYKQTSAATVQPAQKETQKQHITDVSK	SESQKTDPQQDKVGFFGFGFGGARSRSPSPQPAVSE			107
zebrafish_R7	KKEWLCLNCQMQRAQAEANKVAPP	VSPLKKTTPAPVP			37
zebrafish_R8	MKEWLCLNCQIQRAPIPPAGQPGP	QTTKLPPPASPQK			37
zebrafish_R9	VKEWLCLTCQMHKTSGPPLPQPEP	QSNIALAPASPLK			37
zebrafish_R10	MKEWLCLTCQVERASGPPPAQQQVPLA				27
zebrafish_R11	VKEWLCLNCQVKKAQEFPSAQPPPMASKASQP	TKSQDMDSPIPSK			45
zebrafish_R12	FNEWLCLNCQTQRALNGIKPQEPSKIKTH	EQPDKVFTPAL	PQKSESITPGSSS		53
zebrafish_R13	VEEWLCLNCQVQRAQQGVKSPSTLPQSV	PLLKDDKTTSKAD	KEAPAPVDTKPDE		54
zebrafish_R14	VDEWLCLNCQVQRAQGGIKSPPVNSQ	TVPLVKDNQTTSK	VDKVAPVESKPDE		52
zebrafish_R15	VDEWLCLNCQVQRAQGGIKSPTVKPQTV	PLVKDNQTTSKAD	KVAPAPVKSKLDE		54
fugu_R1	GCEWLCLNCQLQRAQGASEPQGASMKK	TTPNKGSTTQNQP	PSA-SPIKRETSA		52
fugu_R2	GKEWLCLNCQVKRATGGTEHNPVMNSSV	TKPSPVPPAQPKT			41
fugu_R3	VKEWLCLNCQMQRALGAAEPPGTPATKL	QPSPNRVSAPAPK	KSPSQLDQSQTKN		54
fugu_R4	AKEWLCLNCQTQRALSTSELTAHPS	KPPNKVSPSVANP	KTTLSNPVENINK		51
fugu_R5	VKEWLCLNCQMKRAVGASEPPGLPTKVPE	KKQDSPGKVSAPA			42
fugu_R6	VKEWLCLTCQMQRALKAPEA	TSVNTQIPVRSPK	KEAIGPVKVDNKA		46
fugu_R7	NKEWLCLTCQMQRAVTPAEPTEPQITKPQASPNKVSTSSAKTSPIKEIITIQKSGEKDENHQGIQTAGG	PRKEDTMPHVEKPLGSISGPTEEKIDGSPPTMEYPS			105
fugu_R8	GNEWLCLNCQMQRALGASEPPGFPMIKSPTLPHKAVPANTQKAASTSQEDIPKPDLAKINVLKA	GEPQDSSLGEAAKKVTPDTVSLSDKPVTPTVSKVEE			100
fugu_R9	VKEWLCLNCQIQRTQMESEPTGQPAVKP	QLSPSKLVTLPESEKKDVSTAAQKEKPPVVKKQVTD			64
medaka_R1	DQEWLCLTCQVQRAQGASLSPGPSTKK	TSPNTVSAPQKQT	EPARKGTSVPGSP		53
medaka_R2	GKEWLCLNCQVRRAAGGTDPQKNPSMPL	EVKKTEAQPSP	QRTSI		44
medaka_R3	VKEWLCLNCQMQRALGASEPPGTPMMKLQASPNKVSAP	ASSRKKETPPQQKDIPTSVKSKENSPIGSPQKRTPQ			74
medaka_R4	VKEWLCLNCQMQRALSASEPSRPSLKPETGVN	KTSPSAVHKKIPS	PSPEESFKKQSQT		58
medaka_R5	VKEWLCLTCQMKRAVGPSEPPGPPSTKS	QVSSNKVPVSTKV	DAKEQKMSQSGET		54
medaka_R6	DKEWLCLTCQMQRAMGETLSPGVAPVKP	QISTKLITDST	DRSKVERKETST		51
medaka_R7	VKEWLCLNCQMQRALGASEPPGLPMIKPQPLTSKEVPITAEPPASPKIKANSSTSKEVTVEPPTSPKIMTK	PPPTKEVPVTVEP	PASSEIKAQPSTNKEVHTTKDKKEAPIPASPKDIPWLNKLPNVPIIPAK		133
medaka_R8	VKEWLCLNCQMQRALKLSESTGPATQKPQHLVSYVSQLPSEEK	EKPGALKAPQKDQ	DKVDQKTEPLNAK		69
stickleback_R1	GREWLCLPCQIQRAQGGSEQQGPSTKK	LTPNKVPSAQNQT	PSPAAPVKKEMLA		53
stickleback_R2	EKEWLCLNCQVKRAASGIEPQKDASLVN	SSFKKTTQAQPAL	QKTSAPGSPRREI		54
stickleback_R3	AKEWLCLNCQMQRALGASHPPGTPMIKR	LVSPNKLPEHLNA	TNKDIQTATESKK		54
stickleback_R4	IKEWLCLNCQMQRALSSSELAGPPLKL	NNSADKMSPSTMH			40
stickleback_R5	ANEWLCLNCQMKRAIGA-EPPGPLTLKSQTSTSPAAVQ	LNDSPKPTTPQKK	ESTGPAVLKESSE		63
stickleback_R6	VKEWLCLTCQMQRALGSTQLQGV	TSANTQIPPKP	QRREFISPAEPEK		47
stickleback_R7	MKNWLCLTCQMQRALLAAESVEPPLMKPQASPNKVSTPLAAQKEISKDHPITTELQNKEENKPSVQMDV	TKTPTTAALPAKETPDTVSSLTEKKTTAPTKEVPTS			105
stickleback_R8	GKEWLCLNCQMQRALGASEPLGLPMIKPKSSQSKEAAATQKKETPISTSQEDIHQPAAPKSVLVKVETTKDTESPVLCPPLKEVVPQRKSADVSLPDKTVLSTTTETNVLPASQ	KPSEDAYKGHPGTSQQQPTQSVTSTPPPDKEAENPSLQQSPKAMTTIAKSAAPLDRHLEEQTPKQPSKTVTS			186
stickleback_R9	VKEWLCLNCQMQRALGASGPTGSPAVKPQTSPCKVPGSELKEM	PTFVQQDKSIGSTAVTKEVTHATTQKQGTPKVEAPV			79
stickleback_R10	VKEWLCLNCQMQRALGASEPTGSPAVKPQTSPCKVPGSELK	EMPTFVQQDKSIGYTAVTKEVTHATTQKQGTPKVEA			77
spotted_puffer_R1	GREWLCLNCQLQRAQKTSEPQGASMKK	TTPNKGSAAQNQP	PPA-SPIKRETST		52
spotted_puffer_R2	GKEWLCLNCQVKRATSGSDSNPLAN				25
spotted_puffer_R3	VKEWLCLNCQMQRALGSSEPPGTPAAKLQASPNR	VSTPASTPKKEFSQLDQSRKKNIPTGAESKVSQSPA			70
spotted_puffer_R4	AKEWLCLNCQTQRALKASEITAHPSNFA	NTANKVSPSVAHP	KTTPTVPGENINE		54
spotted_puffer_R5	VKEWLCLNCQMKRAVGALEPPGLPTKVPEKFQ	DSPGNGSALDPQK			45
spotted_puffer_R6	EKDWLCVTCQMQRTLDPPKAKSVNAQIH	VKSPKKEPLNPDK	YDNKAPQLRQRTS		54
spotted_puffer_R7	NKEWLCLTCQMEKALIAAESTEPTVDTQASPNKVPPSRATT	SQTKDTIPTQKSEKLDENHLGNVKSLPSTEGAILRA			77
spotted_puffer_R8	GNEWLCLNCQMQRALGASEPPGFPMIKSPTLPHKELP	ASPQKKKSPTPPSQEDVLTPDLAKNNVPIAKESEDP			73
spotted_puffer_R9	VKEWLCLNCQIQRAQMESEPTGQPAVKPQP	SPSKVVTRPASEKKDITTAAQKVVKKEVADKVETTS			66
tilapia_R1	VHEWLCLTCQIQRAQGSSELPGPPMKN	PTPNKISGPQKQT	PSAAEPVKKEISS		53
tilapia_R2	GKEWLCLNCQVKRAAGGIEPQIDTSLG	KSFKKTTPAQPTP	QKTNAPGSPQRKV		53
tilapia_R3	VKEWLCLNCQMQRALGASEPPGTPMMKIQASPSKVTGQDSALKM	ETLPVDKLQKKDN	VTSAESKLKEPSP		70
tilapia_R4	GKVWLCLNCQMKK				13
tilapia_R5	VKEWLCLNCQMQRALSAAEPTGPPVKHLHGDK	KMSPSAVHQKAIV	TDQVETKKEIKSD		58
tilapia_R6	VKEWLCLNCQMKRAVGASELTGPPSLKS	QMSPNKVSLPATV	QLKESSKLTTPQT		54
tilapia_R7	AKEWLCLTCQMQRALGVTEPPSVISVNS	QTPAKPQKKDVTS	PAEPEKKVSSAQQ		54
tilapia_R8	MKEWLCLTCQMQRALTAAESVQPPLMKPQ	ASPSKASTPAQKDVSETKTKDIIPTQKTDGLEKIQK			65
tilapia_R9	VKEWLCLNCQMQRALGASEPPGLPMIKPQPSPSKEVPVSTQKTDILTASQKDVSKPDVPKGDLVSIPATKETVSPVNIDLTKEVAPKAASLPDKTISSTIIKT	DVSQKPSAETPKG	QPGTPQQQTPKIQ		129
tilapia_R10	VKEWLCLNCQMQRALGVSEAIEPTVVKP	QPMPSKVSTPPRSEQKDRPTLVQKEKPAEYADVRKE			64

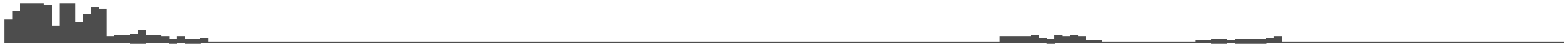

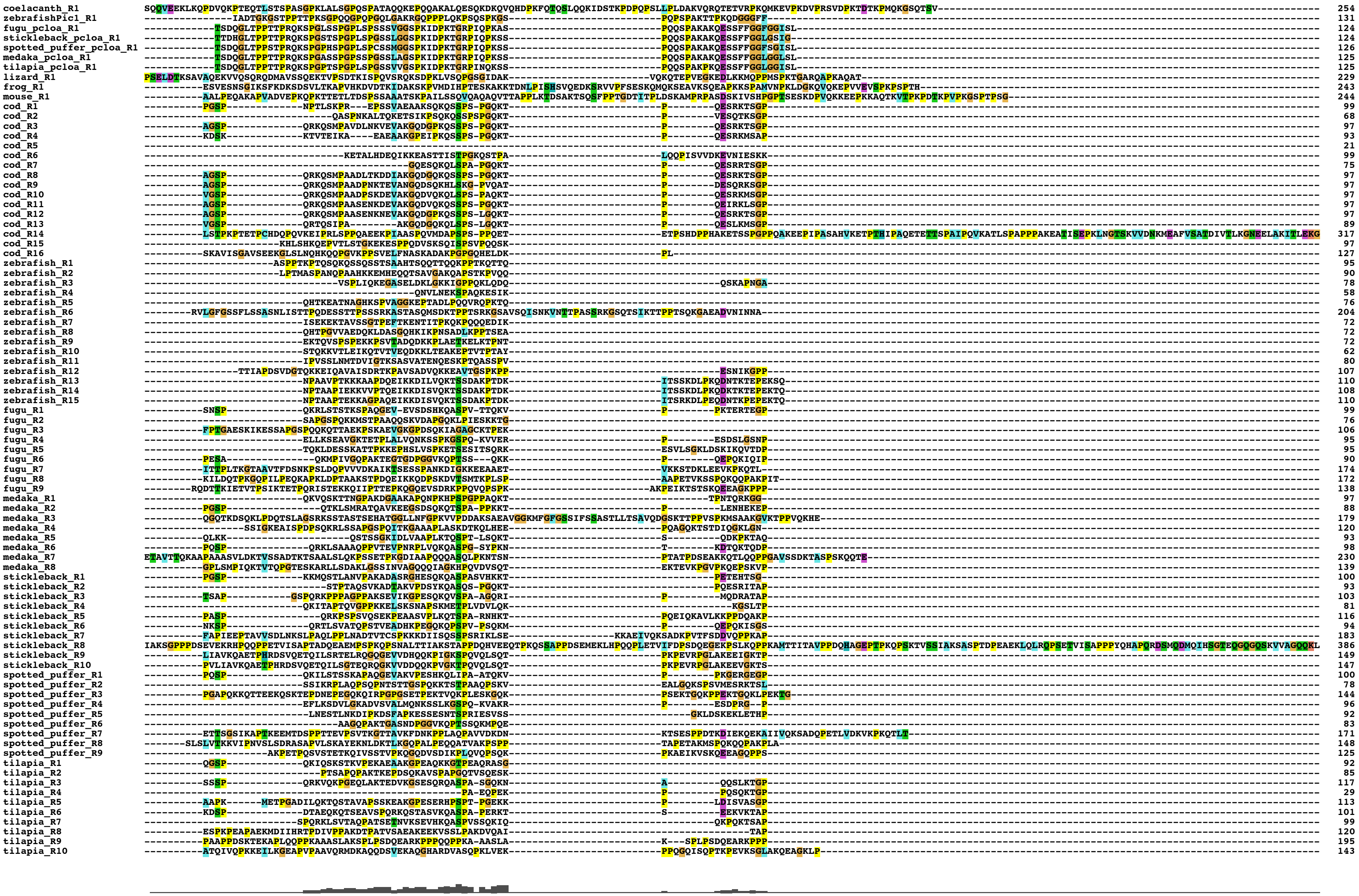
coelacanth_R1 254
zebrafishPlc1_R1 131
fugu_pcloa_R1 124
stickleback_pcloa_R1 124
spotted_puffer_pcloa_R1 126
medaka_pcloa_R1 125
tilapia_pcloa_R1 125
lizard_R1 229
frog_R1 243
mouse_R1 244
cod_R1 99
cod_R2 68
cod_R3 97
cod_R4 93
cod_R5 21
cod_R6 99
cod_R7 75
cod_R8 97
cod_R9 97
cod_R10 97
cod_R11 97
cod_R12 97
cod_R13 89
cod_R14 317
cod_R15 97
cod_R16 127
zebrafish_R1 95
zebrafish_R2 90
zebrafish_R3 78
zebrafish_R4 58
zebrafish_R5 76
zebrafish_R6 204
zebrafish_R7 72
zebrafish_R8 72
zebrafish_R9 72
zebrafish_R10 62
zebrafish_R11 80
zebrafish_R12 107
zebrafish_R13 110
zebrafish_R14 108
zebrafish_R15 110
fugu_R1 99
fugu_R2 76
fugu_R3 106
fugu_R4 95
fugu_R5 95
fugu_R6 90
fugu_R7 174
fugu_R8 172
fugu_R9 138
medaka_R1 97
medaka_R2 88
medaka_R3 179
medaka_R4 120
medaka_R5 93
medaka_R6 98
medaka_R7 230
medaka_R8 139
stickleback_R1 100
stickleback_R2 93
stickleback_R3 103
stickleback_R4 81
stickleback_R5 116
stickleback_R6 94
stickleback_R7 183
stickleback_R8 386
stickleback_R9 149
stickleback_R10 147
spotted_puffer_R1 100
spotted_puffer_R2 78
spotted_puffer_R3 144
spotted_puffer_R4 96
spotted_puffer_R5 92
spotted_puffer_R6 83
spotted_puffer_R7 171
spotted_puffer_R8 148
spotted_puffer_R9 125
tilapia_R1 92
tilapia_R2 85
tilapia_R3 117
tilapia_R4 29
tilapia_R5 113
tilapia_R6 101
tilapia_R7 99
tilapia_R8 120
tilapia_R9 195
tilapia_R10 143

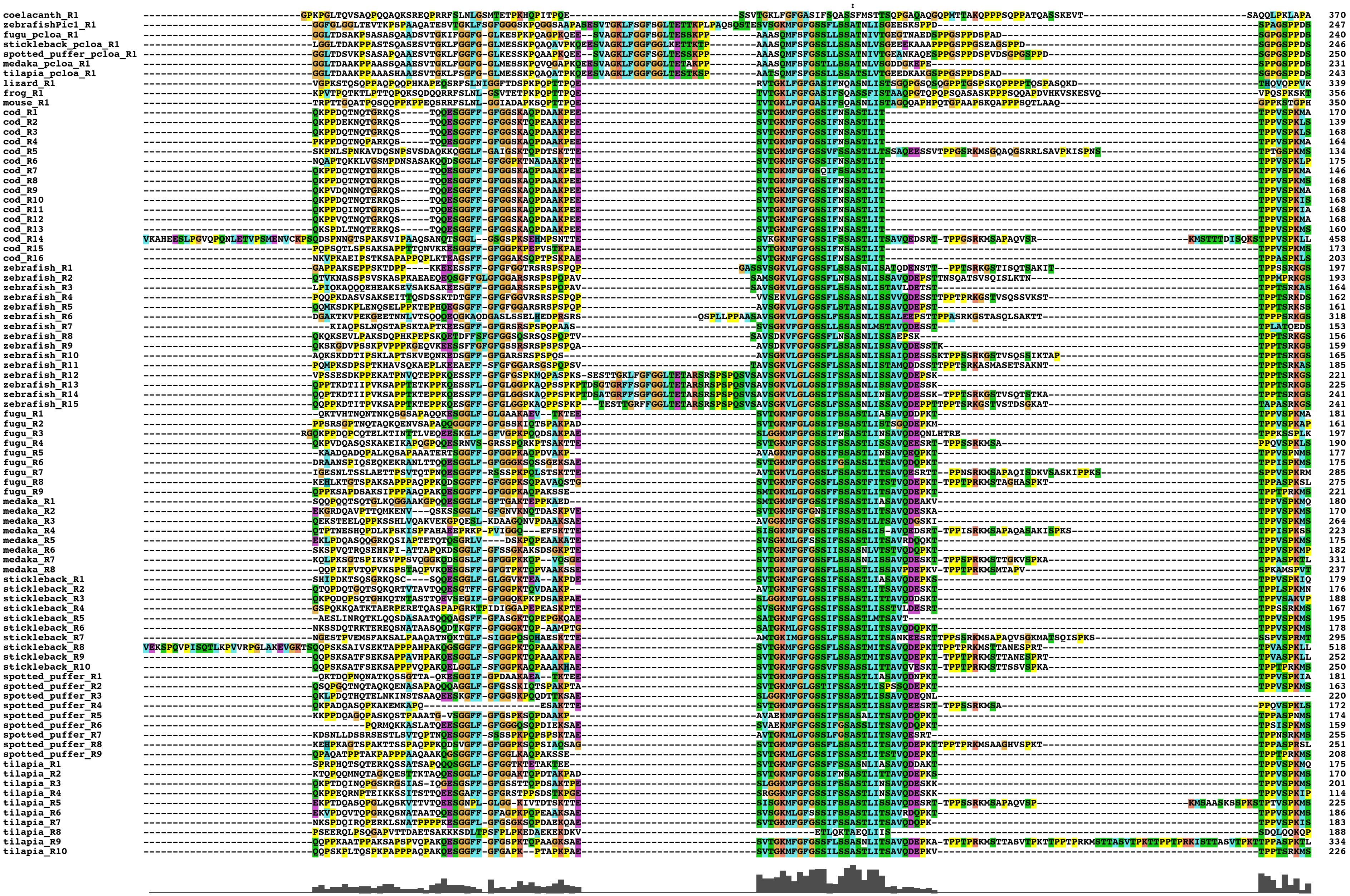
coelacanth_R1
zebrafishPlc1_R1
fugu_pcloa_R1
stickleback_pcloa_R1
spotted_puffer_pcloa_R1
medaka_pcloa_R1
tilapia_pcloa_R1
lizard_R1
frog_R1
mouse_R1
cod_R1
cod_R2
cod_R3
cod_R4
cod_R5
cod_R6
cod_R7
cod_R8
cod_R9
cod_R10
cod_R11
cod_R12
cod_R13
cod_R14
cod_R15
cod_R16
zebrafish_R1
zebrafish_R2
zebrafish_R3
zebrafish_R4
zebrafish_R5
zebrafish_R6
zebrafish_R7
zebrafish_R8
zebrafish_R9
zebrafish_R10
zebrafish_R11
zebrafish_R12
zebrafish_R13
zebrafish_R14
zebrafish_R15
fugu_R1
fugu_R2
fugu_R3
fugu_R4
fugu_R5
fugu_R6
fugu_R7
fugu_R8
fugu_R9
medaka_R1
medaka_R2
medaka_R3
medaka_R4
medaka_R5
medaka_R6
medaka_R7
medaka_R8
stickleback_R1
stickleback_R2
stickleback_R3
stickleback_R4
stickleback_R5
stickleback_R6
stickleback_R7
stickleback_R8
stickleback_R9
stickleback_R10
spotted_puffer_R1
spotted_puffer_R2
spotted_puffer_R3
spotted_puffer_R4
spotted_puffer_R5
spotted_puffer_R6
spotted_puffer_R7
spotted_puffer_R8
spotted_puffer_R9
tilapia_R1
tilapia_R2
tilapia_R3
tilapia_R4
tilapia_R5
tilapia_R6
tilapia_R7
tilapia_R8
tilapia_R9
tilapia_R10

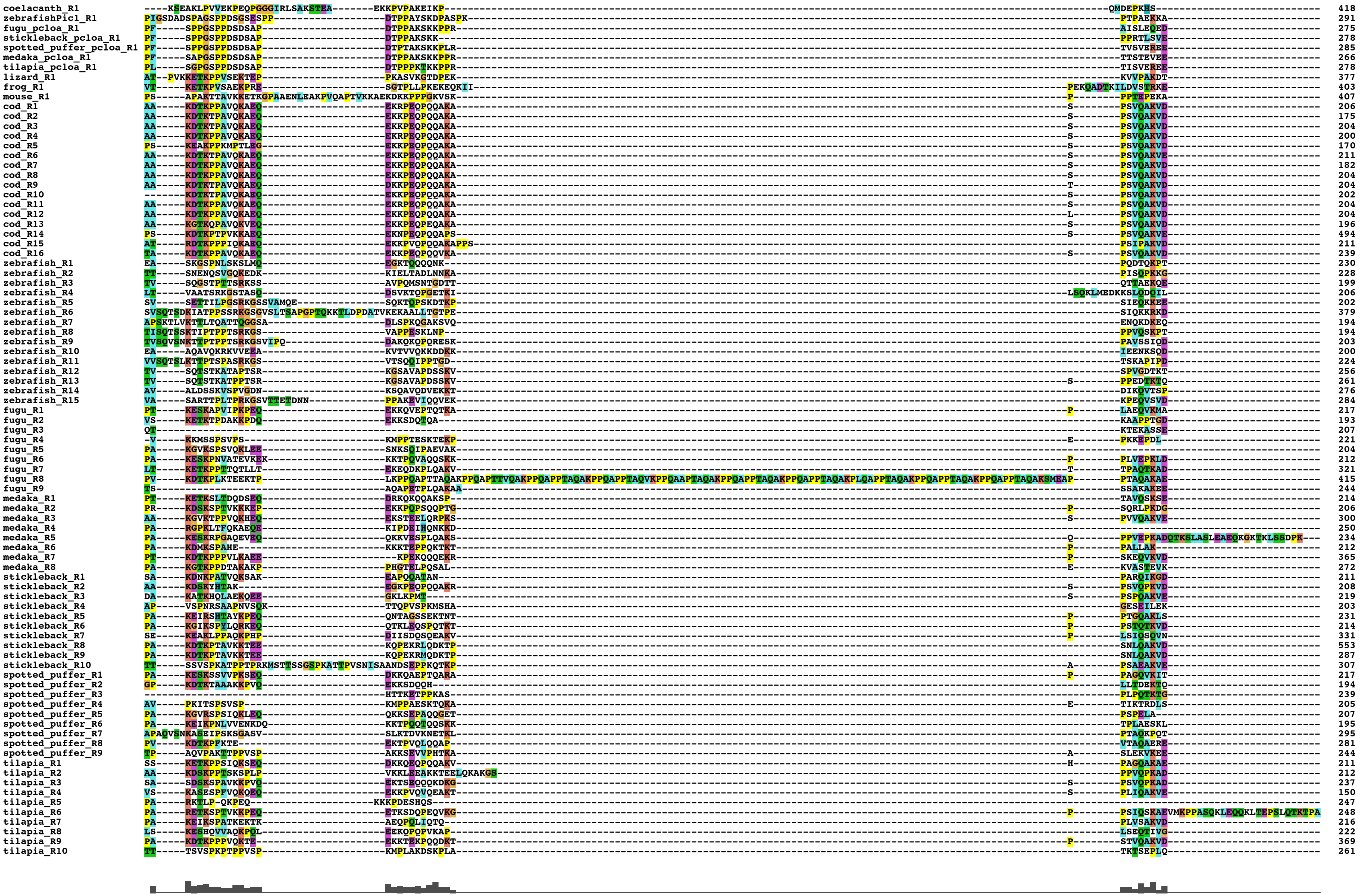

coelacanth_R1 418
zebrafishPlc1_R1 291
fugu_pcloa_R1 275
stickleback_pcloa_R1 278
spotted_puffer_pcloa_R1 285
medaka_pcloa_R1 266
tilapia_pcloa_R1 278
lizard_R1 377
frog_R1 403
mouse_R1 407
cod_R1 206
cod_R2 175
cod_R3 204
cod_R4 200
cod_R5 170
cod_R6 211
cod_R7 182
cod_R8 204
cod_R9 204
cod_R10 202
cod_R11 204
cod_R12 204
cod_R13 196
cod_R14 494
cod_R15 211
cod_R16 239
zebrafish_R1 230
zebrafish_R2 228
zebrafish_R3 199
zebrafish_R4 206
zebrafish_R5 202
zebrafish_R6 379
zebrafish_R7 194
zebrafish_R8 194
zebrafish_R9 203
zebrafish_R10 200
zebrafish_R11 224
zebrafish_R12 256
zebrafish_R13 261
zebrafish_R14 276
zebrafish_R15 284
fugu_R1 217
fugu_R2 193
fugu_R3 207
fugu_R4 221
fugu_R5 204
fugu_R6 212
fugu_R7 321
fugu_R8 415
fugu_R9 244
medaka_R1 214
medaka_R2 206
medaka_R3 300
medaka_R4 250
medaka_R5 234
medaka_R6 212
medaka_R7 365
medaka_R8 272
stickleback_R1 211
stickleback_R2 208
stickleback_R3 219
stickleback_R4 203
stickleback_R5 231
stickleback_R6 214
stickleback_R7 331
stickleback_R8 553
stickleback_R9 287
stickleback_R10 307
spotted_puffer_R1 217
spotted_puffer_R2 194
spotted_puffer_R3 239
spotted_puffer_R4 205
spotted_puffer_R5 207
spotted_puffer_R6 195
spotted_puffer_R7 295
spotted_puffer_R8 281
spotted_puffer_R9 244
tilapia_R1 211
tilapia_R2 212
tilapia_R3 237
tilapia_R4 150
tilapia_R5 247
tilapia_R6 248
tilapia_R7 216
tilapia_R8 222
tilapia_R9 369
tilapia_R10 261

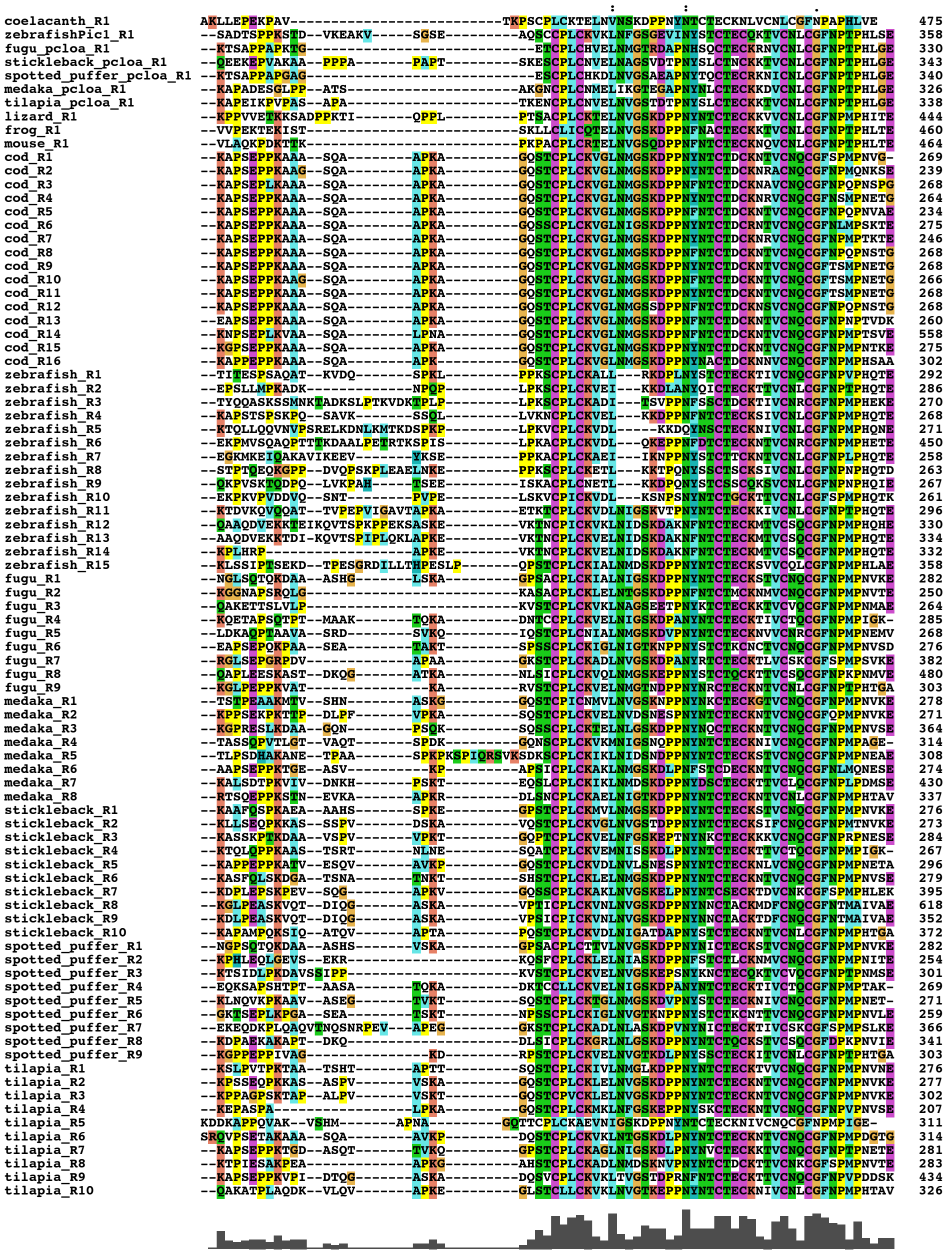
coelacanth_R1 475
zebrafishPlc1_R1 358
fugu_pcloa_R1 330
stickleback_pcloa_R1 343
spotted_puffer_pcloa_R1 340
medaka_pcloa_R1 326
tilapia_pcloa_R1 338
lizard_R1 444
frog_R1 460
mouse_R1 464
cod_R1 269
cod_R2 239
cod_R3 268
cod_R4 264
cod_R5 234
cod_R6 275
cod_R7 246
cod_R8 268
cod_R9 268
cod_R10 266
cod_R11 268
cod_R12 268
cod_R13 260
cod_R14 558
cod_R15 275
cod_R16 302
zebrafish_R1 292
zebrafish_R2 286
zebrafish_R3 270
zebrafish_R4 268
zebrafish_R5 271
zebrafish_R6 450
zebrafish_R7 258
zebrafish_R8 263
zebrafish_R9 267
zebrafish_R10 261
zebrafish_R11 296
zebrafish_R12 330
zebrafish_R13 334
zebrafish_R14 332
zebrafish_R15 358
fugu_R1 282
fugu_R2 250
fugu_R3 264
fugu_R4 285
fugu_R5 268
fugu_R6 276
fugu_R7 382
fugu_R8 480
fugu_R9 303
medaka_R1 278
medaka_R2 271
medaka_R3 364
medaka_R4 314
medaka_R5 308
medaka_R6 274
medaka_R7 430
medaka_R8 337
stickleback_R1 276
stickleback_R2 273
stickleback_R3 284
stickleback_R4 267
stickleback_R5 296
stickleback_R6 279
stickleback_R7 395
stickleback_R8 618
stickleback_R9 352
stickleback_R10 372
spotted_puffer_R1 282
spotted_puffer_R2 254
spotted_puffer_R3 301
spotted_puffer_R4 269
spotted_puffer_R5 271
spotted_puffer_R6 259
spotted_puffer_R7 366
spotted_puffer_R8 341
spotted_puffer_R9 303
tilapia_R1 276
tilapia_R2 277
tilapia_R3 302
tilapia_R4 207
tilapia_R5 311
tilapia_R6 314
tilapia_R7 281
tilapia_R8 283
tilapia_R9 434
tilapia_R10 326
