# Supplementary material for: A Window into Domain Amplification Through Piccolo in Teleost Fish
Source: G3 (Bethesda). 2012 Nov 1;2(11):1325–39. doi: 10.1534/g3.112.003624 (PMC3484663; doi:10.1534/g3.112.003624)
Supplement: Supporting Information [file supp_2.11.1325_FigureS15.pdf]

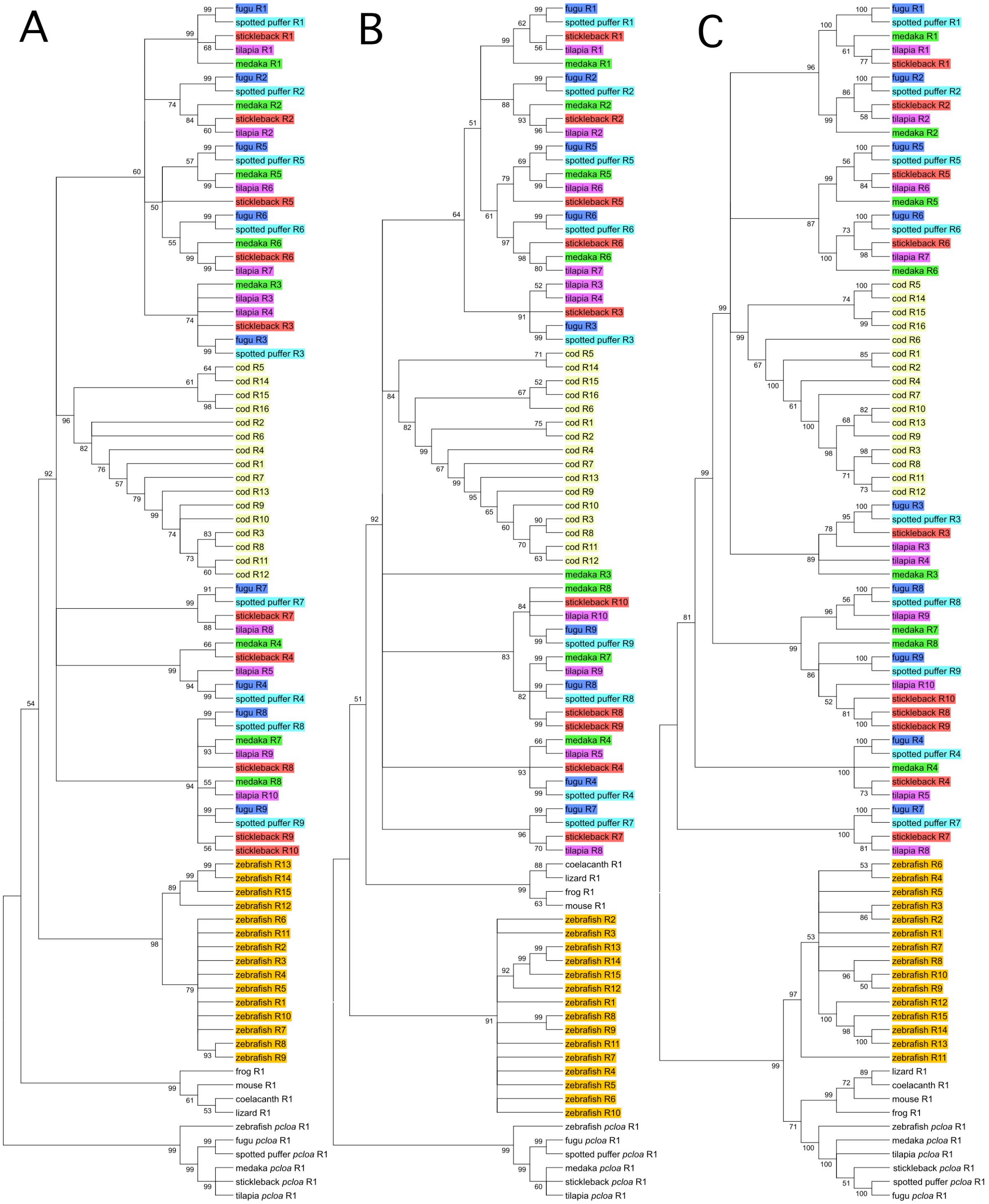

Figure S15 Comparison of evolutionary trees of repeated zinc finger domains obtained using distinct alignment methods and gap penalties. Tree trees constructed from alignments created by A) Clustal W alignment run within MEGA5 using default values for gap opening and extension without a negatively weighted substitution matrix. These are the default parameters for Clustal X stand alone application used in initial analysis of these sequences. B) MUSCLE run within MEGA5 under default parameters (-2.9 gap opening penalty, 0 gap extension penalty), and C) MUSCLE run under parameters used for the zinc finger repeat analysis presented in the rest of the paper (-4.9 gap opening penalty, -0.01 gap extension penalty).
