# Supplementary material for: A Window into Domain Amplification Through Piccolo in Teleost Fish
Source: G3 (Bethesda). 2012 Nov 1;2(11):1325–39. doi: 10.1534/g3.112.003624 (PMC3484663; doi:10.1534/g3.112.003624)
Supplement: Supporting Information [file supp_2.11.1325_FigureS16.pdf]

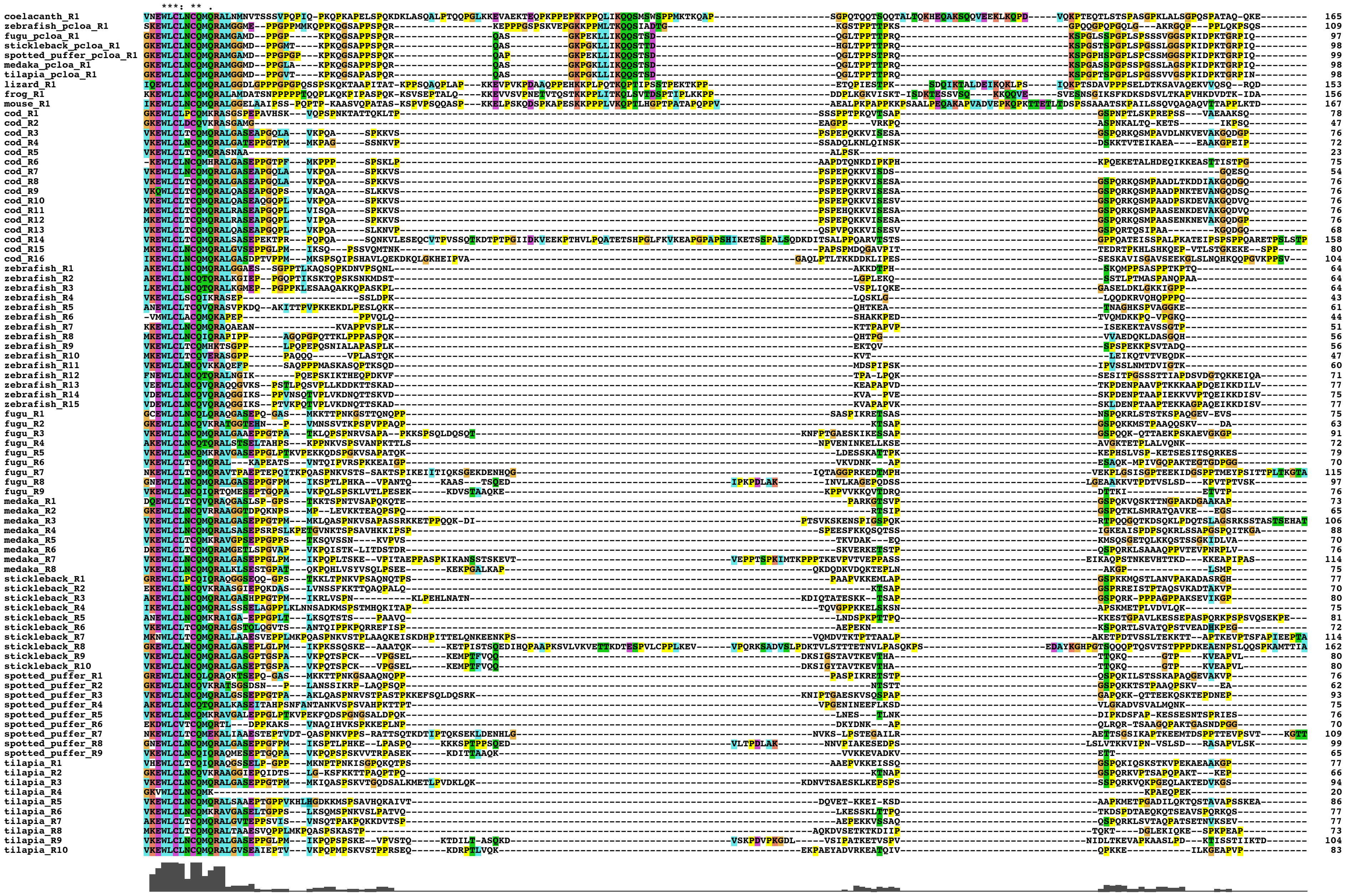

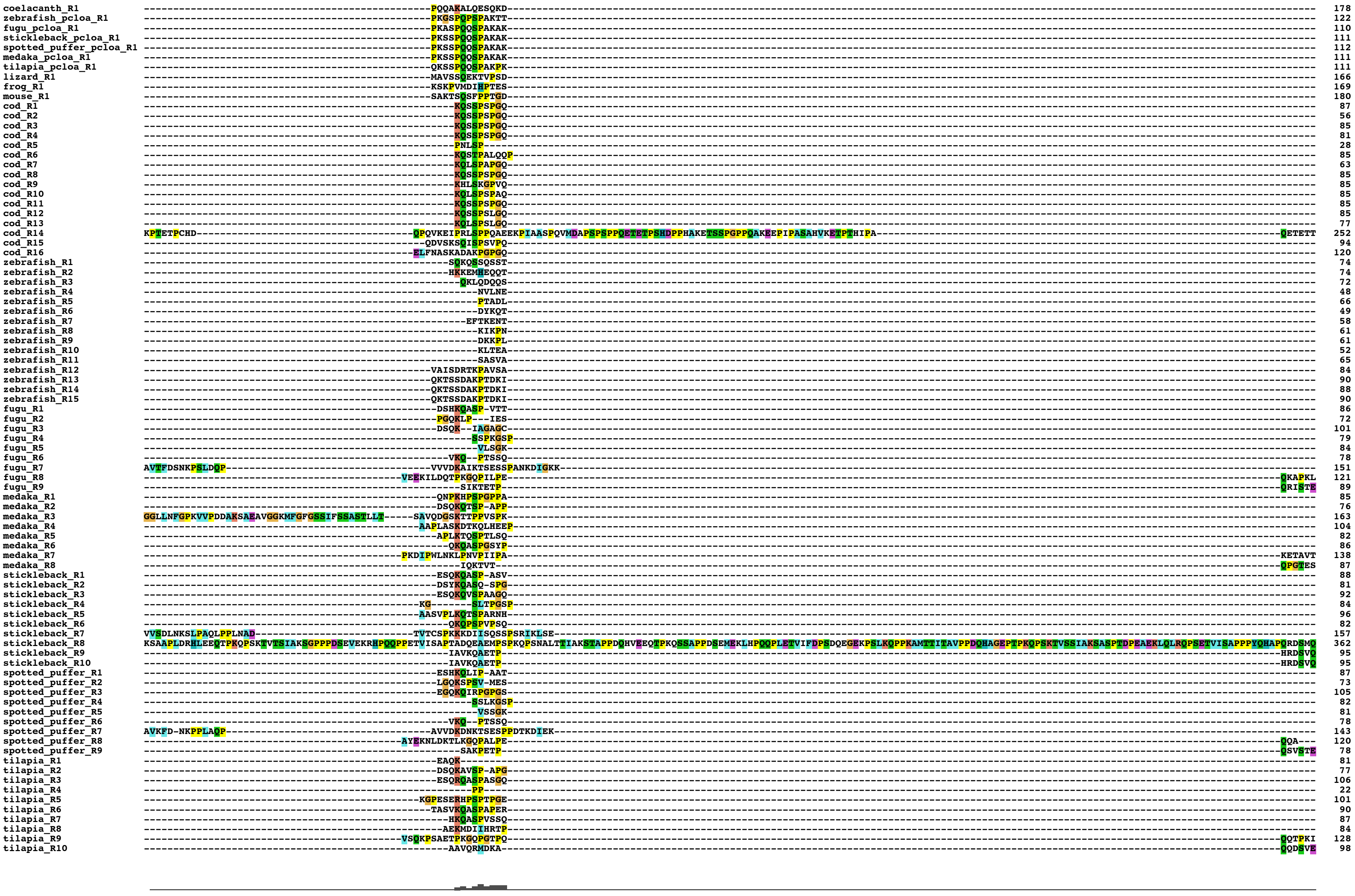
coelacanth_R1 178
zebrafish_pcloa_R1 122
fugu_pcloa_R1 110
stickleback_pcloa_R1 111
spotted_puffer_pcloa_R1 112
medaka_pcloa_R1 111
tilapia_pcloa_R1 111
lizard_R1 166
frog_R1 169
mouse_R1 180
cod_R1 87
cod_R2 56
cod_R3 85
cod_R4 81
cod_R5 28
cod_R6 85
cod_R7 63
cod_R8 85
cod_R9 85
cod_R10 85
cod_R11 85
cod_R12 85
cod_R13 77
cod_R14 252
cod_R15 94
cod_R16 120
zebrafish_R1 74
zebrafish_R2 74
zebrafish_R3 72
zebrafish_R4 48
zebrafish_R5 66
zebrafish_R6 49
zebrafish_R7 58
zebrafish_R8 61
zebrafish_R9 61
zebrafish_R10 52
zebrafish_R11 65
zebrafish_R12 84
zebrafish_R13 90
zebrafish_R14 88
zebrafish_R15 90
fugu_R1 86
fugu_R2 72
fugu_R3 101
fugu_R4 79
fugu_R5 84
fugu_R6 78
fugu_R7 151
fugu_R8 121
fugu_R9 89
medaka_R1 85
medaka_R2 76
medaka_R3 163
medaka_R4 104
medaka_R5 82
medaka_R6 86
medaka_R7 138
medaka_R8 87
stickleback_R1 88
stickleback_R2 81
stickleback_R3 92
stickleback_R4 84
stickleback_R5 96
stickleback_R6 82
stickleback_R7 157
stickleback_R8 362
stickleback_R9 95
stickleback_R10 95
spotted_puffer_R1 87
spotted_puffer_R2 73
spotted_puffer_R3 105
spotted_puffer_R4 82
spotted_puffer_R5 81
spotted_puffer_R6 78
spotted_puffer_R7 143
spotted_puffer_R8 120
spotted_puffer_R9 78
tilapia_R1 81
tilapia_R2 77
tilapia_R3 106
tilapia_R4 22
tilapia_R5 101
tilapia_R6 90
tilapia_R7 87
tilapia_R8 84
tilapia_R9 128
tilapia_R10 98

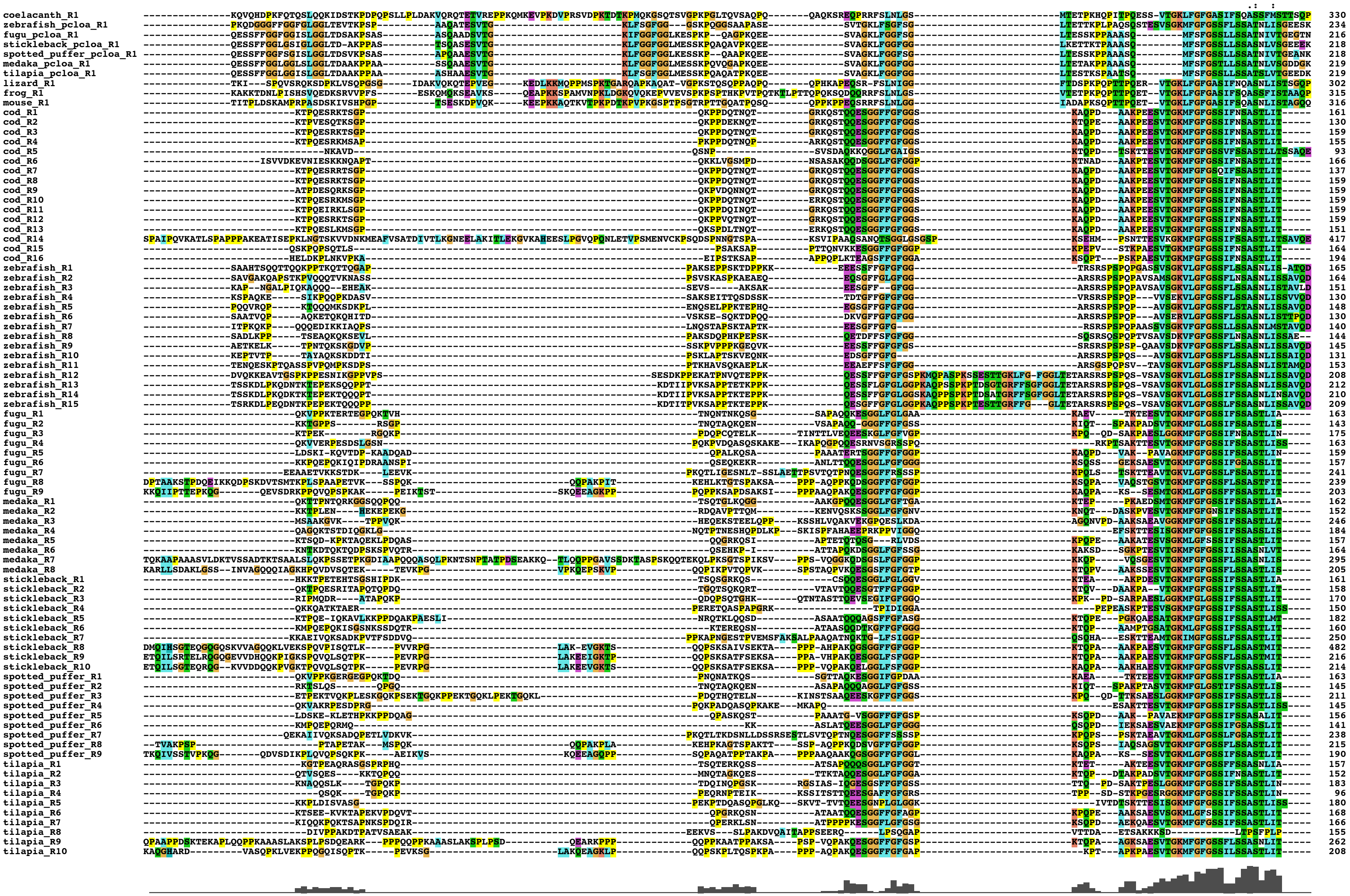

coelacanth_R1 330
zebrafish_pcloa_R1 234
fugu_pcloa_R1 216
stickleback_pcloa_R1 218
spotted_puffer_pcloa_R1 218
medaka_pcloa_R1 219
tilapia_pcloa_R1 219
lizard_R1 302
frog_R1 315
mouse_R1 316
cod_R1 161
cod_R2 130
cod_R3 159
cod_R4 155
cod_R5 93
cod_R6 166
cod_R7 137
cod_R8 159
cod_R9 159
cod_R10 159
cod_R11 159
cod_R12 159
cod_R13 151
cod_R14 417
cod_R15 164
cod_R16 194
zebrafish_R1 165
zebrafish_R2 164
zebrafish_R3 151
zebrafish_R4 130
zebrafish_R5 148
zebrafish_R6 130
zebrafish_R7 140
zebrafish_R8 144
zebrafish_R9 145
zebrafish_R10 131
zebrafish_R11 153
zebrafish_R12 208
zebrafish_R13 212
zebrafish_R14 210
zebrafish_R15 209
fugu_R1 163
fugu_R2 143
fugu_R3 175
fugu_R4 163
fugu_R5 159
fugu_R6 157
fugu_R7 241
fugu_R8 239
fugu_R9 203
medaka_R1 162
medaka_R2 152
medaka_R3 246
medaka_R4 184
medaka_R5 157
medaka_R6 164
medaka_R7 295
medaka_R8 205
stickleback_R1 161
stickleback_R2 158
stickleback_R3 170
stickleback_R4 150
stickleback_R5 182
stickleback_R6 160
stickleback_R7 250
stickleback_R8 482
stickleback_R9 216
stickleback_R10 214
spotted_puffer_R1 163
spotted_puffer_R2 145
spotted_puffer_R3 211
spotted_puffer_R4 145
spotted_puffer_R5 156
spotted_puffer_R6 141
spotted_puffer_R7 238
spotted_puffer_R8 215
spotted_puffer_R9 190
tilapia_R1 157
tilapia_R2 152
tilapia_R3 183
tilapia_R4 96
tilapia_R5 180
tilapia_R6 168
tilapia_R7 166
tilapia_R8 155
tilapia_R9 262
tilapia_R10 208

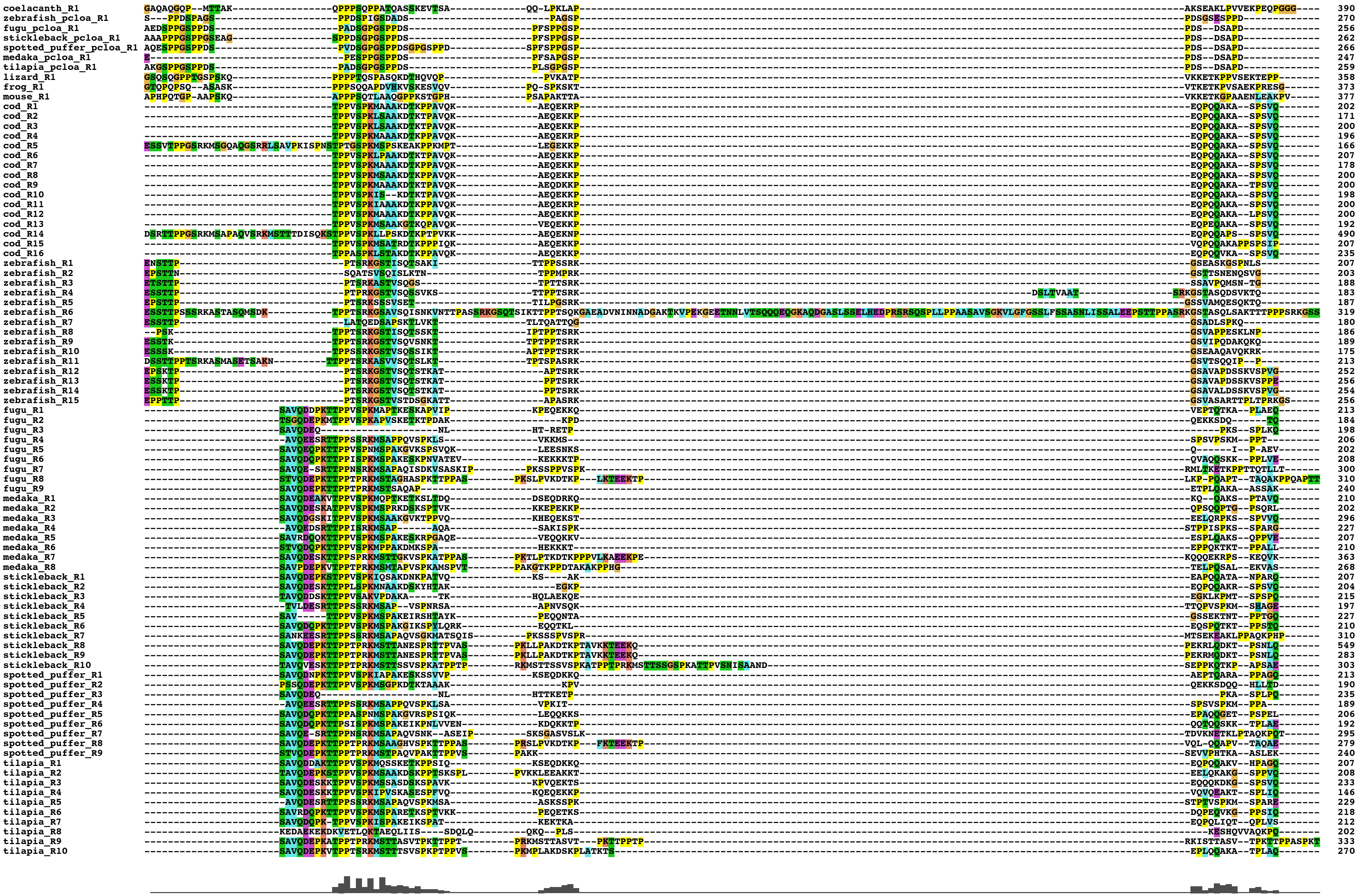

coelacanth_R1 390
zebrafish_pcloa_R1 270
fugu_pcloa_R1 256
stickleback_pcloa_R1 262
spotted_puffer_pcloa_R1 266
medaka_pcloa_R1 247
tilapia_pcloa_R1 259
lizard_R1 358
frog_R1 373
mouse_R1 377
cod_R1 202
cod_R2 171
cod_R3 200
cod_R4 196
cod_R5 166
cod_R6 207
cod_R7 178
cod_R8 200
cod_R9 200
cod_R10 198
cod_R11 200
cod_R12 200
cod_R13 192
cod_R14 490
cod_R15 207
cod_R16 235
zebrafish_R1 207
zebrafish_R2 203
zebrafish_R3 188
zebrafish_R4 183
zebrafish_R5 187
zebrafish_R6 319
zebrafish_R7 180
zebrafish_R8 186
zebrafish_R9 189
zebrafish_R10 175
zebrafish_R11 213
zebrafish_R12 252
zebrafish_R13 256
zebrafish_R14 254
zebrafish_R15 256
fugu_R1 213
fugu_R2 184
fugu_R3 198
fugu_R4 206
fugu_R5 202
fugu_R6 208
fugu_R7 300
fugu_R8 310
fugu_R9 240
medaka_R1 210
medaka_R2 202
medaka_R3 296
medaka_R4 227
medaka_R5 207
medaka_R6 210
medaka_R7 363
medaka_R8 268
stickleback_R1 207
stickleback_R2 204
stickleback_R3 215
stickleback_R4 197
stickleback_R5 227
stickleback_R6 210
stickleback_R7 310
stickleback_R8 549
stickleback_R9 283
stickleback_R10 303
spotted_puffer_R1 213
spotted_puffer_R2 190
spotted_puffer_R3 235
spotted_puffer_R4 189
spotted_puffer_R5 206
spotted_puffer_R6 192
spotted_puffer_R7 295
spotted_puffer_R8 279
spotted_puffer_R9 240
tilapia_R1 207
tilapia_R2 208
tilapia_R3 233
tilapia_R4 146
tilapia_R5 229
tilapia_R6 218
tilapia_R7 212
tilapia_R8 202
tilapia_R9 333
tilapia_R10 270

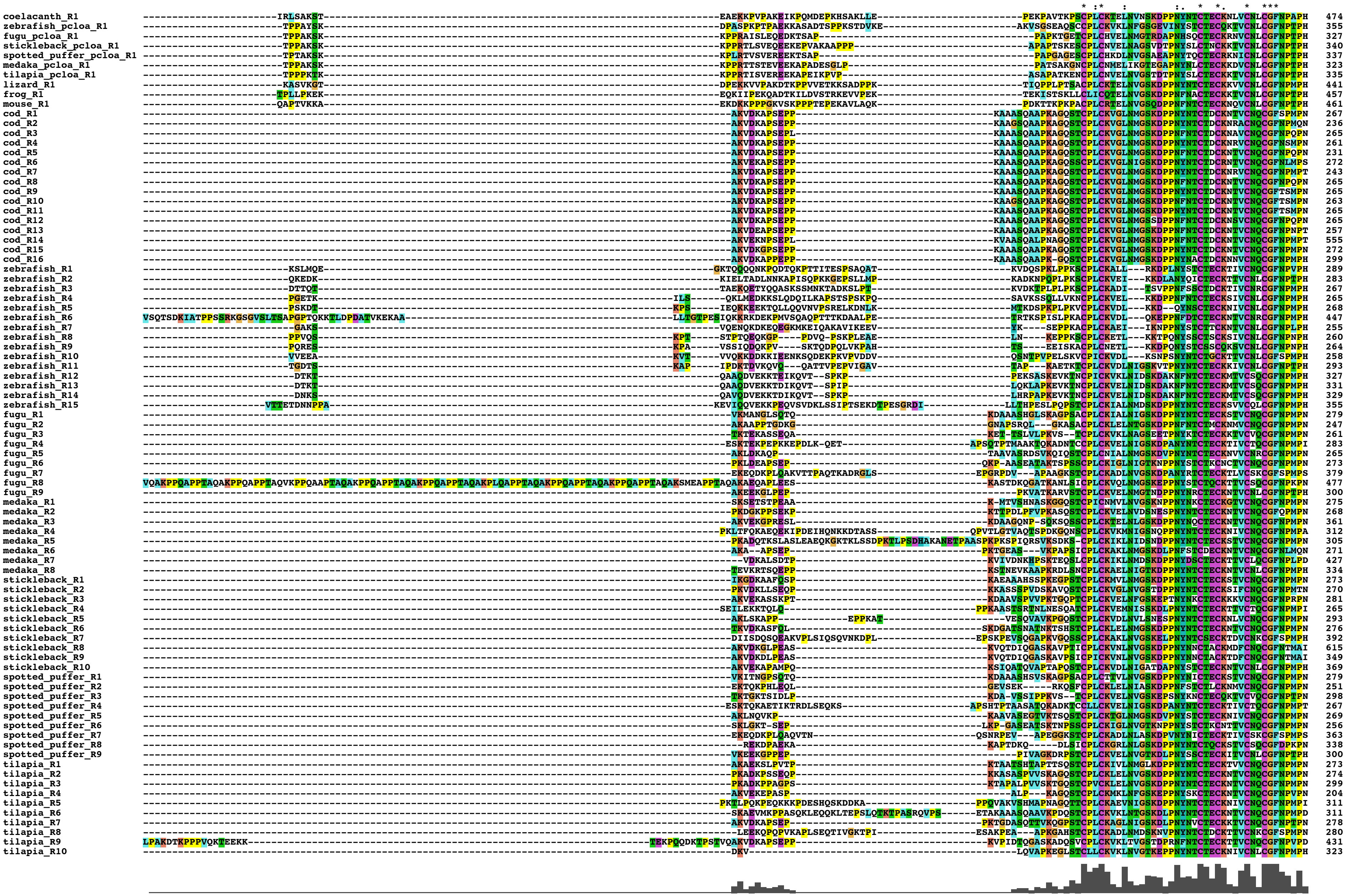
coelacanth_R1
zebrafish_pcloa_R1
fugu_pcloa_R1
stickleback_pcloa_R1
spotted_puffer_pcloa_R1
medaka_pcloa_R1
tilapia_pcloa_R1
lizard_R1
frog_R1
mouse_R1
cod_R1
cod_R2
cod_R3
cod_R4
cod_R5
cod_R6
cod_R7
cod_R8
cod_R9
cod_R10
cod_R11
cod_R12
cod_R13
cod_R14
cod_R15
cod_R16
zebrafish_R1
zebrafish_R2
zebrafish_R3
zebrafish_R4
zebrafish_R5
zebrafish_R6
zebrafish_R7
zebrafish_R8
zebrafish_R9
zebrafish_R10
zebrafish_R11
zebrafish_R12
zebrafish_R13
zebrafish_R14
zebrafish_R15
fugu_R1
fugu_R2
fugu_R3
fugu_R4
fugu_R5
fugu_R6
fugu_R7
fugu_R8
fugu_R9
medaka_R1
medaka_R2
medaka_R3
medaka_R4
medaka_R5
medaka_R6
medaka_R7
medaka_R8
stickleback_R1
stickleback_R2
stickleback_R3
stickleback_R4
stickleback_R5
stickleback_R6
stickleback_R7
stickleback_R8
stickleback_R9
stickleback_R10
spotted_puffer_R1
spotted_puffer_R2
spotted_puffer_R3
spotted_puffer_R4
spotted_puffer_R5
spotted_puffer_R6
spotted_puffer_R7
spotted_puffer_R8
spotted_puffer_R9
tilapia_R1
tilapia_R2
tilapia_R3
tilapia_R4
tilapia_R5
tilapia_R6
tilapia_R7
tilapia_R8
tilapia_R9
tilapia_R10

coelacanth_R1	LVE	477
zebrafish_pcloa_R1	LSE	358
fugu_pcloa_R1	LGE	330
stickleback_pcloa_R1	LGE	343
spotted_puffer_pcloa_R1	LGE	340
medaka_pcloa_R1	LGE	326
tilapia_pcloa_R1	LGE	338
lizard_R1	ITE	444
frog_R1	LTE	460
mouse_R1	LTE	464
cod_R1	VG-	269
cod_R2	KSE	239
cod_R3	SPG	268
cod_R4	ETG	264
cod_R5	VAE	234
cod_R6	KTE	275
cod_R7	KTE	246
cod_R8	STG	268
cod_R9	ETG	268
cod_R10	ETG	266
cod_R11	ETG	268
cod_R12	STG	268
cod_R13	VDK	260
cod_R14	SVE	558
cod_R15	TKE	275
cod_R16	SAA	302
zebrafish_R1	QTE	292
zebrafish_R2	QTE	286
zebrafish_R3	EKE	270
zebrafish_R4	QTE	268
zebrafish_R5	QNE	271
zebrafish_R6	ETE	450
zebrafish_R7	QTE	258
zebrafish_R8	QTD	263
zebrafish_R9	QIE	267
zebrafish_R10	QTK	261
zebrafish_R11	QTE	296
zebrafish_R12	QHE	330
zebrafish_R13	QTE	334
zebrafish_R14	QTE	332
zebrafish_R15	LAE	358
fugu_R1	VKE	282
fugu_R2	VTE	250
fugu_R3	MAE	264
fugu_R4	GK-	285
fugu_R5	EMV	268
fugu_R6	VSD	276
fugu_R7	VKE	382
fugu_R8	MVE	480
fugu_R9	TGA	303
medaka_R1	VKE	278
medaka_R2	VKE	271
medaka_R3	VSE	364
medaka_R4	GE-	314
medaka_R5	EAE	308
medaka_R6	ESE	274
medaka_R7	MSE	430
medaka_R8	TAV	337
stickleback_R1	VKE	276
stickleback_R2	VKE	273
stickleback_R3	ESE	284
stickleback_R4	GK-	267
stickleback_R5	ETA	296
stickleback_R6	VSE	279
stickleback_R7	LEK	395
stickleback_R8	VAE	618
stickleback_R9	VAE	352
stickleback_R10	TGA	372
spotted_puffer_R1	VKE	282
spotted_puffer_R2	ITE	254
spotted_puffer_R3	MSE	301
spotted_puffer_R4	AK-	269
spotted_puffer_R5	ET-	271
spotted_puffer_R6	VLE	259
spotted_puffer_R7	LKE	366
spotted_puffer_R8	VIE	341
spotted_puffer_R9	TGA	303
tilapia_R1	VNE	276
tilapia_R2	VKE	277
tilapia_R3	VKE	302
tilapia_R4	VSE	207
tilapia_R5	GE-	313
tilapia_R6	GTG	314
tilapia_R7	ETE	281
tilapia_R8	VTE	283
tilapia_R9	DSK	434
tilapia_R10	TAV	326
