# Supplementary material for: A Window into Domain Amplification Through Piccolo in Teleost Fish
Source: G3 (Bethesda). 2012 Nov 1;2(11):1325–39. doi: 10.1534/g3.112.003624 (PMC3484663; doi:10.1534/g3.112.003624)

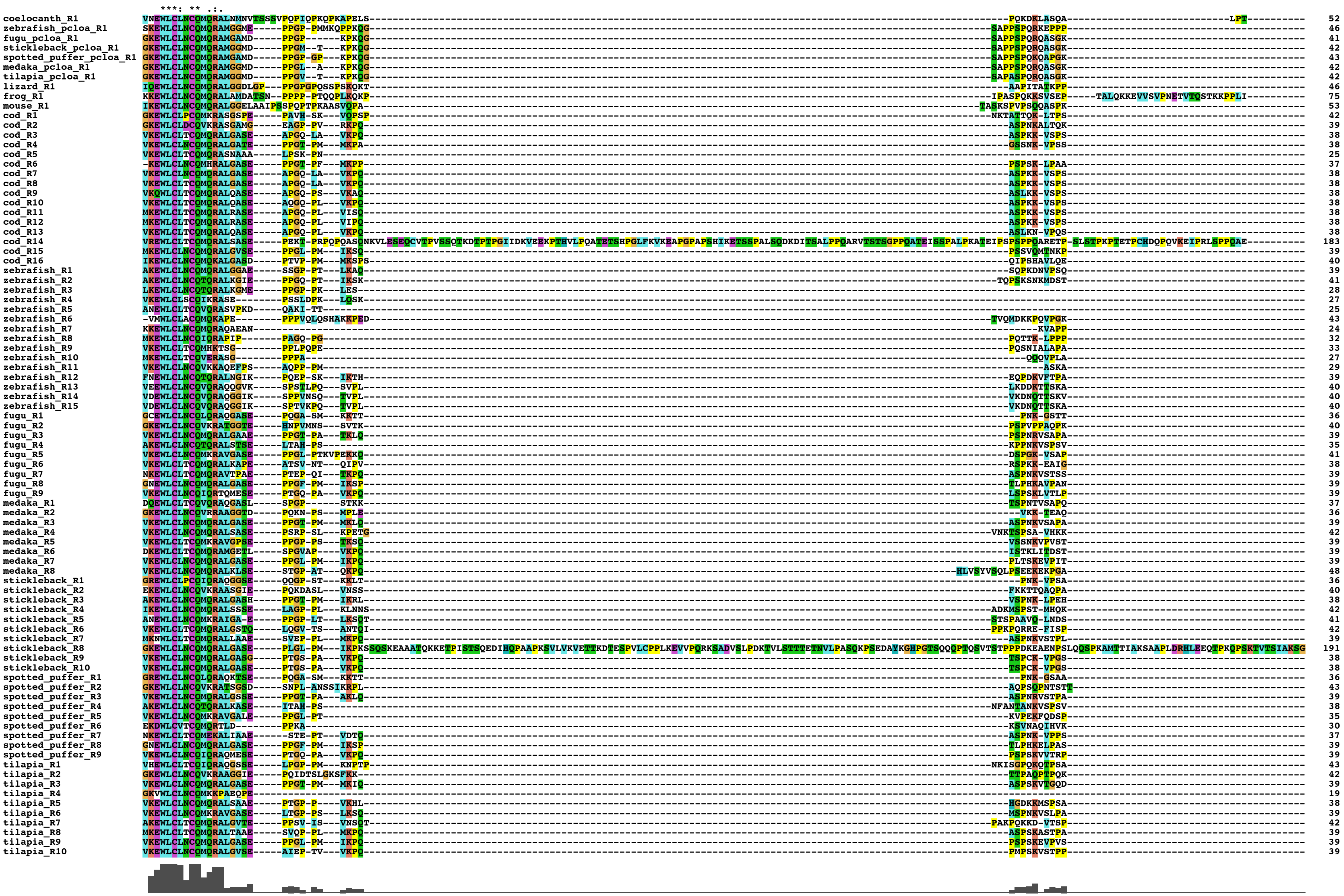

coelocanth_R1 52
zebrafish_pcloa_R1 46
fugu_pcloa_R1 41
stickleback_pcloa_R1 42
spotted_puffer_pcloa_R1 43
medaka_pcloa_R1 42
tilapia_pcloa_R1 42
lizard_R1 46
frog_R1 75
mouse_R1 53
cod_R1 42
cod_R2 39
cod_R3 38
cod_R4 38
cod_R5 25
cod_R6 37
cod_R7 38
cod_R8 38
cod_R9 38
cod_R10 38
cod_R11 38
cod_R12 38
cod_R13 38
cod_R14 183
cod_R15 39
cod_R16 40
zebrafish_R1 39
zebrafish_R2 41
zebrafish_R3 28
zebrafish_R4 27
zebrafish_R5 25
zebrafish_R6 43
zebrafish_R7 24
zebrafish_R8 32
zebrafish_R9 33
zebrafish_R10 27
zebrafish_R11 29
zebrafish_R12 39
zebrafish_R13 40
zebrafish_R14 40
zebrafish_R15 40
fugu_R1 36
fugu_R2 40
fugu_R3 39
fugu_R4 35
fugu_R5 41
fugu_R6 38
fugu_R7 39
fugu_R8 39
fugu_R9 39
medaka_R1 37
medaka_R2 36
medaka_R3 39
medaka_R4 42
medaka_R5 39
medaka_R6 39
medaka_R7 39
medaka_R8 48
stickleback_R1 36
stickleback_R2 40
stickleback_R3 38
stickleback_R4 42
stickleback_R5 41
stickleback_R6 42
stickleback_R7 39
stickleback_R8 191
stickleback_R9 38
stickleback_R10 38
spotted_puffer_R1 36
spotted_puffer_R2 43
spotted_puffer_R3 39
spotted_puffer_R4 38
spotted_puffer_R5 35
spotted_puffer_R6 30
spotted_puffer_R7 37
spotted_puffer_R8 39
spotted_puffer_R9 39
tilapia_R1 43
tilapia_R2 42
tilapia_R3 39
tilapia_R4 19
tilapia_R5 38
tilapia_R6 39
tilapia_R7 42
tilapia_R8 39
tilapia_R9 39
tilapia_R10 39

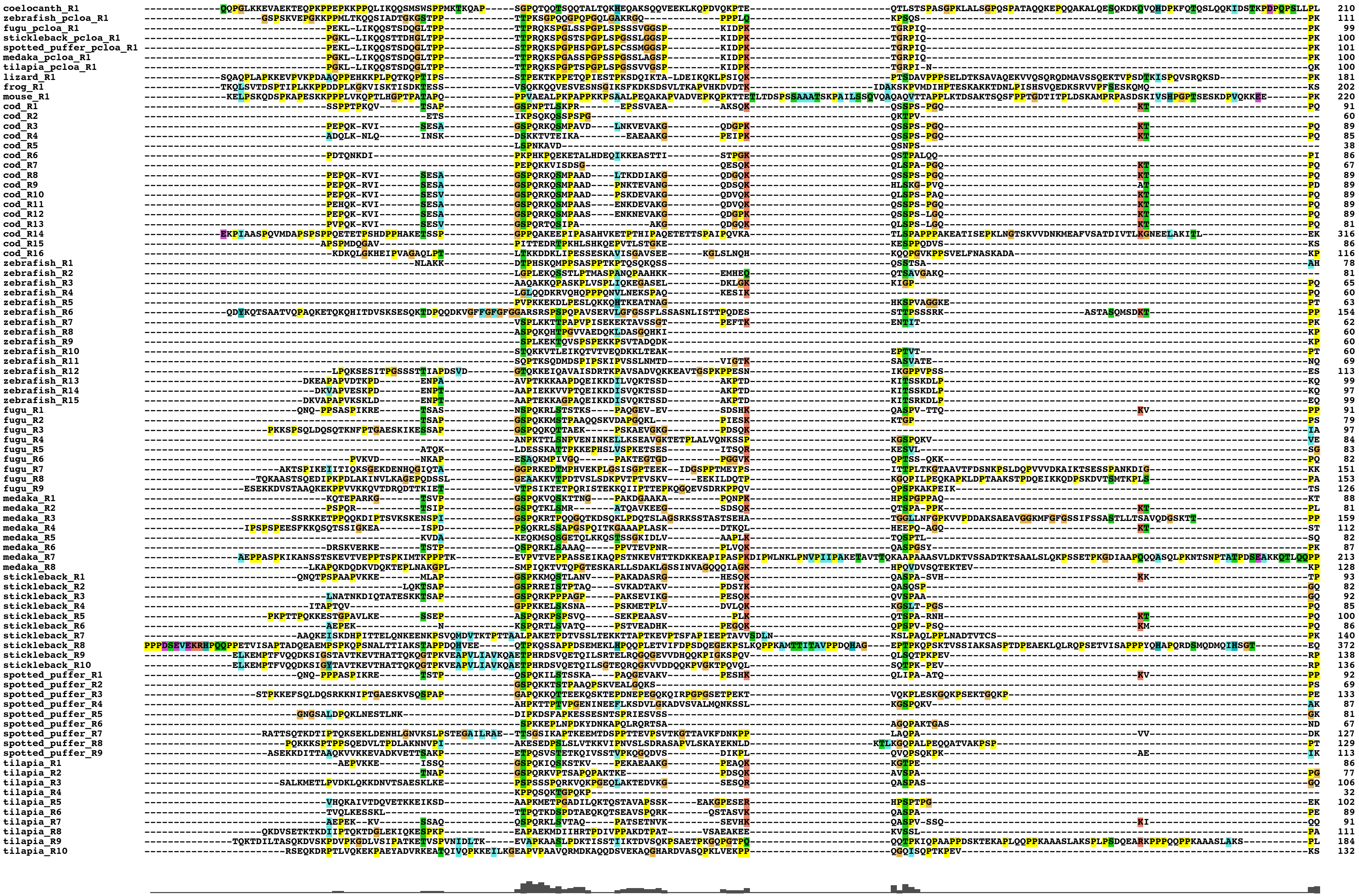
coelocanth_R1 210
zebrafish_pcloa_R1 111
fugu_pcloa_R1 99
stickleback_pcloa_R1 100
spotted_puffer_pcloa_R1 101
medaka_pcloa_R1 100
tilapia_pcloa_R1 100
lizard_R1 181
frog_R1 202
mouse_R1 220
cod_R1 91
cod_R2 60
cod_R3 89
cod_R4 85
cod_R5 38
cod_R6 86
cod_R7 67
cod_R8 89
cod_R9 89
cod_R10 89
cod_R11 89
cod_R12 89
cod_R13 81
cod_R14 316
cod_R15 86
cod_R16 116
zebrafish_R1 78
zebrafish_R2 81
zebrafish_R3 65
zebrafish_R4 60
zebrafish_R5 63
zebrafish_R6 154
zebrafish_R7 62
zebrafish_R8 60
zebrafish_R9 60
zebrafish_R10 60
zebrafish_R11 69
zebrafish_R12 113
zebrafish_R13 99
zebrafish_R14 97
zebrafish_R15 99
fugu_R1 91
fugu_R2 79
fugu_R3 97
fugu_R4 84
fugu_R5 83
fugu_R6 82
fugu_R7 151
fugu_R8 153
fugu_R9 126
medaka_R1 88
medaka_R2 81
medaka_R3 159
medaka_R4 112
medaka_R5 82
medaka_R6 87
medaka_R7 213
medaka_R8 128
stickleback_R1 93
stickleback_R2 82
stickleback_R3 92
stickleback_R4 85
stickleback_R5 100
stickleback_R6 86
stickleback_R7 140
stickleback_R8 372
stickleback_R9 138
stickleback_R10 136
spotted_puffer_R1 92
spotted_puffer_R2 69
spotted_puffer_R3 133
spotted_puffer_R4 87
spotted_puffer_R5 81
spotted_puffer_R6 67
spotted_puffer_R7 127
spotted_puffer_R8 129
spotted_puffer_R9 113
tilapia_R1 86
tilapia_R2 77
tilapia_R3 106
tilapia_R4 32
tilapia_R5 102
tilapia_R6 89
tilapia_R7 91
tilapia_R8 111
tilapia_R9 184
tilapia_R10 132

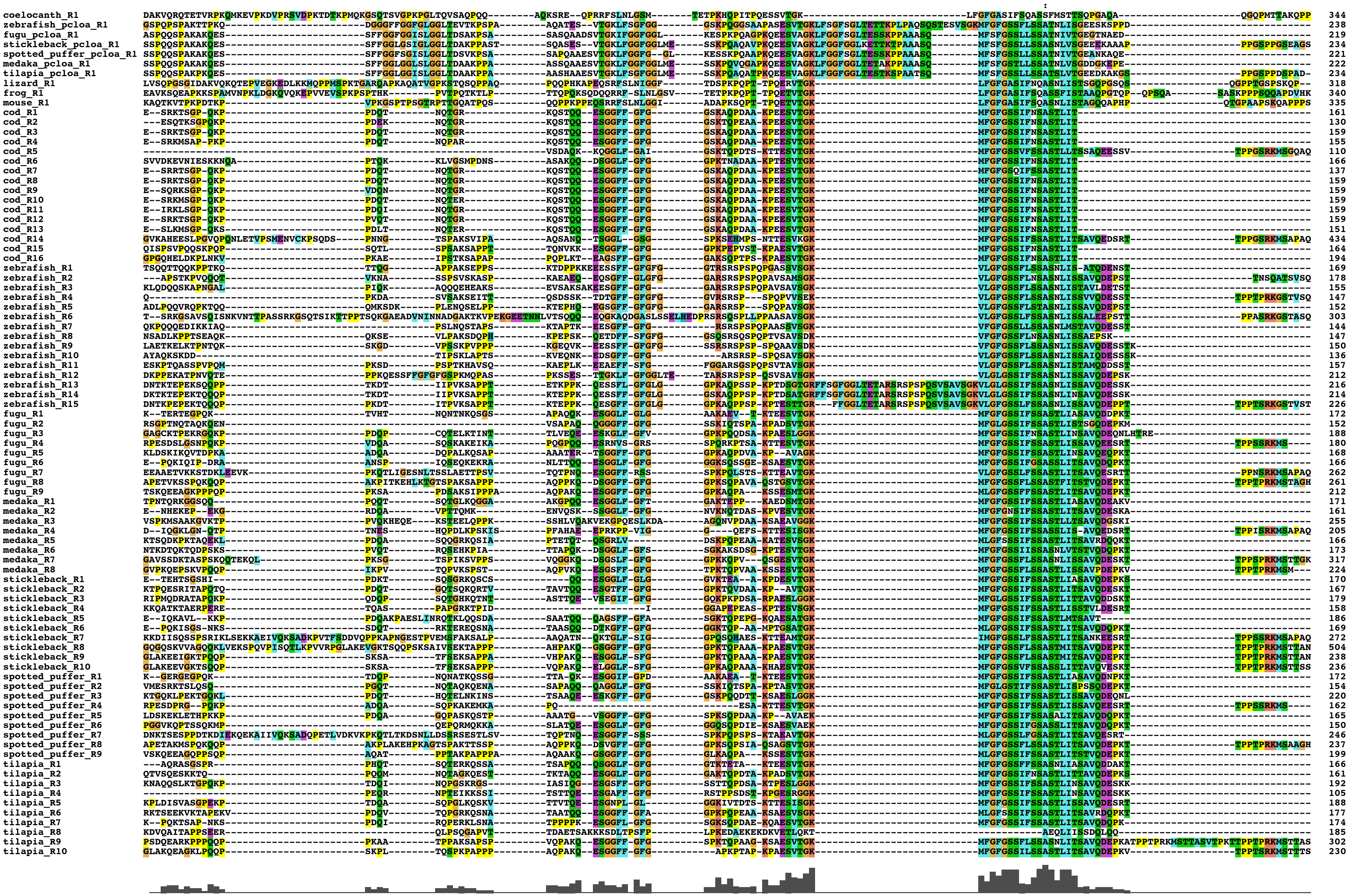

coelocanth_R1 344
zebrafish_pcloa_R1 238
fugu_pcloa_R1 219
stickleback_pcloa_R1 234
spotted_puffer_pcloa_R1 221
medaka_pcloa_R1 222
tilapia_pcloa_R1 234
lizard_R1 318
frog_R1 340
mouse_R1 335
cod_R1 161
cod_R2 130
cod_R3 159
cod_R4 155
cod_R5 110
cod_R6 166
cod_R7 137
cod_R8 159
cod_R9 159
cod_R10 159
cod_R11 159
cod_R12 159
cod_R13 151
cod_R14 434
cod_R15 164
cod_R16 194
zebrafish_R1 169
zebrafish_R2 178
zebrafish_R3 155
zebrafish_R4 147
zebrafish_R5 152
zebrafish_R6 303
zebrafish_R7 144
zebrafish_R8 147
zebrafish_R9 150
zebrafish_R10 136
zebrafish_R11 157
zebrafish_R12 212
zebrafish_R13 216
zebrafish_R14 214
zebrafish_R15 226
fugu_R1 172
fugu_R2 152
fugu_R3 188
fugu_R4 180
fugu_R5 168
fugu_R6 166
fugu_R7 262
fugu_R8 261
fugu_R9 212
medaka_R1 171
medaka_R2 161
medaka_R3 255
medaka_R4 205
medaka_R5 166
medaka_R6 173
medaka_R7 317
medaka_R8 224
stickleback_R1 170
stickleback_R2 167
stickleback_R3 179
stickleback_R4 158
stickleback_R5 186
stickleback_R6 169
stickleback_R7 272
stickleback_R8 504
stickleback_R9 238
stickleback_R10 236
spotted_puffer_R1 172
spotted_puffer_R2 154
spotted_puffer_R3 220
spotted_puffer_R4 162
spotted_puffer_R5 165
spotted_puffer_R6 150
spotted_puffer_R7 246
spotted_puffer_R8 237
spotted_puffer_R9 199
tilapia_R1 166
tilapia_R2 161
tilapia_R3 192
tilapia_R4 105
tilapia_R5 188
tilapia_R6 177
tilapia_R7 174
tilapia_R8 185
tilapia_R9 302
tilapia_R10 230

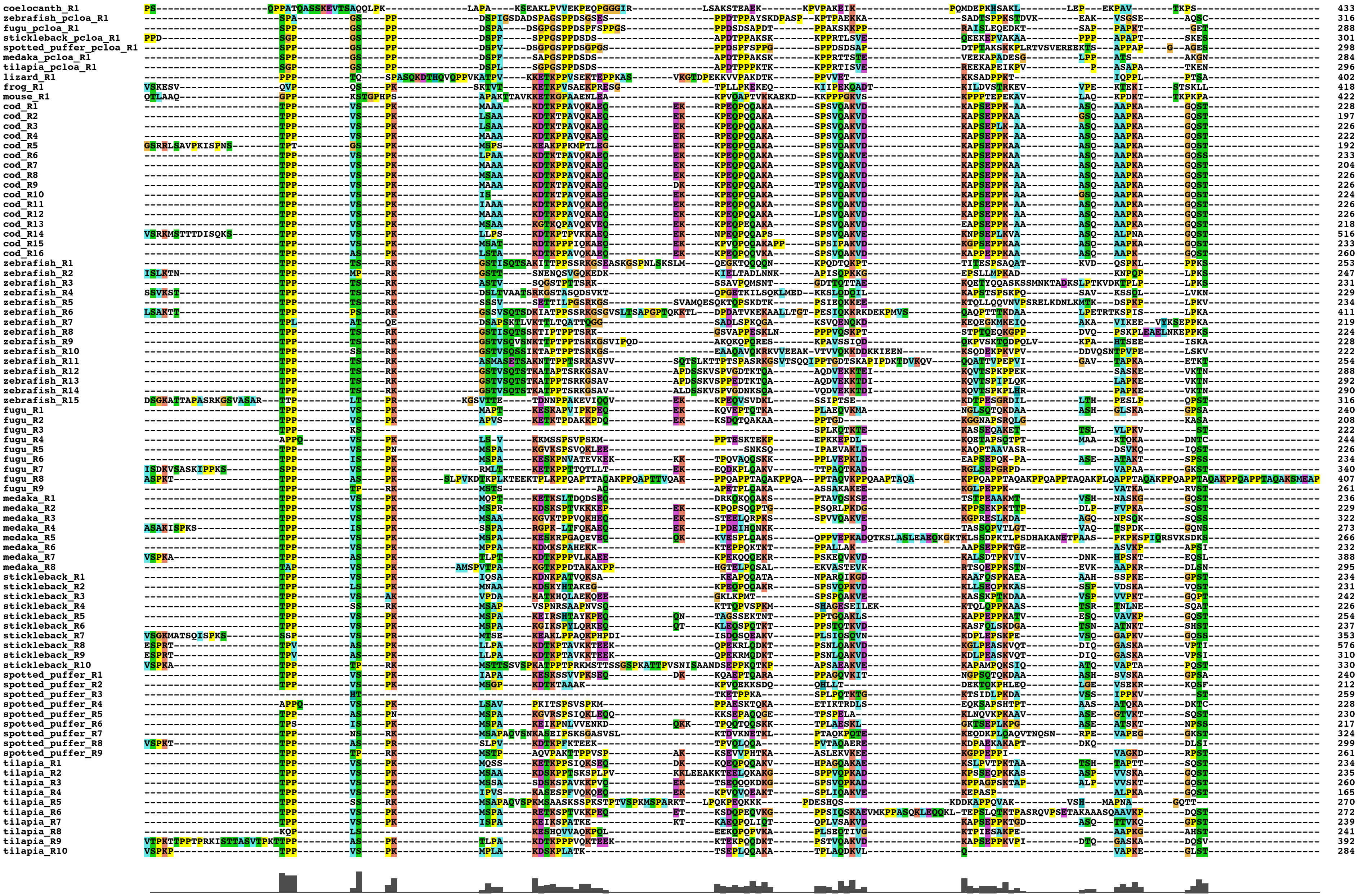
coelocanth_R1 433
zebrafish_pcloa_R1 316
fugu_pcloa_R1 288
stickleback_pcloa_R1 301
spotted_puffer_pcloa_R1 298
medaka_pcloa_R1 284
tilapia_pcloa_R1 296
lizard_R1 402
frog_R1 418
mouse_R1 422
cod_R1 228
cod_R2 197
cod_R3 226
cod_R4 222
cod_R5 192
cod_R6 233
cod_R7 204
cod_R8 226
cod_R9 226
cod_R10 224
cod_R11 226
cod_R12 226
cod_R13 218
cod_R14 516
cod_R15 233
cod_R16 260
zebrafish_R1 253
zebrafish_R2 247
zebrafish_R3 231
zebrafish_R4 229
zebrafish_R5 234
zebrafish_R6 411
zebrafish_R7 219
zebrafish_R8 224
zebrafish_R9 228
zebrafish_R10 222
zebrafish_R11 254
zebrafish_R12 288
zebrafish_R13 292
zebrafish_R14 290
zebrafish_R15 316
fugu_R1 240
fugu_R2 208
fugu_R3 222
fugu_R4 244
fugu_R5 226
fugu_R6 234
fugu_R7 340
fugu_R8 407
fugu_R9 261
medaka_R1 236
medaka_R2 229
medaka_R3 322
medaka_R4 273
medaka_R5 266
medaka_R6 232
medaka_R7 388
medaka_R8 295
stickleback_R1 234
stickleback_R2 231
stickleback_R3 242
stickleback_R4 226
stickleback_R5 254
stickleback_R6 237
stickleback_R7 353
stickleback_R8 576
stickleback_R9 310
stickleback_R10 330
spotted_puffer_R1 240
spotted_puffer_R2 212
spotted_puffer_R3 259
spotted_puffer_R4 228
spotted_puffer_R5 230
spotted_puffer_R6 217
spotted_puffer_R7 324
spotted_puffer_R8 299
spotted_puffer_R9 261
tilapia_R1 234
tilapia_R2 235
tilapia_R3 260
tilapia_R4 165
tilapia_R5 270
tilapia_R6 272
tilapia_R7 239
tilapia_R8 241
tilapia_R9 392
tilapia_R10 284

```
                                                                            :        :                .
coelocanth_R1            ------------------------------CPLCKTELNVNSKDPPNYNTCTECKNLVCNLCGFNPAPHLVE     475
zebrafish_pcloa_R1       -------------------------------CPLCKVKLNFGSGEVINYSTCTECQKTVCNLCGFNPTPHLSE    358
fugu_pcloa_R1            -------------------------------CPLCHVELNMGTRDAPNHSQCTECKRNVCNLCGFNPTPHLGE    330
stickleback_pcloa_R1     -------------------------------CPLCNVELNAGSVDTPNYSLCTNCKKTVCNLCGFNPTPHLGE    343
spotted_puffer_pcloa_R1  -------------------------------CPLCHKDLNVGSAEAPNYTQCTECRKNICNLCGFNPTPHLGE    340
medaka_pcloa_R1          -------------------------------CPLCNMELIKGTEGAPNYNLCTECKKDVCNLCGFNPTPHLGE    326
tilapia_pcloa_R1         -------------------------------CPLCNVELNVGSTDTPNYSLCTECKKTVCNLCGFNPTPHLGE    338
lizard_R1                -------------------------------CPLCKTELNVGSKDPPNYNTCTECKKVVCNLCGFNPMPHITE    444
frog_R1                  -------------------------------CLICQTELNVGSKDPPNFNACTECKKTVCNLCGFNPTPHLTE    460
mouse_R1                 -------------------------------CPLCRTELNVGSQDPPNFNTCTECKNQVCNLCGFNPTPHLTE    464
cod_R1                   -------------------------------CPLCKVGLNMGSKDPPNYNTCTDCKNTVCNQCGFSPMPNVG-    269
cod_R2                   -------------------------------CPLCKVGLNMGSKDPPNYNTCTDCKNRACNQCGFNPMQNKSE    239
cod_R3                   -------------------------------CPLCKVGLNMGSKDPPNFNTCTDCKNAVCNQCGFNPQPNSPG    268
cod_R4                   -------------------------------CPLCKVGLNMGSKDPPNYNTCTDCKNRVCNQCGFNSMPNETG    264
cod_R5                   -------------------------------CPLCKVGLNMGSKDPPNFNTCTDCKNTVCNQCGFNPQPNVAE    234
cod_R6                   -------------------------------CPLCKVGLNIGSKDPPNYNTCTDCRNTVCNQCGFNLMPSKTE    275
cod_R7                   -------------------------------CPLCKVGLNMGSKDPPNYNTCTDCKNRVCNQCGFNPMPTKTE    246
cod_R8                   -------------------------------CPLCKVGLNMGSKDPPNFNTCTDCKNTVCNQCGFNPQPNSTG    268
cod_R9                   -------------------------------CPLCKVGLNMGSKDPPNYNTCTDCKNTVCNQCGFTSMPNETG    268
cod_R10                  -------------------------------CPLCKVGLNMGSKDPPNFNTCTDCKNTVCNQCGFTSMPNETG    266
cod_R11                  -------------------------------CPLCKVGLNMGSKDPPNYNTCTDCKNTVCNQCGFTSMPNETG    268
cod_R12                  -------------------------------CPLCKVGLNMGSSDPPNFNTCTDCKNSVCNQCGFNPQPNSTG    268
cod_R13                  -------------------------------CPLCKVGLNMGSKDPPNFNTCTDCKNTVCNQCGFNPNPTVDK    260
cod_R14                  -------------------------------CPLCKVGLNMGSKDPPNFNTCTDCKNTVCNQCGFNPMPTSVE    558
cod_R15                  -------------------------------CPLCKVGLNMGSKDPPNYNTCTDCKNTVCNQCGFNPMPNTKE    275
cod_R16                  -------------------------------CPLCKVGLNMGSKDPPNYNACTDCKNNVCNQCGFNPMPHSAA    302
zebrafish_R1             -------------------------------CPLCKALLR---KDPLNYSTCTECKTIVCNQCGFNPVPHQTE    292
zebrafish_R2             -------------------------------CPLCKVEIK---KDLANYQICTECKTTVCNLCGFNPTPHQTE    286
zebrafish_R3             -------------------------------CPLCKADIT---SVPPNFSSCTDCKTIVCNRCGFNPMPHEKE    270
zebrafish_R4             -------------------------------CPLCKVELK---KDPPNFNTCTECKSIVCNLCGFNPMPHQTE    268
zebrafish_R5             -------------------------------CPLCKVDLK---KD--QYNSCTECKNIVCNLCGFNPMPHQNE    271
zebrafish_R6             -------------------------------CPLCKVDLQ---KEPPNFDTCTECKNTVCNRCGFNPMPHETE    450
zebrafish_R7             -------------------------------CPLCKAEII---KNPPNYSTCTTCKNTVCNLCGFNPLPHQTE    258
zebrafish_R8             -------------------------------CPLCKETLK---KTPQNYSSCTSCKSIVCNLCGFNPNPHQTD    263
zebrafish_R9             -------------------------------CPLCNETLK---KDPQNYSTCSSCQKSVCNLCGFNPNPHQIE    267
zebrafish_R10            -------------------------------CPICKVDLK---SNPSNYNTCTGCKTTVCNLCGFSPMPHQTK    261
zebrafish_R11            -------------------------------CPLCKVDLNIGSKVTPNYNTCTECKKIVCNLCGFNPTPHQTE    296
zebrafish_R12            -------------------------------CPICKVKLNIDSKDAKNFNTCTECKMTVCSQCGFNPMPHQHE    330
zebrafish_R13            -------------------------------CPLCKVELNIDSKDAKNFNTCTECKMTVCSQCGFNPMPHQTE    334
zebrafish_R14            -------------------------------CPLCKVELNIDSKDAKNFNTCTECKMTVCSQCGFNPMPHQTE    332
zebrafish_R15            -------------------------------CPLCKIALNMDSKDPPNYNTCTECKSVVCQLCGFNPMPHLAE    358
fugu_R1                  -------------------------------CPLCKIALNIGSKDPPNYNTCTECKSTVCNQCGFNPMPNVKE    282
fugu_R2                  -------------------------------CPLCKLELNTGSKDPPNFNTCTMCKNMVCNQCGFNPMPNVTE    250
fugu_R3                  -------------------------------CPLCKVKLNAGSEETPNYKTCTECKKTVCVQCGFNPMPNMAE    264
fugu_R4                  -------------------------------CPLCKVELNIGSKDPANYNTCTECKTIVCTQCGFNPMP-IGK    285
fugu_R5                  -------------------------------CPLCNIALNMGSKDVPNYNTCTECKNVVCNRCGFNPMPNEMV    268
fugu_R6                  -------------------------------CPLCKIGLNIGTKNPPNYSTCTKCNCTVCNQCGFNPMPNVSD    276
fugu_R7                  -------------------------------CPLCKADLNVGSKDPANYRTCTECKTLVCSKCGFSPMPSVKE    382
fugu_R8                  PTAQAKAEQAPLEESKASTDKQGATKANLSICPLCKVQLNMGSKEPPNYSTCTQCKTTVCSQCGFNPKPNMVE    480
fugu_R9                  -------------------------------CPLCKVELNMGTNDPPNYNRCTECKNTVCNLCGFNPTPHTGA    303
medaka_R1                -------------------------------CPICNMVLNVGSKNPPNYNKCTECKGTVCNQCGFNPMPNVKE    278
medaka_R2                -------------------------------CPLCKVELNVDSNESPNYNTCTECKNTVCNQCGFQPMPNVKE    271
medaka_R3                -------------------------------CPLCKTELNLGSKDPPNYNQCTECKNTVCNQCGFNPMPNVSE    364
medaka_R4                -------------------------------CPLCKVKMNIGSNQPPNYNTCTECKNIVCNQCGFNPMP-AGE    314
medaka_R5                -------------------------------CPLCKIKLNIDSNDPPNYNTCTECKSTVCNQCGFNPMPNEAE    308
medaka_R6                -------------------------------CPLCKAKLNMGSKDLPNFSTCDECKNTVCNQCGFNLMQNESE    274
medaka_R7                -------------------------------CPLCKIKLNMDSKDPPNYDSCTECKTTVCLQCGFNPLPDMSE    430
medaka_R8                -------------------------------CPLCKAELNIGTKDPPNYNTCTECKNTVCNLCGFNPMPHTAV    337
stickleback_R1           -------------------------------CPLCKMVLNMGSKDPPNYNTCTECKSTVCNQCGFNPMPNVKE    276
stickleback_R2           -------------------------------CPLCKVGLNVGSTDPPNYNTCTECKSIFCNQCGFNPMTNVKE    273
stickleback_R3           -------------------------------CPLCKVELNFGSKEPTNYNKCTECKKKVCNQCGFNPRPNESE    284
stickleback_R4           -------------------------------CPLCKVEMNISSKDLPNYNTCTECKTTVCTQCGFNPMP-IGK    267
stickleback_R5           -------------------------------CPLCKVDLNVLSNESPNYNTCTECKNLVCNQCGFNPMPNETA    296
stickleback_R6           -------------------------------CPLCKLELNMGSKDPPNYNTCTECKNTVCNQCGFNPMPNVSE    279
stickleback_R7           -------------------------------CPLCKAKLNVGSKELPNYNTCSECKTDVCNKCGFSPMPHLEK    395
stickleback_R8           -------------------------------CPLCKVNLNVGSKDPPNYNNCTACKMDFCNQCGFNTMAIVAE    618
stickleback_R9           -------------------------------CPICKVNLNVGSKDPPNYNNCTACKTDFCNQCGFNTMAIVAE    352
stickleback_R10          -------------------------------CPLCKVDLNIGATDAPNYSTCTECKNTVCNLCGFNPMPHTGA    372
spotted_puffer_R1        -------------------------------CPLCTTVLNVGSKDPPNYNICTECKSTVCNQCGFNPMPNVKE    282
spotted_puffer_R2        -------------------------------CPLCKLELNIASKDPPNFSTCTLCKNMVCNQCGFNPMPNITE    254
spotted_puffer_R3        -------------------------------CPLCKVELNVGSKEPSNYKNCTECQKTVCVQCGFNPTPNMSE    301
spotted_puffer_R4        -------------------------------CLLCKVELNIGSKDPANYNTCTECKTIVCTQCGFNPMPTAK-    269
spotted_puffer_R5        -------------------------------CPLCKTGLNMGSKDVPNYSTCTECKNIVCNQCGFNPMPNET-    271
spotted_puffer_R6        -------------------------------CPLCKIGLNVGTKNPPNYSTCTKCNTTVCNQCGFNPMPNVLE    259
spotted_puffer_R7        -------------------------------CPLCKADLNLASKDPVNYNICTECKTIVCSKCGFSPMPSLKE    366
spotted_puffer_R8        -------------------------------CPLCKGRLNLGSKDPPNYNTCTQCKSTVCSQCGFDPKPNVIE    341
spotted_puffer_R9        -------------------------------CPLCKVELNVGTKDLPNYSSCTECKITVCNLCGFNPTPHTGA    303
tilapia_R1               -------------------------------CPLCKIVLNMGLKDPPNYNTCTECKTVVCNQCGFNPMPNVNE    276
tilapia_R2               -------------------------------CPLCKLELNVGSKDPPNYNTCTECKNTVCNQCGFNPMPNVKE    277
tilapia_R3               -------------------------------CPVCKLELNVGSKDPPNYNTCTECKNTVCNQCGFNPMPNVKE    302
tilapia_R4               -------------------------------CPLCKMKLNFGSKEPPNYSKCTECKNTVCNQCGFNPVPNVSE    207
tilapia_R5               ------------------------------CPLCKAEVNIGSKDPPNYNTCTECKNIVCNQCGFNPMP-IGE     311
tilapia_R6               -------------------------------CPLCKVKLNTGSKDLPNYNTCTECKNTVCNQCGFNPMPDGTG    314
tilapia_R7               -------------------------------CPLCKAGLNIGSKDLPNYNVCTECKKTVCNQCGFNPTPNETE    281
tilapia_R8               -------------------------------CPLCKADLNMDSKNVPNYNTCTDCKTTVCNKCGFSPMPNVTE    283
tilapia_R9               -------------------------------CPLCKVKLTVGSTDPRNFNTCTECKTTVCNQCGFNPVPDDSK    434
tilapia_R10              -------------------------------CLLCKVKLNVGTKEPPNYNTCTECKNIVCNLCGFNPMPHTAV    326
```

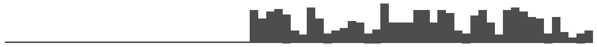

Supplement: Supporting Information [file supp_2.11.1325_FigureS17.pdf]
