# Supplementary material for: A Window into Domain Amplification Through Piccolo in Teleost Fish
Source: G3 (Bethesda). 2012 Nov 1;2(11):1325–39. doi: 10.1534/g3.112.003624 (PMC3484663; doi:10.1534/g3.112.003624)
Supplement: Supporting Information [file supp_2.11.1325_TableS3.pdf]

Table S3 Oligonucleotides used in this study

A list of oligonucleotides used in the study for 1) characterization of the splicing of zebrafish *pclob* by RT-PCR, 2) verify the identification of cod and green spotted puffer genomic samples, 3) to isolation of PCR products from tilapia, fugu, and cod repeat exons to correct sequencing errors and fill in missing WGS data, 4) to amplify from cDNA templates for the construction of cRNA probes for in situ hybridization.

primer	Sequence 5'- 3'	purpose
4721	CWCCCAAAGCYRRNATTCTWAAYTAAACTA	amplify and sequence mitochondrial genome D-Loop region
4722	CATAGTGGGGTATCTAATCCCAGTTTG	amplify and sequence mitochondrial genome D-Loop region
4703	GCCTCACTGAAGAATGTCCCTCA	amplify and sequence cod zinc finger repeat 13
4704	GGTGACACACTGTTCAGATTCAAGC	amplify and sequence cod zinc finger repeat 13
4705	GTCTCAGAACCAGGGCCAGGA	amplify and sequence spotted puffer zinc finger repeat 2/3
4606	CAGAAACCTCATTTAGAGCAGTTAGGGG	amplify and sequence spotted puffer zinc finger repeat 2/3
4707	CCTGATTTCAAATAAGTTGGGACAGTATGTAAGAG	amplify and sequence spotted puffer zinc finger repeat 8
4708	GTGGATTTAGTCCTATGCCAAGCCTA	amplify and sequence spotted puffer zinc finger repeat 8
4717	AGCCTTCTGCAGAGACTCCCAAGGG	amplify and sequence tilapia zinc finger repeat 9
4718	CCTCCGTTTTCTGGACAGGCGGTGG	amplify and sequence tilapia zinc finger repeat 9
4768	ACCTTGGTGGGAAGTCCTGGT	amplify and sequence spotted puffer zinc finger repeat 4
4769	CTTTGTCTTTACAGTCCTGAGC	amplify and sequence spotted puffer zinc finger repeat 4
4853	GCTCTGTCCAGTGTGCAAGACTG	amplify and sequence *bassoon b* cDNA fragment
4854	CAGCTGGCTTTTGCTGCTGAGG	amplify and sequence *bassoon b* cDNA fragment
4855	CACCATAGCAGCTGCCGTTACAC	amplify and sequence *piccolo b* cDNA fragment
4856	TGCTGCAGCCTGGTCTTGTTC	amplify and sequence *piccolo b* cDNA fragment
4582	CCTCTGCCAAGATCACAACACCC	amplify zebrafish zinc finger repeat R1|R2 junction from cDNA
4583	CTGACTGTTCTTGTTCAGCCTCAGC	amplify zebrafish zinc finger repeat R1|R2 junction from cDNA
4598	GGACCTTGCCAACTACCAGATCTG	amplify zebrafish zinc finger repeat R2|3, R3|4 junctions from cDNA
4605	AGACTGCAACTTAGGATCTAGACTTGAAG	amplify zebrafish zinc finger repeat R2|3, R3|4 junctions from cDNA
4599	CAGCTGTTAAGTCCTCTCAGCTTCTTG	amplify zebrafish zinc finger repeat R4|5, R5|6 junctions from cDNA
4604	CGAGAACTAGACGGTGTTGTGGAGG	amplify zebrafish zinc finger repeat R4|5, R5|6 junctions from cDNA
4597	CCCATGGTCAGTCAGGCACAG	amplify zebrafish zinc finger repeat R6|7 junction from cDNA
4584	GGAGATGGCGATCTAGAGCGTCC	amplify zebrafish zinc finger repeat R6|7 junction from cDNA
4596	GAATCCACCAAACTATAGCACATGTACTAC	amplify zebrafish zinc finger repeat R7|8, R8|9 junctions from cDNA
4603	GTGGAGATGCTGGTGCAAGTGC	amplify zebrafish zinc finger repeat R7|8, R8|9 junctions from cDNA
4595	CAGAACTACAGCACTTGTAGTTCATGCC	amplify zebrafish zinc finger repeat R9|10, R10|11 junctions from cDNA
4602	GGTTGAGATGCTTTGCTAGCCATTGG	amplify zebrafish zinc finger repeat R9|10, R10|11 junctions from cDNA
4594	GTGGATCTCAATATAGGTTCTAAGGTCACTCC	amplify zebrafish zinc finger repeat R11|12, R12|13 junctions from cDNA
4601	CCTGATCAGGTGCAGCCTTCTTC	amplify zebrafish zinc finger repeat R11|12, R12|13 junctions from
