# Supplementary material for: A Window into Domain Amplification Through Piccolo in Teleost Fish
Source: G3 (Bethesda). 2012 Nov 1;2(11):1325–39. doi: 10.1534/g3.112.003624 (PMC3484663; doi:10.1534/g3.112.003624)
Supplement: Supporting Information [file supp_2.11.1325_FigureS3.pdf]

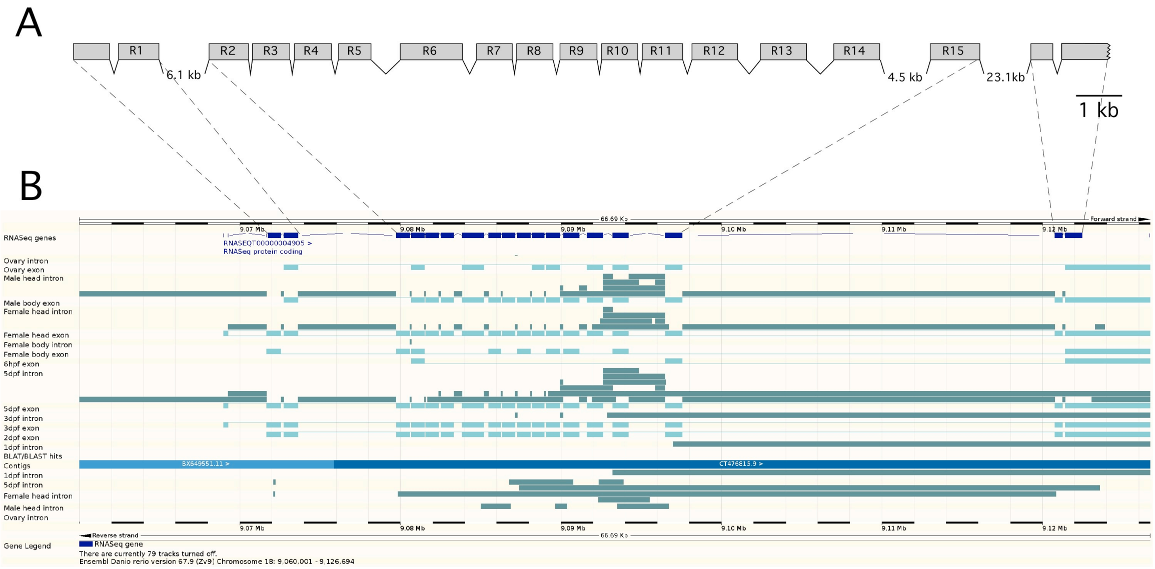

Figure S3 RNAseq DNA of zebrafish *pclob* repeated zinc finger exons. A) Diagram of the exon structure of the repeated exons encoding zinc finger domains of zebrafish *pclob*. The drawing is to scale except for the 3 large exons, the sizes of which are indicated in the drawing. A 1 kb scale bar is present on the right. B) A contig view obtained from Ensembl v67 of the genomic organization of the zinc finger exon region of *pclob* (chr18: 9,060,001- 9,126,694). RNAseq predicted gene structure for *pclob* (RNAseqT000004905) is identical to the manual annotation in the zinc finger repeat exon region. Note that translation of the T000004905 transcript ends in the Ensembl display because of an error in the genomic sequence (see materials and methods for details). The consensus exon structures determined from RNAseq of sample of various age and/or body section are shown. Also shown are the intron splicing patterns utilized in each sample based on RNAseq data crossing splice junctions.
