# Supplementary material for: A Window into Domain Amplification Through Piccolo in Teleost Fish
Source: G3 (Bethesda). 2012 Nov 1;2(11):1325–39. doi: 10.1534/g3.112.003624 (PMC3484663; doi:10.1534/g3.112.003624)
Supplement: Supporting Information [file supp_2.11.1325_FigureS11.pdf]

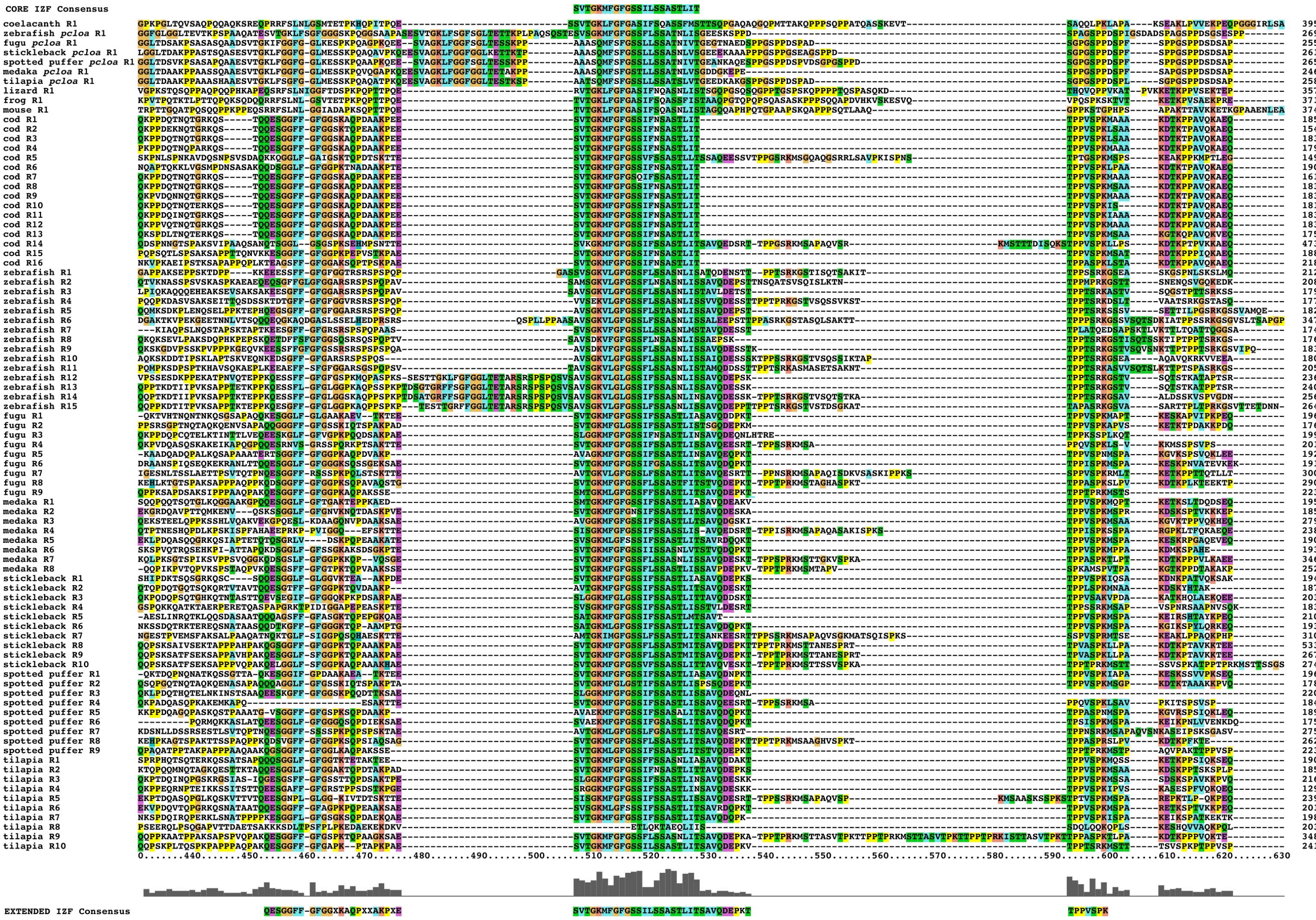

Figure S11 Sequence alignments of the IZF domain of *piccolo* genes. An alignment of central region of exons encoding the Inter Zinc Finger domain (IZF). *piccolo* repeat exon sequences were aligned using MUSCLE and formatting of the alignment figure was performed using Clustal X as described in methods. A bar graph below represents the level of conservation. A core IZF domain consensus sequence is positioned above the alignment and an extended IZF domain consensus sequence is positioned below the alignment.
